# Supplementary material for: Coursing hyenas and stalking lions: The potential for inter- and intraspecific interactions
Source: PLoS One. 2023 Feb 3;18(2):e0265054. doi: 10.1371/journal.pone.0265054 (PMC9897591; doi:10.1371/journal.pone.0265054)
Supplement: S1 Appendix — (PDF) [file pone.0265054.s001.pdf]

S1 Appendix. Additional details on study sites and data collection.

Etosha National Park

Etosha National Park, a 22,270km^2 fenced reserve in northern Namibia (Fig. 1), is classified as semi-arid savanna [1], with perennial water available only natural artesian springs or from pumped boreholes [2]. The 9000km^2 Etosha site occupied the central regions of Okaukeujo and Halali, and included an area outside the southeastern boundary of the park that a collared individual had dispersed to. Seasonal rainfall occurs mainly between November and April, with the highest monthly rainfall in January and February [3]. The area is interspersed with dolomite inselberge and sandy bushveld, and is comprised mainly of grasslands, short-grass plains, shrub-land, dwarf-shrub and mixed tree savanna, including Mopani (*Colophospermum mopane*). [4].

Chobe/Linyanti River System

The Chobe National Park, a 11,700km^2 unfenced reserve in northeastern Botswana, and the Linyanti Conservancy, a 1660km^2 community managed wildlife area within the Chobe Enclave surrounded by the Chobe National Park (Fig. 1) form part of the core component of the Kavango-Zambezi (KAZA) Transfrontier Conservation Area. The 7500km^2 Botswana site occupies the floodplains of the northern Kalahari bounded by the waters of the Chobe and Linyanti Rivers, complete with papyrus swamps, lagoons and marshes. The Linyanti-Chobe Rivers, fed by the Kwando and Zambezi rivers, and the Cubango River that feeds into the Okavango Delta are the main sources of permanent water for this ecosystem [5]. Seasonal rainfall occurs mainly between November and March with a mainly dry season from April to October. The vegetation of the area is highly varied with riparian and mopane woodlands,

Baikiaea forests (*Baikiaea plurijugn*), open grasslands and mixed Acacia savanna, with *Terminalia sericea* and *Philenoptera nelsii* in sandveld communities [6,7].

NG32 concession, Okavango Delta

The NG32 concession, a 1000km^2 site in the southeastern section of the Okavango Delta, borders the Moremi Game Reserve, and includes the private concession of Sanctuary Retreats and adjoining Wildlife Management Areas (Fig. 1). The area is characterized by seasonal flooding and rainfall, with rising floodwaters occurring between April to July, and typically highest in August [8]. Rainfall occurs mainly between December and March, with a cool dry season from May to August and a hot dry season from September to November. Comprised of floodplains with a network of waterways, islands, lagoons, reed banks, and pans [9], the area consists mainly of riparian tree species (*Croton megalobotrys, Hyphaene petersiana, Philenoptera violacea, and Diospyros mespiliformis*), dryland tree species of acacia and mopane woodland, and grasslands [10].

Data Collection

A total of 19 lions (13 females and 6 males) and 14 spotted hyenas (10 females and 4 males) were fitted with GPS-satellite collars (IridiumTrackM, Lotek Wireless Inc., Newmarket, Ontario, Canada) programmed to record GPS fixes every 30 minutes during nocturnal periods (18h00 – 6h00 or 17h00 – 8h00) and every 5 minutes for two hours after sunset (19h00 – 21h00) and before sunrise (4h00 – 6h00). All collars recorded diurnal fixes at 10h00 and 14h00 to aid in locating individuals prior to night follows. Collars were also outfitted with dual-axis accelerometers, which measure acceleration on the *x*-axis (representing forward-backward

movement), and on the *y*-axis (representing the sideways or rotary movement) continuously in 8 second bursts, and averaged over 240 seconds. Activity was measured four times per second simultaneously on each axis as the difference in acceleration between two consecutive measurements and given a relative range between 0 and 255 (activity monitor values [AMVs]), characterizing the mean activity/acceleration. The activity data were downloaded from all retrieved collars, and any activity from more than 7 consecutive tracking days were used for subsequent analysis. Collared animals were located in the field from daily uploads of GPS locations via satellite uplink, and, when necessary, radio-tracked from a vehicle with a handheld 3-element yagi antenna (Lotek), and a custom built vehicular-mounted 5-element antenna with a SRX 600 telemetry receiver. Lions and spotted hyenas were monitored on a daily basis via satellite to ensure no mortality events occurred. Individuals whose locations were accessible by the road network prior to sundown were selected for night follows for the maintenance of demographic records and observations of social dynamics. All collared individuals were monitored each day from daily satellite uploads of relocations and up to two or three individuals were selected each day according to their proximity to accessible roads for continuous follows during the night for the ground-truthing of acceleration data and to record any predated events or competitive interactions that occurred.

In the Etosha National Park, 17 collars were deployed on 9 lions and 8 spotted hyenas in the late dry season of 2013 (S1 Fig., Supporting information). Two collars (1 lion and 1 hyena) suffered structural failures during the dry season and were retrieved. A collar was also recovered from a lioness that had been shot outside the park. Subsequently, an additional two lions and one hyena were collared in the wet season of 2014. During the wet season, five additional collars (4 lions and 1 hyena) suffered structural failures and four lion collars were recovered. All but two

hyena collars were retrieved at the end of the study in Etosha, including a collar from a hyena killed by a lion, and a collar from a hyena that had dispersed 120 km outside of the park.

In Botswana, 13 collars were deployed on 6 lions and 5 spotted hyenas along the Chobe/Linyanti (hereafter "Chobe") riverfront in the dry season of 2015, and on 2 lions in the NG32 concession of the Okavango Delta in the late dry/early wet season of 2015 (S1 Fig., Supporting information). All hyena collars included remote drop-off features, eliminating the need to immobilize hyenas for collar retrieval. The drop-off units were programmed to release in the dry season of 2016, but failed to release. Two collars were recovered via wildlife authorities from a hyena shot by a local farmer, and a lioness that had been lured across the river and poached by a Namibian wildlife official. At the end of the study, four collars were recovered from the remaining eleven collars whose batteries had depleted. Collars unable to be removed would eventually fall off due to the deterioration of the collar material.

Prior to collaring, we determined the individuals of lion prides and hyena clans within the study areas over a period of 8 months in Etosha and 5 months in Chobe. We identified lion individuals using whisker/scarring patterns, and used spot patterns/pelage colouration for hyenas. We placed a collar on each of the known groups within the study areas, and data therefore represent the movements and behaviour of the species groups within these areas.

Capture and Sample Collection

All immobilizations were undertaken by registered wildlife veterinarians or under supervision of a veterinarian by persons who successfully passed the Zimbabwe wildlife capture and handling course. All animals were darted with a cartridge-fired projector system (Pneu-Dart, Inc.) from a range of 10-50m. Most of the animals were darted at night using red-filtered

spotlights from vehicles for deployment, with 2 lions darted from a helicopter during the late afternoon for retrievals. In the case of a collared spotted hyena that had dispersed outside of Etosha onto privately owned ranchland, this individual was tracked and darted on foot with permission from the landowner. Initial drug combinations consisted of 3.81 ± 2.17 mg/kg Ketamine hydrochloride, 2.55 ± 1.20 mg/kg Xylazine, 1.48 ± 0.51 mg/kg Tiletamine/Zolazepam hydrochloride (Zoletil), and 0.07 ± 0.02 mg/kg Medetomidine reversed with 0.20 ± 0.07 mg/kg Atipamazole and 0.43 ± 0.19 mg/kg Yohimbine. Later drug combinations consisted of 0.31 ± 0.11 mg/kg Butorphanol with 0.05 ± 0.01 mg/kg Medetomidine and 0.15 ± 0.03 mg/kg Midazolam, reversed with 0.58 ± 0.19 mg/kg Naltrexone and 0.27 ± 0.05 mg/kg Atipamazole, or 1.12 ± 0.65 mg/kg Zoletil and 0.07 ± 0.04 mg/kg Medetomidine, with 1.05 ± 0.87 mg/kg Ketamine as required, reversed with 0.24 ± 0.20 mg/kg Atipamazole and 0.22 ± 0.13 mg/kg Yohimbine.

Recumbency in animals occurred 9 ± 4 mins after darting and immobilizations lasted for 80 ± 65 mins. Eyes were covered with a blindfold to reduce stress and stimuli during handling. Specimens were collected from all animals and included blood, tissue, and feces, with anal gland excretions collected from spotted hyenas only and stored in vials with ethanol. External parasites were noted and visually estimated, with a subset extracted and placed into vials filled with ethanol or methylated spirits. Morphometric measurements were undertaken on all animals after de Waal et al. [11], and included the length of the mane of male lions at four points (B. Stapelkamp, pers. comm). Morphometric measures were used to assign a body condition score to each individual (for lions, Dikeman, unpub.; Treiber & Mann, unpub.; for spotted hyenas, Watts & Holekamp, 2008). All animals collared were fully mature, and age estimates of lions from Etosha were compared against a database of branded lions of known ages. Lion ages were

estimated from visual scoring of body size and pelage colouration, facial scarring, mane development, nose darkness, and based on teeth wear after Smuts et al. [13]. Spotted hyena ages were estimated from visual scoring of body size and pelage colouration, facial scarring, and based on teeth wear after van Horn et al. [14].

Collar Accuracy

We tested collar accuracy by placing all collars out at known GPS locations under a variety of canopy cover ranging from low cover (grassland/shrubs) to very dense cover (heavily vegetated wooded areas) at different times. The location fixes collected by the collars (n = 5159) was used to measure the distances between each consecutive fix. This information was then averaged to give a fix accuracy error rate of 3.49 ± 3.80m, and is representative of the accuracy of collars deployed on the animals.

Etosha Carcass Data

As carcasses provide lions and spotted hyenas with an essential resource, we assessed how distances to available carcasses in the Etosha National Park influenced lion and spotted hyena recursion rates and the duration of stays throughout their ranges. Carcasses were found while following collared individuals or during cluster investigations and were recorded with the ENP's Mortality database (n=30). The relative homogeneous and open plains of Etosha lends itself to the detection of carcasses, vultures circling above carrion, or to conspicuous groups of feeding vultures [15]. Carcasses sighted during road transects and management or (other) research activities were recorded by park personnel and researchers. Carcasses are identified to species, noted as either adults or juveniles, and sexed when possible. Cause of death was

investigated to determine whether a predation event or anthrax death had occurred (other proximate causes of death in the Etosha plain's ungulates are negligible, Gasaway et al. 1996). If a carcass was determined to be predated, the suspected predator was identified or confirmed when possible due to tracks, spoor and field signs. The remains were scored as whether to be fresh, partially consumed or completely consumed leaving only skins, bones and horns. Evidence of anthrax death typically includes bloated, unopened carcasses with bloody discharges at body orifices. Carcasses of suspected anthrax incidences were swabbed with standard cotton swabs, which were submitted to the Central Veterinary Laboratory (Windhoek, Namibia) for confirmation of the presence of anthrax spores. GPS locations were obtained at carcass sites or coordinates were later assigned to specified locations.

ENP's mortality records for the period of the study in Etosha (September 2013 – March 2015, n=304) was used to include carcasses as an available resource on the landscape. Records of carnivore mortalities (n=49) were removed, as they were expected not to pose as an attractant to predators as an available food resource. Animals that were shot to be used for feeding captured carnivores awaiting transport to Cuba were removed, as were old and desiccated carcasses as these were assumed to have no effects on predator movement. The remaining carcass locations were assigned a starting and ending time based on the weight of the species, the estimated date of death and according to the detection and consumption times for carrion [17] or the mean persistence times for predated carcasses [18]. We then measured the minimum Euclidean distances of the locations of collared individuals to these carcass locations only during those times that the carcasses were deemed to have existed on the landscape for.

While we acknowledge that our carcass data do not fully include all the available carcasses that occurred during the study period, and likely excludes many smaller sized carcasses

which are likely to have been completely consumed prior to detection by observers, we assume the survey area to be a random (i.e. unbiased) sample of the larger study area, and the distance of carcasses to the nearest road is assumed to be uniform and an unbiased sample of live animal locations [15]. Although the collective effort in detecting carcasses by park personnel and researchers alike while undertaking other work results in a highly variable surveillance effort, communication amongst researchers ensures that carcasses were reported only once, which safeguards against double sampling. As anthrax carcass sites from previous years pose an attractant to ungulates in subsequent years as a result of the enriched nutrients found in the soil [19], these sites now possess an inherent risk of infection for foraging ungulates as anthrax spores were found to persist in these locations one and two years following initial deposition of the carcass [20,21]. Anthrax carcass records from a recent period of an anthrax outbreak (2009-2010) was used to create a GIS layer of anthrax risk sites, which we defined as the probability of encountering site-attracted foraging ungulates [20,22]. We included carcasses that were identified as anthrax positive from blood swabs that were tested for *B. anthracis* through culture with PCR confirmation [23]. The area representing high incidences of anthrax carcasses (3405.7 km^2) was constructed using the 95% kernel density estimator from the locations of confirmed anthrax positive carcasses. We converted this into a volume UD to obtain probability of presence values for each cell. We subtracted volume UD values from 100 as described above for the combined UD layers.

Statistical Analysis

All statistical analyses were conducted in R version 3.5.1 (R Core Team, 2018), and all GIS applications were conducted in ArcGIS (ESRI ArcMap v.10.0, Redlands, CA, USA). The *t*-

test was used to examine for differences among lion and spotted hyena movement metrics, as well as for differences in the sizes of home ranges and core use areas according to lion and hyena ages, body condition scores, and group sizes. We examined with the *t*-test whether lions and hyenas moved faster and in straighter paths when at closer distances to competitors compared to conspecifics. In addition to the *t*-test, we used a mixed model ANOVA to compare the speed and tortuosity of lions and spotted hyenas between reserves, and to determine whether lion and hyena activity and movement metrics differed according to the lunar cycle (i.e. new moon versus full moon), and when they were within competitor core areas compared to when they were outside of competitor core areas.

References

1. Huntley BJ, Walker BH. Ecology of Tropical Savannas. Berlin, Heidelberg: Springer Berlin Heidelberg; 1982. Available: http://public.eblib.com/choice/publicfullrecord.aspx?p=3091432

2. Auer C. Chemical quality of water at waterholes in the Etosha National Park. Madoqua. 1997;20: 121–128.

3. Engert S. Spatial variability and temporal periodicity of rainfall in the Etosha National Park and surrounding areas in northern Namibia. Madoqua. 1997;20: 115–120.

4. le Roux CJG, Grunow JO, Bredenkamp GJ, Morris JW, Scheepers JC. A classification of the vegetation of the Etosha National Park. South African Journal of Botany. 1988;54: 1–10. doi:10.1016/S0254-6299(16)31355-2

5. Fox J, Vandewalle M, Alexander K. Land Cover Change in Northern Botswana: The Influence of Climate, Fire, and Elephants on Semi-Arid Savanna Woodlands. Land. 2017;73: 1–23. doi:10.3390/land6040073

6. Murray-Hudson M, Combs F, Wolski P, Brown MT. A vegetation-based hierarchical classification for seasonally pulsed floodplains in the Okavango Delta, Botswana. African Journal of Aquatic Science. 2011;36: 223–234. doi:10.2989/16085914.2011.636904

7. Sianga K, Fynn R. The vegetation and wildlife habitats of the Savuti-Mababe-Linyanti ecosystem, northern Botswana. Koedoe. 2017;59: a1406. doi:10.4102/koedoe.v59i2.1406

8. Bennitt E, Hubel TY, Bartlam-Brooks HLA, Wilson AM. Possible causes of divergent population trends in sympatric African herbivores. PLOS ONE. 2019;14: e0213720. doi:10.1371/journal.pone.0213720

9. Beehner JC, Bergman TJ, Cheney DL, Seyfarth RM, Whitten PL. The effect of new alpha males on female stress in free-ranging baboons. Animal Behaviour. 2005;69: 1211–1221. doi:10.1016/j.anbehav.2004.08.014

10. Tsheboeng G, Murray-Hudson M, Kashe K. Regeneration status of riparian tree species in two sites that differ in land-use in the Okavango Delta, Botswana. Journal of Forestry Research. 2017;28: 1073–1082. doi:10.1007/s11676-017-0382-y

11. de Waal HO, Combrinck WJ, Borstlap DG. A comprehensive procedure to measure the body dimensions of large African predators with comments on the repeatability of measurements taken from an immobilized African lion (*Panthera leo*). Journal of Zoology. 2004;262: 393–398. doi:10.1017/S095283690300476X

12. Watts HE, Holekamp KE. Interspecific competition influences reproduction in spotted hyenas. Journal of Zoology. 2008;276: 402–410. doi:10.1111/j.1469-7998.2008.00506.x

13. Smuts GL, Anderson JL, Austin JC. Age determination of the African lion (*Panthera leo*). Journal of Zoology. 1978;185: 115–146. doi:10.1111/j.1469-7998.1978.tb03317.x

14. Van Horn RC, McElhinny TL, Holekamp KE. Age Estimation and Dispersal in the Spotted Hyena (*Crocuta crocuta*). Journal of Mammalogy. 2003;84: 1019–1030. doi:10.1644/BBa-023

15. Bellan SE, Gimenez O, Choquet R, Getz WM. A hierarchical distance sampling approach to estimating mortality rates from opportunistic carcass surveillance data. Methods Ecol Evol. 2013;4: 361–369. doi:10.1111/2041-210x.12021

16. Gasaway WC, Gasaway KT, Berry HH. Persistent low densities of plains ungulates in Etosha National Park, Namibia: testing the food-regulating hypothesis. Can J Zool. 1996;74: 1556–1572. doi:10.1139/z96-170

17. Moleón M, Sánchez-Zapata JA, Sebastián-González E, Owen-Smith N. Carcass size shapes the structure and functioning of an African scavenging assemblage. Oikos. 2015;124: 1391–1403. doi:10.1111/oik.02222

18. Benbow EM, Tomberlin JK, Tarone AM. Carrion Ecology, Evolution, and Their Applications. Boca Raton, London, New York: CRC Press Tayloy & Francis Group; 2016.

19. Turner WC, Imologhome P, Havarua Z, Kaaya GP, Mfune JKE, Mpofu IDT, et al. Soil ingestion, nutrition and the seasonality of anthrax in herbivores of Etosha National Park. Ecosphere. 2013;4: 13. doi:10.1890/ES12-00245.1

20. Turner WC, Kausrud KL, Krishnappa YS, Cromsigt PGM, Ganz HH, Mapaure I, et al. Fatal attraction: vegetation responses to nutrient inputs attract herbivores to infectious anthrax carcass sites. Proceedings of the Royal Society B: Biological Sciences. 2014;281: 1–9.

21. Dougherty ER, Seidel DP, Blackburn JK, Turner WC, Getz WM. A framework for integrating inferred movement behavior into disease risk models. IN REVIEW. 2020; 29.

22. Turner WC, Kausrud KL, Beyer W, Easterday WR, Barandongo ZR, Blaschke E, et al. Lethal exposure: An integrated approach to pathogen transmission via environmental reservoirs. Scientific Reports. 2016;6: 1–13. doi:10.1038/srep27311

23. Beyer W, Bellan S, Eberle G, Ganz HH, Getz WM, Haumacher R, et al. Distribution and Molecular Evolution of *Bacillus anthracis* Genotypes in Namibia. PLoS Neglected Tropical Diseases. 2012;6: e1534. doi:10.1371/journal.pntd.0001534
