# Supplementary material for: Coursing hyenas and stalking lions: The potential for inter- and intraspecific interactions
Source: PLoS One. 2023 Feb 3;18(2):e0265054. doi: 10.1371/journal.pone.0265054 (PMC9897591; doi:10.1371/journal.pone.0265054)
Supplement: S2 Appendix — (PDF) [file pone.0265054.s002.pdf]

S2 Appendix. Detailed analyses of lion and spotted hyena movement descriptors.

Species movement patterns

Investigations into the movement parameters of the predators reveal that, although hyenas are temporally aligned in their activity periods with lions, they exhibited nearly twice the activity rates of lions (mean ± SE: lion 21.88 ± 10.3 AMVs, hyena 40.20 ± 24.0 AMVs, $t = -2.90$, df = 7.2, $p < 0.05$; Fig 5 in main text; S7 Table), moved at characteristically higher speeds than lions in both ecosystems (Etosha mean ± SE: lion 0.187 ± 0.08 m/s, hyena 0.364 ± 0.14 m/s, $t = -5.77$, df = 8.2, $p < 0.001$; Chobe mean ± SE: lion 0.128 ± 0.08 m/s, hyena 0.310 ± 0.16 m/s, $t = -7.73$, df = 6.8, $p < 0.001$; S4 Fig; S7 Table), and had greater nocturnal mean step lengths (mean ± SE: lion 288.90 ± 99.84 m, hyena 618.12 ± 189.85 m, $t = -7.99$, df = 17.5, $p < 0.0001$; S5 Fig; S7 Table).

In both ecosystems, spotted hyenas demonstrated overall larger mean step lengths than lions throughout the 4 hour periods of the 24 hour cycle (Etosha mean ± SE: lion 1256.78 ± 485.44 m, hyena 2008.87 ± 762.16 m; Botswana mean ± SE: lion 897.80 ± 409.67 m, hyena 1744.06 ± 830.56 m), and had consistently larger mean step lengths than lions for each 30 min period throughout the night (Etosha mean ± SE: lion 307.89 ± 139.20 m, hyena 649.43 ± 242.32 m; Botswana mean ± SE: lion 208.08 ± 120.14 m, hyena: 528.75 ± 254.92 m), as well as 5 min dusk/dawn periods (Etosha mean ± SE: lion 54.94 ± 29.85 m, hyena 130.17 ± 52.46 m; Botswana mean ± SE: lion 44.04 ± 27.56 m, hyena 119.03 ± 68.40 m) with some individuals by an order of magnitude (all *t*-tests, $p < 0.05$ for 24 hour periods, and $p < 0.001$ for nocturnal and dusk/dawn periods; S6-S7 Figs; S7 Table). Although there were more lion individuals (61.5%)

with a larger mean step length during dawn periods ($\varkappa^2$ = 5.33, df = 1, p < 0.05), lions travelled significantly further during dusk periods (mean ± SE: at dusk 64.79 ± 2.29 m, at dawn 50.64 ± 3.87 m; t = 3.14, df = 9.7, p < 0.05; S8 Fig). Contrarily, hyena individuals were equally split in whether they had larger mean step length during dusk or dawn periods, and whether they had either small or large differences between the two periods.

In addition, spotted hyenas had consistently significantly larger net-squared displacements (NSD) than lions during nocturnal periods, and across all seasons in Etosha (dry season mean ± SE: lion 12.39 ± 0.26 km^2, hyena 27.40 ± 0.32 km^2, t = -2.97, df = 7.8, p < 0.05; wet season mean ± SE: lion 12.84 ± 0.20 km^2, hyena 32.69 ± 0.44 km^2, t = -2.52, df = 6.6, p < 0.05; S7 Table) and for the dry season in Chobe (mean ± SE: lion 5.21 ± 0.25 km^2, hyena 12.65 ± 0.31 km^2, t = -3.85, df = 6, p < 0.01; S9 Fig; S7 Table). NSDs were generally larger during the wet seasons for both species, although these were not statistically significant. Inspections of the movement parameters over the diel cycle further highlights the similarities between species. Both lions and hyenas typically had larger NSDs during nocturnal than diurnal periods in both ecosystems, although not statistically significant for Chobe spotted hyenas (Etosha mean ± SE: lion, nocturnal 8.25 ± 6.05 km^2, diurnal 2.56 ± 2.16 km^2, t = -6.29, df = 13, p < 0.0001; hyena, nocturnal 17.90 ± 15.24 km^2, diurnal 5.17 ± 7.67 km^2, t = -2.71, df = 10, p < 0.05; Botswana mean ± SE: lion, nocturnal 2.94 ± 3.23 km^2, diurnal 1.13 ± 1.36 km^2, t = -2.77, df = 8, p < 0.05; hyena, nocturnal 6.17 ± 8.90 km^2, diurnal 0.97 ± 1.67 km^2, p > 0.05; S10 Fig; S7 Table).

Spotted hyenas demonstrated relatively more directional movements in the semi-arid Etosha ecosystem, and exhibited increased tortuosity in the wetland ecosystem of Chobe during the 24 hour periods for both seasons (dry season mean ± SE: Etosha -2.012 ± 0.89, Chobe 2.660 ± 1.01, Watson's test statistic: 0.217, p < 0.05; wet season mean ± SE: Etosha 1.637 ± 0.94,

Chobe -2.942 ± 0.90, Watson's test statistic: 0.200, $p < 0.05$; S11 Fig; S7 Table). In addition, lions were significantly more directional than spotted hyenas over the 24 hour period in Etosha (mean ± SE: lion 0.080 ± 0.62, hyena -2.852 ± 0.82, Watson's test statistic: 0.305, $p < 0.01$), and during the wet season in Chobe (mean ± SE: lion 0.209 ± 0.83, hyena -2.942 ± 0.90, Watson's test statistic: 0.189, $p < 0.05$; S11 Fig; S7 Table).

However during the nocturnal period, Etosha lions were relatively more directional than Botswana lions, with the Watson's test significant for the dry season only (mean ± SE: Etosha 0.018 ± 0.85, Botswana -0.068 ± 0.86, Watson's test statistic: 0.177, $0.05 < p < 0.10$; S12 Fig; S7 Table). Contrarily for hyenas, they were more directional in Chobe and had increased tortuosity in Etosha during the dusk/dawn periods, with the Watson's test significant for the wet season only (mean ± SE: Chobe 0.004 ± 0.56, Etosha 0.012 ± 0.58, Watson's test statistic: 0.171, $0.05 < p < 0.10$; S12 Fig; S7 Table). Furthermore, lions were relatively more tortuous than hyenas during dusk and dawn periods (two hours after sunset and two hours before sunrise), although the Watson's test statistic indicated significance only in Etosha (mean ± SE: lion 0.053 ± 0.73, hyena 0.013 ± 0.56, Watson's test statistic: 0.305, $p < 0.01$; S12 Fig; S7 Table).

A fine-scale scrutiny of seasonal path tortuosity over the 24 hour period from both ecosystems reveals lions consistently undertake more tortuous movements than spotted hyenas throughout nocturnal periods, specifically between 18h00-6h00. Although the Watson's Two-Sample Test of Homogeneity only showed significance for some of the time intervals (indicated with asterisks in S13 Fig; Watson's test statistic for Etosha 19h00-20h00, 0.233; 20h00-21h00, 0.250; 21h00-22h00, 0.353; 2h00-3h00, 0.211; 5h00-6h00, 0.211; and Botswana 18h00-19h00, 0.269; 22h00-23h00, 0.215; all p-values < 0.05). Thus, lions were predominantly more tortuous than spotted hyenas in the early phase of the night (18h00-23h00) and in the early morning

(1h00-3h00, and 5h00-6h00), while Chobe spotted hyenas had significantly more tortuous movements than lions during the late morning (6h00-10h00, Watson's test statistic 0.269, $p < 0.05$).

Moreover, lion and hyena movements were found to differ according to the lunar cycle. During nocturnal periods, Etosha lions travelled further during low light conditions (i.e., waxing and waning crescents, $F = 16.36$, $p < 0.0001$; S8 Table), while lions from Botswana travelled further on new moon nights (mean ± SE: new moon 242.81 ± 146.57 m, full moon 222.71 ± 139.56 m, $F = 7.34$, $p < 0.05$; S8 Table) and in the dry season (mean ± SE: new moon 286.42 ± 154.61 m, full moon 223.80 ± 145.39 m, $F = 8.21$, $p < 0.05$; S8 Table). Additionally, Etosha lions had more directional movements during periods of low light conditions (waxing, waning crescents; $F = 34.10$, $p < 0.0001$; S14 Fig), and had more tortuous movements on new moon nights in the dry season (mean ± SE: new moon 0.351 ± 0.86, full moon 0.013 ± 0.84, $F = 22.99$, $p < 0.05$; S8 Table).

However, Chobe lions presented with more tortuous movements during full moon nights (mean ± SE: full moon 0.442 ± 0.91, new moon -0.272 ± 0.80), and in the wet season (mean ± SE: full moon 0.674 ± 0.88, new moon -0.128 ± 0.77), with the Watson's tests significant in both cases (Watson's test statistic: 0.168 and $0.5 < p < 0.10$; S8 Table). Furthermore, during dusk/dawn periods of the wet season, Botswana lions had mostly tortuous movements in first quarter and waning gibbous, with mostly directional movements in waxing gibbous phases ($F = 2.51$, $p < 0.05$; S8 Table).

Conversely, Etosha hyenas had more directional movements during the brightest phases (i.e., waxing/waning gibbous and full moon), and exhibited more tortuous movements during new moon nights, and first and last quarter phases in the wet season during dusk/dawn and

nocturnal periods (F = 2.32, p < 0.05; S8 Table). Similarly, hyenas in Chobe had mostly directional movements during full moon nights (mean ± SE: full moon 0.020 ± 0.96, new moon 0.139 ± 0.94; F = 4.19, p < 0.05; S8 Table).

Species activity patterns

Analyses of lion and spotted hyena activity in relation to the lunar cycle reveals both a seasonal effect and regional differences on the temporal activity patterns during the nocturnal period. Lions and hyenas generally exhibited significantly higher proportion of activity during the periods of the night from dusk to dawn in both ecosystems (Night mean ± SE: lion 0.531 ± 0.014 AMVs, hyena 0.591 ± 0.009 AMVs; Dusk/dawn mean ± SE: lion 0.356 ± 0.010 AMVs, hyena 0.350 ± 0.007 AMVs; t = -16.25, df = 23.1, p < 0.0001; Fig 6 in main text), regardless of moon phase. However, lions in Botswana had increased activity during new moon periods, and also in the dry season (F = 19.39 and 20.30 respectively, p < 0.05; Fig 6 in main text; S8 Table). In addition, the nocturnal activity of both lions and spotted hyenas in Etosha differed according to the various moon phases in the dry season (lion, F = 11.33, p < 0.0001; hyena, F = 20.13, p < 0.0001; S8 Table).

During both new and full moon nights in Etosha, spotted hyenas exhibit heightened proportions of activity in the initial phase of the night (dusk-night mean ± SE: lion 0.159 ± 0.008 AMVs, hyena 0.193 ± 0.009 AMVs, t = -2.77, df = 14, p < 0.05; Fig 6 in main text; S9 Table) However, the opposite is true for Chobe with lions having heightened proportions of activity in the initial phase (dusk-night mean ± SE: lion 0.177 ± 0.013 AMVs, hyena 0.129 ± 0.012 AMVs, t = -2.81, df = 13.9, p < 0.05; Fig 6 in main text; S9 Table), and hyenas during the latter phase (night end-dawn mean ± SE: lion 0.177 ± 0.010 AMVs, hyena 0.241 ± 0.008 AMVs, t = -5.03, df

= 13.7, $p < 0.001$; Fig 6 in main text; S9 Table). Despite the differences in whether lions or hyenas were exhibiting heightened proportions of activity during specific time periods, there remains a temporal shift in activity between lions and hyenas in which periods of heightened proportions of activity is dominated by one species during different time periods of the night. Thus, these distinctive differences in the activity patterns between the two species during the night suggest a temporal partitioning strategy in areas where both species co-exist.

Species variations

Other factors such as age, sex, body condition scores, and group size, were analyzed to determine their effects, if any, on the spatial use patterns of lions and spotted hyenas across both ecosystems. For both species, the size of the home ranges and core areas did not differ significantly with age, despite older animals being weakly correlated with larger home ranges and core areas for lions and smaller home ranges and core areas for hyenas (Spearman's correlation lion home range: 0.46, and core areas: 0.37; spotted hyena home range: -0.08, and core areas: -0.08). However, the relationship between age and the sizes of home ranges and core areas was not significant (lion home range adjusted R^2: 0.01, lion core areas adjusted R^2: 0.12; hyena home range adjusted R^2: -0.05, hyena core areas adjusted R^2: -0.08; all p-values > 0.05). Female hyenas had larger home ranges and core areas in the wet season than male hyenas (mean ± SE home ranges: females 394.16 ± 111.82, males 118.70 ± 12.50, t = 2.45, df = 9.2, $p < 0.05$; core areas: females 101.76 ± 28.00, males 34.45 ± 8.65, t = 2.30, df = 10.0, $p < 0.05$), while there were no differences in the home ranges and core areas between male and female lions.

In addition, higher body condition scores appeared to be weakly correlated with smaller home ranges (Spearman's correlation: -0.14 for each of lions and hyenas), although the

relationship was again insignificant (lion adjusted R^2: -0.06, hyena adjusted R^2: -0.07, all p-values > 0.05). However, there was a significant two-way interaction between sex and body condition scores on the traveling speed and path tortuosity of lions. Although lions of lower body condition scores tended to move at faster speeds during nocturnal periods (F = 42.36, p < 0.05; S10 Table), male lions of low to medium body condition scores travelled at higher speeds than females of similar body condition scores, whereas females with high body condition scores moved at faster speeds than male lions with high body condition scores (F = 81.89, p < 0.05; S10 Table). Despite female lions having more tortuous movements than male lions during dusk/dawn periods (mean ± SE: females 0.117 ± 0.67, males -0.104 ± 0.76; F = 729.79, p < 0.05), the sex-specific tortuosity of lions differed across the categories of low, medium, and high body condition scores (F = 50.06, p < 0.05; S10 Table). Male lions of low body condition had mostly directional movements which increased to more tortuous movements with increasing body condition. Alternatively, female lions of high body condition had mostly directional movements, but had consistently more tortuous movements than males (F = 51.38, p < 0.05; S10 Table). Moreover, spotted hyenas with lower body condition scores also presented with more tortuous movements during nocturnal periods than hyenas of higher body condition scores which had more directional movements (F = 29.46, p < 0.05; S10 Table). In addition, we found no significant effects of group size on the space use patterns of lions or spotted hyenas. Although lions of larger pride sizes were weakly correlated with smaller home ranges (Spearman's correlation: -0.11), this was not a significant relationship (adjusted R^2: -0.05, p > 0.05). Conversely, while spotted hyenas from larger clans were weakly correlated with larger core areas (Spearman's correlation: 0.19), the relationship was not significant (adjusted R^2: -0.09, p > 0.05).

Additional investigations revealed no significant differences in the travelling speed of Etosha lions and spotted hyenas among the various bins of site-attracted foraging ungulate probabilities. However, the tortuosity of Etosha spotted hyenas during dusk/dawn periods differed significantly among the five bins of site-attracted foraging ungulate probabilities. Hyenas exhibited the most tortuous movements within areas of higher probabilities of site-attracted foraging ungulates (mean ± SE: -0.043 ± 0.66), and were relatively more directional in their movements within the regions of lowest probabilities (mean ± SE: 0.015 ± 0.60; $F = 3.83$, $p < 0.05$; S11 Table).
