# Supplementary material for: Coursing hyenas and stalking lions: The potential for inter- and intraspecific interactions
Source: PLoS One. 2023 Feb 3;18(2):e0265054. doi: 10.1371/journal.pone.0265054 (PMC9897591; doi:10.1371/journal.pone.0265054)
Supplement: S1 Table — Relocations were recorded from lions and spotted hyenas in the Etosha National Park, Namibia, the Chobe National Park and Linyanti Conservancy, Botswana, and only from lions in the NG32 concession of the Okavango Delta, Botswana. (PDF) [file pone.0265054.s003.pdf]

S1 Table. Percent frequency of lion and spotted hyena relocations by land cover type. Relocations were recorded from lions and spotted hyenas in the Etosha National Park, Namibia, the Chobe National Park and Linyanti Conservancy, Botswana, and only from lions in the NG32 concession of the Okavango Delta, Botswana.

Region	Land cover type	Percent frequency	
		Lion	Spotted hyena
Etosha	Forest	-	1.68
	Woodland	17.20	17.62
	Shrubland	16.02	26.88
	Grassland	58.75	48.18
	Pan	5.58	3.74
	Cropland	0.02	0.10
	Settlement	0.05	0.01
	Other	2.38	1.79
Botswana	Forest	11.75	28.12
	Woodland	0.16	-
	Shrubland	43.21	59.15
	Grassland	27.52	4.81
	Pan	-	0.04
	Wetland	13.10	6.55
	Cropland	-	0.03
	Settlement	0.01	-
	Other	4.25	1.29
