# Supplementary material for: Coursing hyenas and stalking lions: The potential for inter- and intraspecific interactions
Source: PLoS One. 2023 Feb 3;18(2):e0265054. doi: 10.1371/journal.pone.0265054 (PMC9897591; doi:10.1371/journal.pone.0265054)
Supplement: S2 Table — Kernel density (a, c) and a-LoCoH (b, d) area measures for home ranges and core use areas (km2) of lion (a, b) and spotted hyena (c, d) individuals. Upper panels consists of lion and hyena individuals from the Etosha National Park, Namibia, with lower panels from the Chobe National Park and Linyanti Conservancy. For lions, the NG32 concession in the Okavango Delta, Botswana in also included. CS = combined seasons, DS = dry season, WS = wet season. (PDF) [file pone.0265054.s004.pdf]

S2 Table. Seasonal utilization distributions (UDs) for all collared lion and spotted hyena individuals. Kernel density (a, c) and *a*-LoCoH (b, d) area measures for home ranges and core use areas (km^2) of lion (a, b) and spotted hyena (c, d) individuals. Upper panels consist of lion and hyena individuals from the Etosha National Park, Namibia, with lower panels from the Chobe National Park and Linyanti Conservancy. For lions, the NG32 concession in the Okavango Delta, Botswana is also included. CS = combined seasons, DS = dry season, WS = wet season.

(a)

	HOME RANGE (95%)									CORE AREA (50%)								
						DRY SEASON		WET SEASON							DRY SEASON		WET SEASON	
**INDIVIDUAL**	**CS**	**DS**	**WS**	**DAY**	**NIGHT**	**DAY**	**NIGHT**	**DAY**	**NIGHT**	**CS**	**DS**	**WS**	**DAY**	**NIGHT**	**DAY**	**NIGHT**	**DAY**	**NIGHT**
**OK-33863**	456	284	457	604	586	326	308	604	586	129	62	128	170	168	73	69	170	168
**RE-33864**	538	117	539	691	674	120	119	691	674	106	23.7	107	137	134	24.8	27.1	137	134
**NU-33865**	841	614	653	1067	1051	837	825	842	834	198	172	158	252	245	230	227	215	208
**MO-33866**	1192	1111	871	1440	1433	1497	1487	1130	1128	277	188	242	370	367	296	290	307	308
**OJ-33867**	787	415	765	937	979	443	474	937	979	168	87	160	224	221	97	103	224	224
**SU-33868**	667	495	646	915	872	810	726	897	868	112	74	114	159	147	159	141	162	149
**OM-34308**	369	131	375	487	490	131	144	487	490	87	24.2	90	116	117	25.1	28.2	116	117
**LU-34308**	214	185	129	241	254	212	220	195	191	29.3	27.5	33.5	36.3	34.6	34.5	32.4	49.7	48.6
**OF-34309**	375	436	361	418	424	433	472	418	424	87	56	87	125	119	62	67	125	119
**G2-35678**	329	329	127	439	424	439	424	-	-	62	62	27.7	83	83	83	83	-	-
Mean	***577***	***411***	***435***	***724***	***719***	***525***	***520***	***688***	***686***	***125***	***135***	***115***	***167***	***163***	***109***	***107***	***167***	***164***
SD	***297***	***294***	***276***	***364***	***361***	***422***	***411***	***293***	***296***	***72***	***189***	***63***	***95***	***94***	***92***	***88***	***74***	***75***
**SW-33950**	255	208	272	314	331	278	290	341	356	56	45.5	63	69	72	62	64	79	82
**AF-34308**	210	40.0	236	284	297	61	58	326	341	36.8	10.7	43.8	56	54	17.4	16.1	69	68
**BE-35678**	727	38.5	720	1196	1096	-	-	1196	1099	213	11.4	211	335	316	-	-	335	317
**BO-35947**	296	304	216	399	399	451	461	270	265	67	79	46.6	84	85	120	124	56	56
**AM-36714**	1032	710	1171	1378	1386	901	971	1693	1612	132	71	214	258	226	154	137	381	337
**BA-36715**	47.0	49.8	38.1	48.9	48.1	42.8	40.0	50	50	8.0	7.7	7.5	8.0	8.1	7.5	7.3	8.5	8.8
**KW-36716**	244	125	304	283	304	147	161	388	404	39.5	29.6	59	48.5	53	34.4	37.8	81	88
**KB-36717**	97	72	113	126	137	75	74	164	190	11.3	9.3	14.6	15.6	17.5	11.1	11.3	23.7	28.5
Mean	***363***	***193***	***384***	***504***	***500***	***279***	***294***	***554***	***540***	***70***	***33.0***	***82***	***109***	***104***	***58***	***57***	***129***	***123***
SD	***339***	***229***	***377***	***498***	***477***	***311***	***335***	***576***	***533***	***69***	***29.0***	***83***	***120***	***109***	***58***	***54***	***144***	***129***

(b)

	HOME RANGE (95%)									CORE AREA (50%)								
						DRY SEASON		WET SEASON							DRY SEASON		WET SEASON	
**INDIVIDUAL**	**CS**	**DS**	**WS**	**DAY**	**NIGHT**	**DAY**	**NIGHT**	**DAY**	**NIGHT**	**CS**	**DS**	**WS**	**DAY**	**NIGHT**	**DAY**	**NIGHT**	**DAY**	**NIGHT**
**OK-33863**	295	54	283	209	269	39.8	69	209	269	144	16.1	123	84	134	6.5	17.3	84	134
**RE-33864**	345	41.8	335	309	362	0.2	38.6	309	362	92	12.2	85	96	91	0.1	14.9	96	91
**NU-33865**	580	362	358	553	609	326	371	384	449	174	120	131	177	212	167	185	169	189
**MO-33866**	622	370	294	734	829	446	554	454	507	178	103	77	232	298	99	114	176	208
**OJ-33867**	348	105	273	265	393	41.7	101	265	393	165	33.3	98	92	160	33.8	21.6	92	160
**SU-33868**	484	162	375	537	503	161	228	412	423	101	46.9	99	97	106	23.1	25.1	99	106
**OM-34308**	221	66	233	189	244	29.5	69	189	244	91	14.4	74	68	90	11.4	18.3	68	90
**LU-34308**	170	150	43.8	149	210	129	167	39.0	44.7	31.1	28.6	18.5	33.9	44.2	43.3	33.7	18.6	21.5
**OF-34309**	327	141	254	201	256	15.1	200	201	256	104	16.1	71	91	63	5.5	27.1	91	63
**G2-35678**	229	229	-	220	258	220	258	-	-	55	54.5	-	78	81	78	81	-	-
Mean	***362***	***168***	***272***	***337***	***394***	***141***	***206***	***273***	***328***	***113***	***44.5***	***86***	***105***	***128***	***46.8***	***54***	***99***	***118***
SD	***153***	***118***	***98***	***199***	***200***	***150***	***160***	***131***	***140***	***50***	***38.2***	***33.1***	***57***	***77***	***53***	***57***	***48.2***	***60***
**SW-33950**	171	113	171	163	194	134	106	134	181	43.2	25.9	40.2	61	51	64	32.2	64	73
**AF-34308**	87	16.0	89	117	120	14.3	17.2	117	125	30.5	5.8	27.8	35.0	28.3	5.3	6.6	40.0	25
**BE-35678**	282	1.5	275	241	314	-	-	241	310	59	0.8	57	79	144	-	-	79	140
**BO-35947**	271	230	180	243	316	191	274	166	185	70	73	46.1	82	83	82	99	56	50
**AM-36714**	934	604	849	751	692	161	-	729	651	195	168	182	353	125	43.3	49	418	358
**BA-36715**	52	49	47.4	44.8	43	30.2	29.6	39.0	38.2	9.8	8.8	10.9	11.6	11.5	7.6	6.6	11.0	10.7
**KW-36716**	174	89	167	183	208	79	85	192	225	36.5	21.5	37.5	34.2	44.7	29.5	27.7	42.7	67
**KB-36717**	88	59	83	74	78	42.1	32.1	75	68	11.6	7.1	10.9	19.2	16.5	18.7	11.6	22.6	18.2
Mean	***257***	***145***	***233***	***227***	***246***	***93***	***91***	***212***	***223***	***57***	***38.9***	***52***	***84***	***63***	***35.7***	***33.2***	***92***	***93***
SD	***286***	***199***	***259***	***223***	***206***	***69***	***97***	***219***	***193***	***60***	***57***	***55***	***112***	***49.6***	***28.8***	***32.9***	***134***	***115***

(c)

	HOME RANGE (95%)									CORE AREA (50%)								
						DRY SEASON		WET SEASON							DRY SEASON		WET SEASON	
**INDIVIDUAL**	**CS**	**DS**	**WS**	**DAY**	**NIGHT**	**DAY**	**NIGHT**	**DAY**	**NIGHT**	**CS**	**DS**	**WS**	**DAY**	**NIGHT**	**DAY**	**NIGHT**	**DAY**	**NIGHT**
**GO-33869**	235	185	265	273	283	205	210	315	332	57	42.6	66	62	67	55	50.8	67.6	80.6
**TJ-33870**	290	307	289	326	362	-	-	326	362	42.1	70	42	40.6	60	-	-	40.6	60.4
**NE-33871**	284	215	309	249	315	168	238	292	360	46.1	25.6	60	36.3	63	15.1	35.0	65.5	87.8
**SA-33872**	1355	201	1465	2013	1973	258	251	2117	2056	210	49.0	325	365	358	70	63.8	495.0	495.9
**AU-33873**	2808	136	4128	4126	4124	148	185	6007	6025	383	10.8	869	639	635	13.5	17.8	1357.5	1355.2
**OM-33874**	299	256	311	283	341	245	295	290	362	43.1	36.7	43.7	39.8	52	38.0	46.1	42.3	56.7
**SU-33951**	169	298	168	264	267	-	-	264	267	27.6	69	27.1	39.7	41	-	-	39.7	41.3
**WO-34310**	309	309	-	354	353	354	353	-	-	69	69	-	74	84	74	84	-	-
Mean	***719***	***239***	***991***	***986***	***1002***	***230***	***255***	***1373***	***1395***	***110***	***46.6***	***205***	***162***	***170***	***44.2***	***49.7***	***301***	***311***
SD	***927***	***64***	***1454***	***1405***	***1388***	***75***	***61***	***2153***	***2140***	***125***	***22.1***	***311***	***223***	***215***	***26.3***	***23.0***	***494***	***488***
**AR-33869**	142	-	142	137	162	-	-	137	162	19.6	-	19.6	25.9	31.9	-	-	25.9	31.9
**IH-33870**	136	119	147	108	156	101	144	103	152	20.4	20.1	13.9	20.0	29.4	19.2	29.4	13.5	24.9
**KW-33871**	82	83	60	45.9	95	48.2	99	13.7	60	10.1	10.9	5.4	7.1	14.5	7.5	16.0	2.3	9.2
**RV-33873**	136	96	137	140	149	-	-	140	149	29.1	16.6	29.5	30.4	41.4	-	-	29.8	41.4
**SR-34310**	1898	173	3655	2932	3025	154	211	5553	5599	164	24.8	463	271	287	28.8	38.7	840	866
Mean	***479***	***118***	***828***	***673***	***717***	***101***	***151***	***1189***	***1224***	***48.7***	***18.1***	***106***	***71***	***81***	***18.5***	***28.0***	***182***	***194***
SD	***794***	***39.6***	***1581***	***1264***	***1290***	***53***	***56***	***2440***	***2446***	***65***	***5.9***	***200***	***112***	***116***	***10.7***	***11.4***	***368***	***376***

(d)

INDIVIDUAL	HOME RANGE (95%) CS	DS	WS	DAY	NIGHT	DRY SEASON DAY	DRY SEASON NIGHT	WET SEASON DAY	WET SEASON NIGHT	CORE AREA (50%) CS	DS	WS	DAY	NIGHT	DRY SEASON DAY	DRY SEASON NIGHT	WET SEASON DAY	WET SEASON NIGHT
**GO-33869**	232	175	242	216	258	129	166	213	280	75	51	91	110	83	58	55	88	101
**TJ-33870**	249	9.6	246	132	259	-	-	132	259	44.8	4.6	44.8	19.6	44.3	-	-	19.6	44
**NE-33871**	282	217	282	205	285	125	198	195	284	79	39.2	93	35.1	56	5.2	35.4	63	95
**SA-33872**	786	171	831	714	876	147	168	762	939	118	54	220	123	138	57	73	234	422
**AU-33873**	1057	112	1148	853	1075	29.8	110	969	1239	168	8.4	281	106	224	2.6	12.8	169	293
**OM-33874**	292	225	299	246	309	148	183	230	303	76	55	87	90	90	34.6	77	81	91
**SU-33951**	135	5.7	134	43.0	62	-	-	43.0	62	55	0.4	55	10.7	18.3	-	-	10.7	18.3
**WO-34310**	275	275	-	239	289	239	289	-	-	67	67	-	68	88	68	88	-	-
Mean	***414***	***149***	***455***	***331***	***427***	***136***	***186***	***363***	***481***	***85***	***35.0***	***124***	***70***	***93***	***37.6***	***57***	***95***	***152***
SD	***326***	***99***	***380***	***289***	***352***	***67***	***59***	***354***	***432***	***39.6***	***26.4***	***90***	***43.7***	***64***	***28.3***	***28.5***	***80***	***148***
**AR-33869**	106	-	106	38.7	54	-	-	38.7	54	25.8	-	25.8	14.5	29.1	-	-	14.5	29.1
**IH-33870**	128	114	133	70	117	61	90	61	84	45.8	24.6	11.5	16.8	42.4	15.5	30.6	4.0	34.9
**KW-33871**	79	77	38.7	12.5	53	11.9	52	-	9.0	9.5	9.5	2.7	2.4	19.8	1.5	27.3	-	7.5
**RV-33873**	135	37.8	131	65	80	-	-	65	80	43.1	5.2	43.1	27.0	40.0	-	-	27.0	40.0
**SR-34310**	523	156	587	514	594	84	134	515	1016	67	43.7	133	85	97	34.2	33.2	98	148
Mean	***194***	***96***	***199***	***140***	***179***	***52***	***92***	***170***	***249***	***38.1***	***20.8***	***43.2***	***29.1***	***45.7***	***17.1***	***30.4***	***36.0***	***52***
SD	***185***	***50***	***220***	***210***	***233***	***37***	***41***	***230***	***430***	***21.6***	***17.4***	***52***	***32.4***	***30.3***	***16.4***	***3.0***	***42.6***	***55***
