# Supplementary material for: Coursing hyenas and stalking lions: The potential for inter- and intraspecific interactions
Source: PLoS One. 2023 Feb 3;18(2):e0265054. doi: 10.1371/journal.pone.0265054 (PMC9897591; doi:10.1371/journal.pone.0265054)
Supplement: S3 Table — Total overlapped areas in km2 between lions’ (vertical column) and spotted hyenas’ (horizontal column) home ranges and core areas in the (a) Etosha National Park, Namibia; (b) Chobe National Park and Linyanti Conservancy, Botswana. Utilization distributions were generated with the home range (95%) and core use area (50%) kernel density estimator (i) and a-LoCoH (ii) isopleths. Males are underlined. An asterisk denotes mortality. (PDF) [file pone.0265054.s005.pdf]

S3 Table. Areas of overlap in lion and spotted hyena ranges. Total overlapped areas in km^2 between lions' (vertical column) and spotted hyenas' (horizontal column) home ranges and core areas in the (a) Etosha National Park, Namibia; (b) Chobe National Park and Linyanti Conservancy, Botswana. Utilization distributions were generated with the home range (95%) and core use area (50%) kernel density estimator (i) and *a*-LoCoH (ii) isopleths. Males are underlined. An asterisk denotes mortality.

(a)(i)

	HOME RANGE (95%)															
	DRY SEASON								WET SEASON							
	SPOTTED HYENA															
LION	GO-33869	TJ-33870	NE-33871	SA-33872	AU-33873	OM-33874	SU-33951	WO-34310	GO-33869	TJ-33870*	NE-33871	SA-33872	AU-33873*	OM-33874	SU-33951*	WO-34310
OK-33863				0				210				199				-
RE-33864		85		94	0		64			116		399	67		167	-
NU-33865	181	130		6.1					231	108		0				-
MO-33866	90	301		161			179		53	134		12			2.5	-
OJ-33867			167	0		36.8		93			193	375		31.6		-
SU-33868		122		171			198			195		482			168	-
OM-34308*			0	0	0	102					62	109	47.9	173		-
LU-34308			70	0.5		76		0.4			12	129		50		-
OF-34309*				0	0							68	361			-
G2-35678				0	0	1.7			-	-	-	-	-	-	-	-

	CORE AREA (50%)															
	DRY SEASON								WET SEASON							
	SPOTTED HYENA															
LION	GO-33869	TJ-33870	NE-33871	SA-33872	AU-33873	OM-33874	SU-33951	WO-34310	GO-33869	TJ-33870*	NE-33871	SA-33872	AU-33873*	OM-33874	SU-33951*	WO-34310
OK-33863				0				24.5				0				-
RE-33864		0		5.2	0		0.8			3.0		20.6	0		20.4	-
NU-33865	33.2	12.3		0					19.9	0		0				-
MO-33866	0	65		4.8			3.4		0	16.0		0			0	-
OJ-33867			6.5	0		0		0			0.01	13.2		0		-
SU-33868		0		7.2			11.3			3.0		41.5			18.5	-
OM-34308*			0	0	0	0					0	0	0	10.7		-
LU-34308			0	0		0		0			0	31.6		1.9		-
OF-34309*				0	0							0	51			-
G2-35678				0	0	0			-	-	-	-	-	-	-	-

(a)(ii)

HOME RANGE (95%)

		DRY SEASON								WET SEASON							
		SPOTTED HYENA															
		GO-33869	TJ-33870	NE-33871	SA-33872	AU-33873	OM-33874	SU-33951	WO-34310	GO-33869	TJ-33870*	NE-33871	SA-33872	AU-33873*	OM-33874	SU-33951*	WO-34310
LION	OK-33863				0				48.7				43.8				-
	RE-33864		0.6		38.8	0		0			76		236	0		124	-
	NU-33865	126	4.9		0.1					172	60		0				-
	MO-33866	22.8	6.8		62			0		17	23.2		0			0	-
	OJ-33867			56	0		0		27.1			69	98		4.4		-
	SU-33868		0.7		72			0.9			131		260			124	-
	OM-34308*			0	0	0	57					35.8	0.2	0	100		-
	LU-34308			53	0		58		0			1.3	43.8		25.2		-
	OF-34309*				0	0							0.7	102			-
	G2-35678				0	0	0			-	-	-	-	-	-	-	-

CORE AREA (50%)

		DRY SEASON								WET SEASON							
		SPOTTED HYENA															
		GO-33869	TJ-33870	NE-33871	SA-33872	AU-33873	OM-33874	SU-33951	WO-34310	GO-33869	TJ-33870*	NE-33871	SA-33872	AU-33873*	OM-33874	SU-33951*	WO-34310
LION	OK-33863				0				4.2				0				-
	RE-33864		0		0.2	0		0			0		56	0		26.4	-
	NU-33865	32.3	1.1		0					34.8	0		0				-
	MO-33866	0	3.3		0			0		0	14.7		0			0	-
	OJ-33867			1.3	0		0		0			0	4.6		0		-
	SU-33868		0		21.5			0			1.7		69			40.7	-
	OM-34308*			0	0	0	0.2					0	0	0	16.0		-
	LU-34308			0	0		0.1		0			0	9.7		0.3		-
	OF-34309*				0	0							0	28.8			-
	G2-35678				0	0	0			-	-	-	-	-	-	-	-

(b)(i)

HOME RANGE (95%)

		DRY SEASON					WET SEASON				
		SPOTTED HYENA									
		AR-33869	IH-33870	KW-33871	RV-33873*	SR-34310	AR-33869*	IH-33870	KW-33871*	RV-33873	SR-34310*
LION	SW-33950	-	67			96		104			272
	AF-34308	-			0					78	
	BE-35678*	-			0		93			15.4	
	BO-35947	-									
	AM-36714	-			65		84			131	
	BA-36715	-									
	KW-36716*	-	4.1	40.1		25.0		26.1	47.0		198
	KB-36717*	-	29.8	0.9		47.1		42.6	2.8		106

CORE AREA (50%)

		DRY SEASON					WET SEASON				
		SPOTTED HYENA									
		AR-33869	IH-33870	KW-33871	RV-33873*	SR-34310	AR-33869*	IH-33870	KW-33871*	RV-33873	SR-34310*
LION	SW-33950	-	2.0			2.9		0			56
	AF-34308	-			0					0	
	BE-35678*	-			0		0			0	
	BO-35947	-									
	AM-36714	-			0		0			22.0	
	BA-36715	-									
	KW-36716*	-	0	0		0		0	0		7.8
	KB-36717*	-	0	0		0		0	0		13.7

(b)(ii)

HOME RANGE (95%)

		DRY SEASON					WET SEASON				
		SPOTTED HYENA									
		AR-33869	IH-33870	KW-33871	RV-33873*	SR-34310	AR-33869*	IH-33870	KW-33871*	RV-33873	SR-34310*
LION	SW-33950	-	52			65		75			130
	AF-34308	-			0					23.7	
	BE-35678*	-			0		32.8			0	
	BO-35947	-									
	AM-36714	-			17.7		64			97	
	BA-36715	-									
	KW-36716*	-	3.2	23.7		15.5		10.0	13.1		13.2
	KB-36717*	-	27.7	0		40.7		30.3	0.3		39.1

CORE AREA (50%)

		DRY SEASON					WET SEASON				
		SPOTTED HYENA									
		AR-33869	IH-33870	KW-33871	RV-33873*	SR-34310	AR-33869*	IH-33870	KW-33871*	RV-33873	SR-34310*
LION	SW-33950	-	0.6			4.6		0			0.4
	AF-34308	-			0					0	
	BE-35678*	-			0		0.1			0	
	BO-35947	-									
	AM-36714	-			0		0			0	
	BA-36715	-									
	KW-36716*	-	0	0		0		0	0		0
	KB-36717*	-	0	0		1.5		0	0		0.7
