# Supplementary material for: Coursing hyenas and stalking lions: The potential for inter- and intraspecific interactions
Source: PLoS One. 2023 Feb 3;18(2):e0265054. doi: 10.1371/journal.pone.0265054 (PMC9897591; doi:10.1371/journal.pone.0265054)
Supplement: S4 Table — Total proportion of lion (vertical column) home ranges and core areas overlapped by spotted hyena individuals (horizontal column) in the (a) Etosha National Park, Namibia; (b) Chobe National Park and Linyanti Conservancy, Botswana. Utilization distributions were generated with the home range (95%) and core use area (50%) kernel density estimator (i) and a-LoCoH (ii) isopleths. Males are underlined. An asterisk denotes mortality. (PDF) [file pone.0265054.s006.pdf]

S4 Table. Proportion of overlaps with spotted hyenas in lion ranges. Total proportion of lion (vertical column) home ranges and core areas overlapped by spotted hyena individuals (horizontal column) in the (a) Etosha National Park, Namibia; (b) Chobe National Park and Linyanti Conservancy, Botswana. Utilization distributions were generated with the home range (95%) and core use area (50%) kernel density estimator (i) and *a*-LoCoH (ii) isopleths. Males are underlined. An asterisk denotes mortality.

(a)(i)

HOME RANGE (95%)

		DRY SEASON								WET SEASON							
		SPOTTED HYENA															
		GO-33869	TJ-33870	NE-33871	SA-33872	AU-33873	OM-33874	SU-33951	WO-34310	GO-33869	TJ-33870*	NE-33871	SA-33872	AU-33873*	OM-33874	SU-33951*	WO-34310
LION	OK-33863				0				0.740				0.436				-
	RE-33864		0.730		0.806	0		0.549			0.214		0.741	0.125		0.309	-
	NU-33865	0.294	0.211		0.0099					0.354	0.165		0				-
	MO-33866	0.081	0.271		0.145			0.161		0.061	0.154		0.014			0.0029	-
	OJ-33867			0.402	0		0.089		0.224			0.252	0.490		0.041		-
	SU-33868		0.246		0.345			0.401			0.302		0.746			0.260	-
	OM-34308*			0	0	0	0.776					0.166	0.290	0.128	0.461		-
	LU-34308			0.376	0.0027		0.409		0.002			0.091	1.0		0.389		-
	OF-34309*				0	0							0.187	1.0			-
	G2-35678				0	0	0.005			-	-	-	-	-	-	-	-

CORE AREA (50%)

		DRY SEASON								WET SEASON							
		SPOTTED HYENA															
		GO-33869	TJ-33870	NE-33871	SA-33872	AU-33873	OM-33874	SU-33951	WO-34310	GO-33869	TJ-33870*	NE-33871	SA-33872	AU-33873*	OM-33874	SU-33951*	WO-34310
LION	OK-33863				0				0.395				0				-
	RE-33864		0		0.219	0		0.034			0.028		0.193	0		0.192	-
	NU-33865	0.193	0.071		0					0.126	0		0				-
	MO-33866	0	0.343		0.025			0.018		0	0.066		0			0	-
	OJ-33867			0.073	0		0		0			0	0.082		0		-
	SU-33868		0		0.097			0.152			0.026		0.365			0.163	-
	OM-34308*			0	0	0	0					0	0	0	0.119		-
	LU-34308			0	0		0		0			0	0.943		0.057		-
	OF-34309*				0	0							0	0.594			-
	G2-35678				0	0	0			-	-	-	-	-	-	-	-

(a)(ii)

HOME RANGE (95%)

LION	DRY SEASON – SPOTTED HYENA								WET SEASON – SPOTTED HYENA							
	GO-33869	TJ-33870	NE-33871	SA-33872	AU-33873	OM-33874	SU-33951	WO-34310	GO-33869	TJ-33870*	NE-33871	SA-33872	AU-33873*	OM-33874	SU-33951*	WO-34310
OK-33863				0				0.895				0.155				-
RE-33864		0.014		0.928	0		0			0.225		0.703	0		0.369	-
NU-33865	0.349	0.014		0.00028					0.481	0.167		0				-
MO-33866	0.062	0.018		0.167			0		0.059	0.079		0			0	-
OJ-33867			0.536	0		0		0.257			0.253	0.360		0.016		-
SU-33868		0.0043		0.444			0.0056			0.348		0.695			0.330	-
OM-34308*			0	0	0	0.871					0.154	0.00086	0	0.430		-
LU-34308			0.354	0		0.385		0			0.030	1.0		0.575		-
OF-34309*				0	0							0.0028	0.402			-
G2-35678				0	0	0			-	-	-	-	-	-	-	-

CORE AREA (50%)

LION	DRY SEASON – SPOTTED HYENA								WET SEASON – SPOTTED HYENA							
	GO-33869	TJ-33870	NE-33871	SA-33872	AU-33873	OM-33874	SU-33951	WO-34310	GO-33869	TJ-33870*	NE-33871	SA-33872	AU-33873*	OM-33874	SU-33951*	WO-34310
OK-33863				0				0.261				0				-
RE-33864		0		0.016	0		0			0		0.651	0		0.309	-
NU-33865	0.269	0.0092		0					0.265	0		0				-
MO-33866	0	0.032		0			0		0	0.191		0			0	-
OJ-33867			0.039	0		0		0			0	0.047		0		-
SU-33868		0		0.458			0			0.017		0.699			0.411	-
OM-34308*			0	0	0	0.014					0	0	0	0.217		-
LU-34308			0	0		0.0035		0			0	0.524		0.016		-
OF-34309*				0	0							0	0.403			-
G2-35678				0	0	0			-	-	-	-	-	-	-	-

(b)(i)

		HOME RANGE (95%)									
		DRY SEASON					WET SEASON				
		SPOTTED HYENA									
		AR-33869	IH-33870	KW-33871	RV-33873*	SR-34310	AR-33869*	IH-33870	KW-33871*	RV-33873	SR-34310*
LION	SW-33950	-	0.324			0.462		0.382			1.0
	AF-34308	-			0					0.331	
	BE-35678*	-			0		0.129			0.021	
	BO-35947	-									
	AM-36714	-			0.092		0.072			0.111	
	BA-36715	-									
	KW-36716*	-	0.033	0.320		0.200		0.086	0.155		0.651
	KB-36717*	-	0.413	0.012		0.653		0.378	0.025		0.945

		CORE AREA (50%)									
		DRY SEASON					WET SEASON				
		SPOTTED HYENA									
		AR-33869	IH-33870	KW-33871	RV-33873*	SR-34310	AR-33869*	IH-33870	KW-33871*	RV-33873	SR-34310*
LION	SW-33950	-	0.044			0.064		0			0.895
	AF-34308	-			0					0	
	BE-35678*	-			0		0			0	
	BO-35947	-									
	AM-36714	-			0		0			0.103	
	BA-36715	-									
	KW-36716*	-	0	0		0		0	0		0.133
	KB-36717*	-	0	0		0		0	0		0.938

(b)(ii)

		HOME RANGE (95%)									
		DRY SEASON					WET SEASON				
		SPOTTED HYENA									
		AR-33869	IH-33870	KW-33871	RV-33873*	SR-34310	AR-33869*	IH-33870	KW-33871*	RV-33873	SR-34310*
LION	SW-33950	-	0.466			0.575		0.437			0.763
	AF-34308	-			0					0.266	
	BE-35678*	-			0		0.119			0	
	BO-35947	-									
	AM-36714	-			0.029		0.075			0.114	
	BA-36715	-									
	KW-36716*	-	0.036	0.267		0.174		0.060	0.078		0.079
	KB-36717*	-	0.470	0		0.691		0.364	0.0036		0.470

		CORE AREA (50%)									
		DRY SEASON					WET SEASON				
		SPOTTED HYENA									
		AR-33869	IH-33870	KW-33871	RV-33873*	SR-34310	AR-33869*	IH-33870	KW-33871*	RV-33873	SR-34310*
LION	SW-33950	-	0.023			0.178		0			0.015
	AF-34308	-			0					0	
	BE-35678*	-			0		0.125			0	
	BO-35947	-									
	AM-36714	-			0		0			0	
	BA-36715	-									
	KW-36716*	-	0	0		0		0	0		0
	KB-36717*	-	0	0		0.211		0	0		0.064
