# Supplementary material for: Coursing hyenas and stalking lions: The potential for inter- and intraspecific interactions
Source: PLoS One. 2023 Feb 3;18(2):e0265054. doi: 10.1371/journal.pone.0265054 (PMC9897591; doi:10.1371/journal.pone.0265054)
Supplement: S5 Table — Total proportion of spotted hyena (vertical column) home ranges and core areas overlapped by lion individuals (horizontal column) in the (a) Etosha National Park, Namibia; (b) Chobe National Park and Linyanti Conservancy, Botswana. Utilization distributions were generated with the home range (95%) and core use area (50%) kernel density estimator (i) and a-LoCoH (ii) isopleths. Males are underlined. An asterisk denotes mortality. (PDF) [file pone.0265054.s007.pdf]

S5 Table. Proportion of overlaps with lions in spotted hyena ranges. Total proportion of spotted hyena (vertical column) home ranges and core areas overlapped by lion individuals (horizontal column) in the (a) Etosha National Park, Namibia; (b) Chobe National Park and Linyanti Conservancy, Botswana. Utilization distributions were generated with the home range (95%) and core use area (50%) kernel density estimator (i) and *a*-LoCoH (ii) isopleths. Males are underlined. An asterisk denotes mortality.

(a)(i)

HOME RANGE (95%)

		DRY SEASON										WET SEASON									
		LION																			
		OK	RE	NU	MO	OJ	SU	OM*	LU	OF*	G2	OK	RE	NU	MO	OJ	SU	OM*	LU	OF*	G2
SPOTTED HYENA	GO-33869			0.974	0.487									0.873	0.201						-
	TJ-33870		0.277	0.423	0.983		0.397						0.400	0.372	0.464		0.676				-
	NE-33871					0.776		0	0.324							0.622		0.201	0.038		-
	SA-33872	0	0.466	0.030	0.801	0	0.849	0	0.0025	0	0	0.136	0.273	0	0.008	0.256	0.329	0.074	0.088	0.046	-
	AU-33873		0					0		0	0		0.016					0.012		0.087	-
	OM-33874					0.144		0.397	0.296		0.007					0.102		0.556	0.161		-
	SU-33951		0.215		0.601		0.665						0.993		0.015		1.0				-
	WO-34310	0.678				0.301			0.001			-	-	-	-	-	-	-	-	-	-

CORE AREA (50%)

		DRY SEASON										WET SEASON									
		LION																			
		OK	RE	NU	MO	OJ	SU	OM*	LU	OF*	G2	OK	RE	NU	MO	OJ	SU	OM*	LU	OF*	G2
SPOTTED HYENA	GO-33869			0.779	0									0.302	0						-
	TJ-33870		0	0.175	0.919		0						0.071	0	0.381		0.071				-
	NE-33871					0.254		0	0							0		0	0		-
	SA-33872	0	0.106	0	0.098	0	0.147	0	0	0	0	0	0.063	0	0	0.041	0.128	0	0.097	0	-
	AU-33873		0					0		0	0		0					0		0.059	-
	OM-33874					0		0	0		0					0		0.245	0.043		-
	SU-33951		0.012		0.049		0.164						0.753		0		0.683				-
	WO-34310	0.355				0			0			-	-	-	-	-	-	-	-	-	-

(a)(ii)

HOME RANGE (95%)

	DRY SEASON										WET SEASON									
	LION																			
SPOTTED HYENA	OK	RE	NU	MO	OJ	SU	OM*	LU	OF*	G2	OK	RE	NU	MO	OJ	SU	OM*	LU	OF*	G2
GO-33869			0.723	0.131									0.713	0.071						-
TJ-33870		0.063	0.51	0.708		0.073						0.307	0.243	0.094		0.53				-
NE-33871					0.26		0	0.244							0.244		0.127	0.005		-
SA-33872	0	0.227	0.001	0.362	0	0.42	0	0	0	0	0.053	0.284	0	0	0.12	0.313	0.0002	0.053	0.001	-
AU-33873		0					0		0	0		0					0		0.089	-
OM-33874					0		0.255	0.256		0					0.015		0.335	0.084		-
SU-33951		0		0		0.158						0.922		0		0.920				-
WO-34310	0.177				0.098			0			-	-	-	-	-	-	-	-	-	-

CORE AREA (50%)

	DRY SEASON										WET SEASON									
	LION																			
SPOTTED HYENA	OK	RE	NU	MO	OJ	SU	OM*	LU	OF*	G2	OK	RE	NU	MO	OJ	SU	OM*	LU	OF*	G2
GO-33869			0.632	0									0.382	0						-
TJ-33870		0	0.239	0.717		0						0	0	0.328		0.038				-
NE-33871					0.033		0	0							0		0	0		-
SA-33872	0	0.004	0	0	0	0.40	0	0	0	0	0	0.253	0	0	0.021	0.315	0	0.044	0	-
AU-33873		0					0		0	0		0					0		0.103	-
OM-33874					0		0.004	0.002		0					0		0.183	0.003		-
SU-33951		0		0		0						0.481		0		0.741				-
WO-34310	0.063				0			0			-	-	-	-	-	-	-	-	-	-

(b)(i)

HOME RANGE (95%)

		DRY SEASON						WET SEASON					
		LION											
		SW-33950	AF-34308	BE-35678*	AM-36714	KW-36716*	KB-36717*	SW-33950	AF-34308	BE-35678*	AM-36714	KW-36716*	KB-36717*
SPOTTED HYENA	AR-33869*	-	-	-	-	-	-			0.656	0.594		
	IH-33870	0.566				0.034	0.250	0.707				0.178	0.291
	KW-33871*					0.432	0.011					0.782	0.047
	RV-33873*		0	0	0.681				0.572	0.113	0.955		
	SR-34310*	0.556				0.145	0.273	0.074				0.054	0.029

CORE AREA (50%)

		DRY SEASON						WET SEASON					
		LION											
		SW-33950	AF-34308	BE-35678*	AM-36714	KW-36716*	KB-36717*	SW-33950	AF-34308	BE-35678*	AM-36714	KW-36716*	KB-36717*
SPOTTED HYENA	AR-33869*	-	-	-	-	-	-			0	0		
	IH-33870	0.100				0	0	0				0	0
	KW-33871*					0	0					0	0
	RV-33873*		0	0	0				0	0	0.746		
	SR-34310*	0.117				0	0	0.122				0.017	0.030

(b)(ii)

		HOME RANGE (95%)											
		DRY SEASON						WET SEASON					
		LION											
		SW-33950	AF-34308	BE-35678*	AM-36714	KW-36716*	KB-36717*	SW-33950	AF-34308	BE-35678*	AM-36714	KW-36716*	KB-36717*
SPOTTED HYENA	AR-33869*	-	-	-	-	-	-			0.309	0.598		
	IH-33870	0.461				0.028	0.244	0.561				0.075	0.228
	KW-33871*					0.307	0					0.339	0.007
	RV-33873*		0	0	0.468				0.181	0	0.736		
	SR-34310*	0.416				0.100	0.262	0.222				0.022	0.067

		CORE AREA (50%)											
		DRY SEASON						WET SEASON					
		LION											
		SW-33950	AF-34308	BE-35678*	AM-36714	KW-36716*	KB-36717*	SW-33950	AF-34308	BE-35678*	AM-36714	KW-36716*	KB-36717*
SPOTTED HYENA	AR-33869*	-	-	-	-	-	-			0.0039	0		
	IH-33870	0.024				0	0	0				0	0
	KW-33871*					0	0					0	0
	RV-33873*		0	0	0				0	0	0		
	SR-34310*	0.105				0	0.034	0.003				0	0.0053
