# Supplementary material for: Coursing hyenas and stalking lions: The potential for inter- and intraspecific interactions
Source: PLoS One. 2023 Feb 3;18(2):e0265054. doi: 10.1371/journal.pone.0265054 (PMC9897591; doi:10.1371/journal.pone.0265054)
Supplement: S6 Table — T-tests comparing the distances between the center of individual core areas versus the competitors’ home range boundary in the Etosha National Park, Namibia (ENP) and the Chobe National Park and Linyanti Conservancy, Botswana (CNP). An asterisk denotes significance at the alpha level with * < 0.05, and *** < 0.001. (PDF) [file pone.0265054.s008.pdf]

S6 Table. Statistical results of the distances between competitor core areas and home range boundaries. *T*-tests comparing the distances between the center of individual core areas versus the competitors' home range boundary in the Etosha National Park, Namibia (ENP) and the Chobe National Park and Linyanti Conservancy, Botswana (CNP). An asterisk denotes significance at the alpha level with * < 0.05, and *** < 0.001.

Region	Measuring from center of core area to …	Distance (m)		Significance
**ENP**	Lion core – Lion core	41355.74 ± 21665.05	$t = 4.18$, df = 18, $p < 0.001$	***
	Lion core – Lion boundary	25375.28 ± 18970.48		
	Hyena core – Hyena core	53648.11 ± 26885.53	$t = 2.52$, df = 14, $p < 0.05$	*
	Hyena core – Hyena boundary	33676.46 ± 22906.57		
	Lion core – Hyena core	44238.06 ± 28973.78	$t = 6.46$, df = 17, $p < 0.001$	***
	Lion core – Hyena boundary	25309.90 ± 22395.97		
	Hyena core – Lion core	44238.06 ± 23381.01	$t = 1.66$, df = 14, $p > 0.05$	
	Hyena core – Lion boundary	29360.33 ± 20599.76		
**CNP**	Lion core – Lion core	62568.75 ± 48970.95	$t = 5.15$, df = 9, $p < 0.001$	***
	Lion core – Lion boundary	50034.22 ± 43051.36		
	Hyena core – Hyena core	67787.71 ± 43125.97	$t = 1.48$, df = 7, $p > 0.05$	
	Hyena core – Hyena boundary	55524.29 ± 45903.21		
	Lion core – Hyena core	55793.54 ± 47189.24	$t = 2.83$, df = 6, $p < 0.05$	*
	Lion core – Hyena boundary	44413.01 ± 46900.07		
	Hyena core – Lion core	55793.54 ± 46476.41	$t = 2.72$, df = 8, $p < 0.05$	*
	Hyena core – Lion boundary	42041.61 ± 41893.09		
