# Supplementary material for: Coursing hyenas and stalking lions: The potential for inter- and intraspecific interactions
Source: PLoS One. 2023 Feb 3;18(2):e0265054. doi: 10.1371/journal.pone.0265054 (PMC9897591; doi:10.1371/journal.pone.0265054)
Supplement: S7 Table — (a) all lions and spotted hyenas, and (b) lions and spotted hyenas from the Etosha National Park, Namibia (ENP) and the Chobe National Park, Linyanti Conservancy, and the NG32 concession of the Okavango Delta†, Botswana (CNP). Movement metrics consist of the means ± standard deviations for activity (activity monitor values [AMVs]), step length (m), speed (m/s), net-squared displacement (NSD, km2), and path tortuosity (radian). †No spotted hyenas were collared from the Okavango Delta, Botswana. (PDF) [file pone.0265054.s009.pdf]

S7 Table. Lion and spotted hyena movement metrics. (a) all lions and spotted hyenas, and (b) lions and spotted hyenas from the Etosha National Park, Namibia (ENP) and the Chobe National Park, Linyanti Conservancy, and the NG32 concession of the Okavango Delta†, Botswana (CNP). Movement metrics consist of the means ± standard deviations for activity (activity monitor values [AMVs]), step length (m), speed (m/s), net-squared displacement (NSD, km^2), and path tortuosity (radian).

†*No spotted hyenas were collared from the Okavango Delta, Botswana.*

(a)

Metric	Lion	Spotted hyena
Activity (AMVs in 24-hour period)	21.88 ± 41.2	40.20 ± 68
Nocturnal step length from 30min fixes (m)	289 ± 424	618 ± 685
Dusk/dawn step length from 5min fixes (m)	51 ± 82	122 ± 147

(b)

Metric		ENP		CNP	
		Lion	Spotted Hyena	Lion	Spotted Hyena
Speed (m/s)		0.187 ± 0.25	0.364 ± 0.39	0.128 ± 0.23	0.310 ± 0.35
24-hour step length from 4hr fixes (m)		1257 ± 1535	2009 ± 2156	898 ± 1159	1744 ± 1857
Nocturnal step length from 30min fixes (m)		308 ± 462	649 ± 685	208 ± 340	529 ± 570
Dusk/dawn step length from 5min fixes (m)		55 ± 99	130 ± 148	44 ± 78	119 ± 153
12-hour nocturnal NSD from 4hr fixes (km^2)		8.3 ± 19.1	17.9 ± 43.1	2.9 ± 9.1	6.2 ± 19.9
12-hour diurnal NSD from 4hr fixes (km^2)		2.6 ± 6.8	5.2 ± 21.7	1.1 ± 3.9	0.97 ± 3.7
12-hour nocturnal dry season NSD from 30min fixes (km^2)		12.4 ± 0.59	27.4 ± 0.78	5.2 ± 0.70	12.6 ± 0.53
12-hour nocturnal wet season NSD from 30min fixes (km^2)		12.8 ± 0.58	32.6 ± 1.2	6.9 ± 0.26	17.2 ± 0.58
24-hour path tortuosity (4hr fixes)		0.080 ± 2.07	-2.852 ± 2.39	0.432 ± 2.41	2.963 ± 2.18
	Dry season	0.070 ± 2.07	-2.012 ± 2.24	0.296 ± 2.33	2.660 ± 2.03
	Wet season	-0.022 ± 2.07	1.637 ± 2.60	0.209 ± 2.35	-2.942 ± 2.01
	Male	0.194 ± 1.98	-1.255 ± 2.50	-0.044 ± 2.63	-3.09 ± 1.87
	Female	0.029 ± 2.09	0.806 ± 2.39	0.612 ± 2.05	2.800 ± 2.27
Nocturnal path tortuosity (30min fixes)		0.135 ± 1.90	0.012 ± 1.73	0.052 ± 2.48	0.034 ± 1.87
	Dry season	0.018 ± 1.89	0.027 ± 1.84	-0.068 ± 2.44	-0.018 ± 1.87
	Wet season	0.151 ± 1.93	0.025 ± 1.68	0.003 ± 2.49	0.035 ± 1.87
	Male	0.046 ± 1.78	-0.053 ± 1.58	0.095 ± 2.46	0.069 ± 1.95
	Female	0.172 ± 1.92	0.021 ± 1.74	0.026 ± 2.52	0.011 ± 1.83
Dusk/dawn path tortuosity (5min fixes)		0.053 ± 2.43	0.013 ± 1.59	0.847 ± 1.92	0.001 ± 1.31
	Dry season	-0.012 ± 2.40	0.036 ± 1.68	0.024 ± 2.01	0.019 ± 1.42
	Wet season	0.097 ± 2.42	0.012 ± 1.54	1.062 ± 1.85	0.004 ± 1.25
	Male	-0.094 ± 2.33	-0.011 ± 1.43	3.067 ± 1.82	-0.026 ± 1.07
	Female	0.123 ± 2.45	0.017 ± 1.60	0.448 ± 2.19	0.019 ± 1.43
