# Supplementary material for: Coursing hyenas and stalking lions: The potential for inter- and intraspecific interactions
Source: PLoS One. 2023 Feb 3;18(2):e0265054. doi: 10.1371/journal.pone.0265054 (PMC9897591; doi:10.1371/journal.pone.0265054)
Supplement: S8 Table — Activity (AMVs), step length (m), and path tortuosity (radian) of lions and spotted hyenas from the Etosha National Park, Namibia (ENP), and the Chobe National Park, Linyanti Conservancy, and the NG32 concession of the Okavango Delta†, Botswana (CNP). Values are means ± standard deviations during the nocturnal (30min fixes from 18h00-6h00 and 17h00-8h00) and dusk/dawn (5min fixes from 19h00-21h00 and 4h00-6h00) periods. †No spotted hyenas were collared from the Okavango Delta, Botswana. (PDF) [file pone.0265054.s010.pdf]

S8 Table. Lion and spotted hyena activity and movement metrics in relation to the lunar cycle. Activity (AMVs), step length (m), and path tortuosity (radian) of lions and spotted hyenas from the Etosha National Park, Namibia (ENP), and the Chobe National Park, Linyanti Conservancy, and the NG32 concession of the Okavango Delta†, Botswana (CNP). Values are means ± standard deviations during the nocturnal (30min fixes from 18h00-6h00 and 17h00-8h00) and dusk/dawn (5min fixes from 19h00-21h00 and 4h00-6h00) periods.

†*No spotted hyenas were collared from the Okavango Delta, Botswana.*

Period	Lunar phase	Activity				Step length (m)				Tortuosity			
		Lion		Spotted Hyena		Lion		Spotted Hyena		Lion		Spotted Hyena	
		ENP	CNP	ENP	CNP	ENP	CNP	ENP	CNP	ENP	CNP	ENP	CNP
Nocturnal combined seasons	**New moon**	32.67 ± 49.27	29.34 ± 45.53	76.37 ± 74.82	70.18 ± 65.65	305.29 ± 430.55	242.81 ± 414.56	622.07 ± 702.96	576.63 ± 645.44	0.367 ± 2.21	-0.272 ± 2.26	-0.049 ± 1.79	-0.015 ± 1.88
	**Full moon**	33.39 ± 48.98	27.16 ± 44.46	79.65 ± 75.11	64.40 ± 64.83	333.58 ± 442.03	222.71 ± 394.73	680.66 ± 715.93	565.66 ± 626.43	-0.011 ± 1.81	0.442 ± 2.58	0.001 ± 1.70	0.041 ± 1.92
Nocturnal dry season	**New moon**	38.46 ± 52.31	30.28 ± 45.85	78.11 ± 77.72	59.39 ± 65.62	304.10 ± 453.23	286.42 ± 409.05	659.76 ± 697.12	543.53 ± 634.24	0.351 ± 1.93	-0.162 ± 2.29	-0.075 ± 1.94	0.139 ± 1.88
	**Waxing crescent**	45.35 ± 53.94	31.19 ± 47.54	73.21 ± 77.41	49.03 ± 59.45	400.64 ± 472.46	275.59 ± 425.23	635.33 ± 713.10	505.70 ± 617.45	0.014 ± 1.82	-0.030 ± 2.24	0.066 ± 1.83	-0.029 ± 1.82
	**First quarter**	27.17 ± 48.32	27.73 ± 44.82	65.85 ± 76.68	55.17 ± 59.19	288.77 ± 437.63	263.57 ± 414.39	676.00 ± 716.69	490.43 ± 624.94	-0.170 ± 2.03	0.077 ± 2.32	0.139 ± 1.78	-0.057 ± 1.77
	**Waxing gibbous**	23.75 ± 45.48	30.39 ± 49.22	86.40 ± 80.02	72.68 ± 71.36	243.85 ± 400.09	289.05 ± 458.72	739.00 ± 742.85	534.73 ± 667.53	-0.245 ± 2.05	-0.243 ± 2.29	-0.194 ± 1.72	-0.046 ± 1.85
	**Full moon**	33.23 ± 50.35	26.42 ± 44.38	85.07 ± 80.38	68.51 ± 69.09	327.22 ± 456.94	223.80 ± 384.66	724.96 ± 766.69	540.41 ± 643.28	0.013 ± 1.88	-0.024 ± 2.49	0.101 ± 1.83	0.020 ± 1.92
	**Waning gibbous**	36.16 ± 50.15	27.85 ± 47.76	66.01 ± 79.53	54.22 ± 64.02	330.55 ± 430.07	242.42 ± 409.72	662.18 ± 739.07	532.14 ± 668.52	0.164 ± 1.82	-0.057 ± 2.36	0.089 ± 1.81	0.026 ± 1.89
	**Last quarter**	37.39 ± 52.07	32.60 ± 46.91	75.38 ± 77.10	57.26 ± 62.45	334.28 ± 449.32	277.93 ± 397.18	677.94 ± 728.41	464.26 ± 585.57	-0.223 ± 1.80	-0.135 ± 2.56	-0.017 ± 1.77	0.075 ± 1.93
	**Waning crescent**	37.22 ± 53.67	29.47 ± 49.82	73.23 ± 75.08	65.82 ± 70.23	389.16 ± 479.82	253.94 ± 438.29	642.67 ± 709.89	465.94 ± 633.93	-0.009 ± 2.00	0.259 ± 2.30	0.023 ± 1.92	0.021 ± 1.94
Nocturnal wet season	**New moon**	29.41 ± 46.60	27.87 ± 45.19	73.84 ± 71.91	80.92 ± 63.90	280.48 ± 405.82	229.89 ± 414.66	612.65 ± 701.19	610.08 ± 642.25	0.279 ± 2.40	-0.128 ± 2.18	-0.089 ± 1.77	-0.035 ± 1.92
	**Waxing crescent**	34.29 ± 49.79	27.51 ± 44.07	77.15 ± 71.30	76.06 ± 59.17	333.92 ± 443.52	221.28 ± 408.04	676.41 ± 700.03	595.96 ± 631.11	0.094 ± 1.90	-0.182 ± 2.38	-0.043 ± 1.60	-0.009 ± 1.80
	**First quarter**	34.05 ± 48.81	24.31 ± 41.38	79.06 ± 71.52	80.88 ± 70.23	337.00 ± 455.68	210.67 ± 385.91	693.34 ± 690.07	531.91 ± 618.95	-0.085 ± 1.87	-0.024 ± 2.53	0.084 ± 1.61	0.432 ± 1.85
	**Waxing gibbous**	36.90 ± 50.21	25.59 ± 44.34	74.46 ± 71.45	67.54 ± 64.49	380.32 ± 461.24	213.71 ± 414.33	678.80 ± 691.21	544.71 ± 598.91	0.200 ± 1.91	0.377 ± 2.41	0.009 ± 1.71	-0.008 ± 1.86
	**Full moon**	32.68 ± 47.55	27.30 ± 43.68	75.49 ± 69.68	59.11 ± 58.48	318.44 ± 428.26	221.29 ± 397.06	663.43 ± 671.91	566.62 ± 622.22	0.017 ± 1.76	0.674 ± 2.49	0.010 ± 1.65	-0.142 ± 1.92
	**Waning gibbous**	36.53 ± 51.71	23.79 ± 41.63	75.74 ± 70.51	59.16 ± 61.74	342.38 ± 458.54	201.83 ± 387.32	679.31 ± 670.25	572.46 ± 639.97	0.139 ± 1.78	0.519 ± 2.60	0.028 ± 1.67	-0.005 ± 1.79

	**Last quarter**	36.05 ± 51.51	28.79 ± 44.52	74.79 ± 71.14	70.74 ± 70.15	333.92 ± 473.36	219.99 ± 397.79	641.18 ± 674.57	597.52 ± 642.67	-0.110 ± 1.92	0.230 ± 2.35	0.137 ± 1.74	0.049 ± 1.81
	**Waning crescent**	34.63 ± 49.27	28.29 ± 44.71	70.94 ± 72.39	74.34 ± 58.46	301.41 ± 429.88	216.85 ± 393.75	616.88 ± 679.42	590.43 ± 633.41	0.072 ± 2.10	0.127 ± 2.33	0.059 ± 1.74	-0.198 ± 1.93
Dusk/dawn combined seasons	**New moon**	32.25 ± 48.36	31.44 ± 47.38	74.68 ± 73.48	66.61 ± 64.09	54.69 ± 87.62	41.51 ± 78.48	117.07 ± 151.05	116.64 ± 138.92	-1.173 ± 2.38	0.844 ± 1.91	0.011 ± 1.66	-0.023 ± 1.36
	**Full moon**	34.49 ± 47.63	28.53 ± 44.50	79.25 ± 73.26	66.91 ± 63.48	61.84 ± 87.42	38.73 ± 75.12	130.90 ± 154.58	117.80 ± 140.95	-0.033 ± 2.44	1.506 ± 1.74	0.011 ± 1.57	-0.008 ± 1.31
Dusk/dawn dry season	**New moon**	40.25 ± 52.00	33.80 ± 46.61	76.43 ± 76.73	54.56 ± 60.78	55.26 ± 94.10	42.43 ± 77.68	123.08 ± 152.44	96.66 ± 128.23	-0.654 ± 2.47	-0.023 ± 1.94	0.074 ± 1.90	-0.111 ± 1.51
	**Waxing crescent**	44.32 ± 53.96	32.81 ± 47.82	77.99 ± 75.64	54.85 ± 61.29	77.17 ± 104.41	44.64 ± 83.82	128.91 ± 151.58	94.87 ± 135.97	0.038 ± 2.19	-0.356 ± 1.92	0.133 ± 1.65	0.060 ± 1.47
	**First quarter**	24.61 ± 48.13	32.81 ± 45.07	72.86 ± 75.03	62.01 ± 63.02	50.38 ± 94.18	46.39 ± 82.31	134.27 ± 152.94	97.98 ± 128.66	-3.045 ± 2.43	-0.539 ± 1.87	0.024 ± 1.58	0.047 ± 1.36
	**Waxing gibbous**	24.67 ± 44.71	32.34 ± 49.42	84.84 ± 78.97	68.70 ± 73.40	45.27 ± 84.72	45.78 ± 85.88	139.50 ± 166.50	109.04 ± 143.66	-0.106 ± 2.26	-0.038 ± 2.07	-0.007 ± 1.74	0.034 ± 1.33
	**Full moon**	34.56 ± 48.77	29.13 ± 44.30	86.23 ± 79.98	65.82 ± 66.37	52.43 ± 89.88	39.62 ± 75.64	139.76 ± 169.42	99.97 ± 136.05	-0.054 ± 2.45	2.994 ± 1.75	-0.068 ± 1.74	-0.013 ± 1.38
	**Waning gibbous**	34.39 ± 49.85	35.13 ± 50.05	70.62 ± 77.86	59.98 ± 62.19	58.02 ± 93.94	42.26 ± 80.11	134.31 ± 157.50	104.37 ± 138.26	0.141 ± 2.27	1.068 ± 1.94	0.023 ± 1.58	0.014 ± 1.40
	**Last quarter**	34.85 ± 50.04	34.75 ± 48.97	79.34 ± 75.24	55.45 ± 62.82	59.50 ± 96.01	41.92 ± 75.98	139.84 ± 153.21	89.95 ± 123.05	0.147 ± 2.22	-0.013 ± 1.77	-0.028 ± 1.57	-0.161 ± 1.60
	**Waning crescent**	35.05 ± 52.38	33.54 ± 51.74	78.63 ± 74.78	64.19 ± 69.48	64.30 ± 100.82	39.75 ± 84.56	123.89 ± 146.00	89.77 ± 126.34	-0.011 ± 2.31	0.354 ± 2.03	0.008 ± 1.72	0.099 ± 1.44
Dusk/dawn wet season	**New moon**	30.13 ± 45.49	30.71 ± 47.94	71.65 ± 70.10	79.33 ± 65.06	53.28 ± 82.14	42.73 ± 78.83	115.69 ± 148.34	123.85 ± 144.87	-2.659 ± 2.25	0.785 ± 1.92	0.012 ± 1.58	0.001 ± 1.29
	**Waxing crescent**	35.09 ± 48.60	31.83 ± 44.60	75.58 ± 68.15	79.60 ± 61.14	62.14 ± 89.65	39.92 ± 74.88	128.85 ± 138.92	123.54 ± 147.11	-0.163 ± 2.35	0.862 ± 1.85	0.008 ± 1.51	0.015 ± 1.22
	**First quarter**	36.24 ± 49.59	29.70 ± 43.85	77.79 ± 69.73	93.75 ± 69.60	68.19 ± 94.63	38.14 ± 74.58	125.10 ± 142.73	116.65 ± 151.16	-0.107 ± 2.29	1.818 ± 1.93	0.030 ± 1.53	0.069 ± 1.22
	**Waxing gibbous**	39.36 ± 51.94	31.46 ± 48.08	73.85 ± 69.95	71.06 ± 62.73	75.70 ± 99.99	39.58 ± 80.33	129.37 ± 148.14	105.13 ± 138.76	-0.195 ± 2.20	0.145 ± 1.76	-0.010 ± 1.54	-0.041 ± 1.24
	**Full moon**	34.21 ± 46.13	29.44 ± 44.54	72.38 ± 66.30	68.25 ± 59.75	58.39 ± 84.40	38.16 ± 74.63	124.89 ± 137.49	102.68 ± 145.11	-0.228 ± 2.11	1.232 ± 1.73	0.031 ± 1.50	-0.089 ± 1.25
	**Waning gibbous**	34.27 ± 50.36	28.11 ± 43.15	73.51 ± 69.59	65.92 ± 67.60	59.76 ± 93.85	35.84 ± 73.21	121.07 ± 139.68	124.33 ± 155.93	0.180 ± 2.30	-1.526 ± 1.77	0.040 ± 1.53	0.021 ± 1.30
	**Last quarter**	33.68 ± 48.72	32.28 ± 46.56	71.73 ± 68.01	78.36 ± 69.36	57.81 ± 93.29	38.61 ± 73.96	113.50 ± 131.89	125.62 ± 146.76	-0.282 ± 2.42	0.988 ± 1.81	-0.018 ± 1.54	-0.009 ± 1.23
	**Waning crescent**	30.61 ± 44.76	33.68 ± 46.29	70.30 ± 70.49	76.18 ± 58.79	49.69 ± 83.14	43.17 ± 76.29	118.35 ± 145.10	128.95 ± 146.78	0.037 ± 2.41	-1.314 ± 1.78	-0.010 ± 1.59	-0.017 ± 1.26
