# Supplementary material for: Coursing hyenas and stalking lions: The potential for inter- and intraspecific interactions
Source: PLoS One. 2023 Feb 3;18(2):e0265054. doi: 10.1371/journal.pone.0265054 (PMC9897591; doi:10.1371/journal.pone.0265054)
Supplement: S9 Table — Activity (AMVs) of lions and spotted hyenas from the Etosha National Park, Namibia (ENP), and the Chobe National Park, Linyanti Conservancy, and the NG32 concession of the Okavango Delta†, Botswana (CNP). The 24-hour cycle was subdivided into seven different periods. Night/nadir/night end consists of the end of evening twilight to the beginning of morning twilight divided into three equal intervals. Afternoon = noon to sundown; dusk = sundown to twilight end; dawn = beginning of morning twilight to sunrise; morning = sunrise to noon. Values indicate the means ± standard deviations of the seven different periods of the 24-hour cycle during new and full moon phases of the dry and wet seasons. †No spotted hyenas were collared from the Okavango Delta, Botswana. (PDF) [file pone.0265054.s011.pdf]

**S9 Table. New and full moon activity of lions and spotted hyenas from the 24-hour period cycle.** Activity (AMVs) of lions and spotted hyenas from the Etosha National Park, Namibia (ENP), and the Chobe National Park, Linyanti Conservancy, and the NG32 concession of the Okavango Delta†, Botswana (CNP). The 24-hour cycle was subdivided into seven different periods. Night/nadir/night end consists of the end of evening twilight to the beginning of morning twilight divided into three equal intervals. Afternoon = noon to sundown; dusk = sundown to twilight end; dawn = beginning of morning twilight to sunrise; morning = sunrise to noon. Values indicate the means ± standard deviations of the seven different periods of the 24-hour cycle during new and full moon phases of the dry and wet seasons.

†*No spotted hyenas were collared from the Okavango Delta, Botswana.*

| Season | 24-hour period cycle | New Moon | | | | Full Moon | | | |
|---|---|---|---|---|---|---|---|---|---|
| | | Lion | | Spotted Hyena | | Lion | | Spotted Hyena | |
| | | ENP | CNP | ENP | CNP | ENP | CNP | ENP | CNP |
| Dry season | **Afternoon** | 6.49 ± 11.93 | 5.47 ± 10.30 | 4.95 ± 10.68 | 6.01 ± 4.03 | 4.97 ± 9.87 | 6.10 ± 11.53 | 3.64 ± 8.67 | 5.39 ± 3.20 |
| | **Dusk** | 24.35 ± 40.59 | 31.30 ± 48.54 | 68.12 ± 61.88 | 24.05 ± 15.10 | 23.20 ± 43.14 | 25.90 ± 44.05 | 74.17 ± 65.99 | 34.81 ± 17.41 |
| | **Night** | 33.72 ± 34.28 | 25.07 ± 30.98 | 91.80 ± 42.73 | 45.43 ± 12.77 | 25.67 ± 32.85 | 20.35 ± 28.26 | 104.43 ± 48.12 | 54.49 ± 16.13 |
| | **Nadir** | 41.01 ± 38.98 | 30.31 ± 32.03 | 81.54 ± 47.29 | 61.49 ± 17.05 | 35.95 ± 38.61 | 28.52 ± 32.80 | 94.11 ± 48.88 | 71.81 ± 17.24 |
| | **Night end** | 41.64 ± 39.22 | 31.60 ± 33.84 | 73.92 ± 46.38 | 75.15 ± 15.45 | 39.63 ± 38.25 | 29.77 ± 33.69 | 84.27 ± 44.80 | 87.56 ± 16.65 |
| | **Dawn** | 34.07 ± 51.66 | 38.74 ± 51.01 | 79.21 ± 59.44 | 82.73 ± 25.09 | 39.02 ± 55.81 | 32.40 ± 47.98 | 71.70 ± 62.64 | 87.65 ± 23.31 |
| | **Morning** | 11.67 ± 17.31 | 15.20 ± 19.70 | 19.10 ± 24.28 | 17.55 ± 8.41 | 13.98 ± 20.63 | 11.98 ± 18.01 | 15.05 ± 23.77 | 15.61 ± 8.75 |
| Wet season | **Afternoon** | 8.12 ± 13.99 | 9.48 ± 14.32 | 9.20 ± 16.40 | 6.23 ± 3.87 | 6.93 ± 13.03 | 10.54 ± 14.25 | 7.46 ± 14.97 | 3.66 ± 2.84 |
| | **Dusk** | 31.48 ± 55.97 | 34.01 ± 48.31 | 76.52 ± 64.24 | 53.71 ± 18.24 | 30.42 ± 44.76 | 37.52 ± 48.07 | 76.06 ± 60.70 | 37.23 ± 15.23 |
| | **Night** | 31.14 ± 43.57 | 30.69 ± 31.50 | 84.21 ± 49.53 | 75.03 ± 13.06 | 28.17 ± 34.34 | 25.15 ± 33.04 | 86.71 ± 42.02 | 54.44 ± 11.13 |
| | **Nadir** | 31.52 ± 44.39 | 28.56 ± 33.30 | 78.38 ± 52.26 | 99.62 ± 16.77 | 35.06 ± 40.30 | 23.73 ± 30.56 | 88.13 ± 48.66 | 61.12 ± 12.63 |
| | **Night end** | 28.51 ± 40.47 | 25.82 ± 34.28 | 73.89 ± 46.56 | 102.01 ± 17.15 | 34.91 ± 39.89 | 25.91 ± 32.34 | 74.29 ± 44.51 | 72.71 ± 13.08 |
| | **Dawn** | 28.02 ± 50.06 | 24.47 ± 43.64 | 74.66 ± 54.77 | 94.28 ± 21.70 | 28.73 ± 48.65 | 22.54 ± 36.63 | 74.97 ± 62.14 | 95.17 ± 14.82 |
| | **Morning** | 11.70 ± 20.30 | 10.50 ± 15.62 | 22.74 ± 28.79 | 15.90 ± 7.77 | 10.61 ± 17.86 | 10.31 ± 14.98 | 18.80 ± 26.16 | 15.67 ± 8.22 |
