# Supplementary material for: Coursing hyenas and stalking lions: The potential for inter- and intraspecific interactions
Source: PLoS One. 2023 Feb 3;18(2):e0265054. doi: 10.1371/journal.pone.0265054 (PMC9897591; doi:10.1371/journal.pone.0265054)
Supplement: S10 Table — Speed (m/s) and path tortuosity (radian) of lions and spotted hyenas during nocturnal (30min fixes) and dusk/dawn (5min fixes) periods from the Etosha National Park, Namibia, the Chobe National Park, Linyanti Conservancy, and the NG32 concession of the Okavango Delta†, Botswana. Values are indicated in means ± standard deviations. Body conditions of individuals were scored from spinal palpations of immobilized individuals during capture events. †No spotted hyenas were collared from the Okavango Delta, Botswana. (PDF) [file pone.0265054.s012.pdf]

S10 Table. Lion and spotted hyena movement metrics according to low, medium, and high body condition scores. Speed (m/s) and path tortuosity (radian) of lions and spotted hyenas during nocturnal (30min fixes) and dusk/dawn (5min fixes) periods from the Etosha National Park, Namibia, the Chobe National Park, Linyanti Conservancy, and the NG32 concession of the Okavango Delta†, Botswana. Values are indicated in means ± standard deviations. Body conditions of individuals were scored from spinal palpations of immobilized individuals during capture events.
†*No spotted hyenas were collared from the Okavango Delta, Botswana.*

Period	BCS	Speed (m/s)				Tortuosity			
		Lion	Male lion	Female lion	Spotted Hyena	Lion	Male lion	Female lion	Spotted Hyena
Nocturnal periods	**Low BCS**	0.195 ± 0.26	0.221 ± 0.31	0.181 ± 0.25	-	-0.016 ± 1.86	0.052 ± 1.66	-0.050 ± 1.89	-
	**Medium BCS**	0.153 ± 0.23	0.195 ± 0.28	0.136 ± 0.22	0.328 ± 0.36	0.044 ± 2.28	0.042 ± 1.81	0.044 ± 2.34	0.043 ± 1.83
	**High BCS**	0.145 ± 0.22	0.108 ± 0.20	0.169 ± 0.24	0.377 ± 0.45	0.081 ± 2.37	0.095 ± 2.46	0.068 ± 2.02	-0.031 ± 1.60
Dusk/dawn periods	**Low BCS**	0.217 ± 0.35	0.253 ± 0.39	0.200 ± 0.34	-	-0.023 ± 2.29	0.008 ± 1.91	-0.038 ± 2.34	-
	**Medium BCS**	0.153 ± 0.25	0.220 ± 0.35	0.134 ± 0.24	0.393 ± 0.46	0.141 ± 2.34	-0.145 ± 2.41	0.284 ± 2.33	0.015 ± 1.50
	**High BCS**	0.173 ± 0.28	0.142 ± 0.27	0.205 ± 0.32	0.433 ± 0.57	0.037 ± 1.87	3.067 ± 1.82	-0.000 ± 2.30	-0.007 ± 1.44
