# Supplementary material for: Coursing hyenas and stalking lions: The potential for inter- and intraspecific interactions
Source: PLoS One. 2023 Feb 3;18(2):e0265054. doi: 10.1371/journal.pone.0265054 (PMC9897591; doi:10.1371/journal.pone.0265054)
Supplement: S11 Table — Speed (m/s) and path tortuosity (radian) of lions and spotted hyenas during nocturnal (30min fixes) and dusk/dawn (5min fixes) periods according to the probability of site-attracted foragers (i.e. ungulates) in sites that consisted of anthrax positive carcasses from previous years. (PDF) [file pone.0265054.s013.pdf]

S11 Table. Lion and spotted hyena movement metrics according to site-attracted foragers in the Etosha National Park, Namibia. Speed (m/s) and path tortuosity (radian) of lions and spotted hyenas during nocturnal (30min fixes) and dusk/dawn (5min fixes) periods according to the probability of site-attracted foragers (i.e., ungulates) in sites that consisted of anthrax positive carcasses from previous years.

Period	Probability of site-attracted foragers	Speed (m/s)		Tortuosity	
		Lion	Spotted Hyena	Lion	Spotted Hyena
Nocturnal periods	**0-20%**	0.181 ± 0.24	0.410 ± 0.37	0.088 ± 1.91	0.012 ± 1.77
	**20-40%**	0.198 ± 0.27	0.456 ± 0.46	0.088 ± 1.78	-0.216 ± 1.85
	**40-60%**	0.230 ± 0.28	0.417 ± 0.46	0.089 ± 1.74	0.039 ± 1.77
	**60-80%**	0.167 ± 0.26	0.460 ± 0.47	-0.225 ± 1.93	-0.037 ± 1.54
	**80-100%**	0.226 ± 0.28	0.470 ± 0.43	0.079 ± 2.18	-0.113 ± 1.49
Dusk/dawn periods	**0-20%**	0.220 ± 0.29	0.484 ± 0.46	-0.021 ± 2.50	0.015 ± 1.70
	**20-40%**	0.213 ± 0.35	0.556 ± 0.60	0.011 ± 2.17	0.004 ± 1.36
	**40-60%**	0.241 ± 0.33	0.447 ± 0.56	0.008 ± 2.22	0.020 ± 1.69
	**60-80%**	0.187 ± 0.33	0.528 ± 0.62	0.014 ± 2.51	-0.007 ± 1.33
	**80-100%**	0.214 ± 0.40	0.536 ± 0.58	-0.034 ± 2.45	-0.043 ± 1.32
