# Supplementary material for: Coursing hyenas and stalking lions: The potential for inter- and intraspecific interactions
Source: PLoS One. 2023 Feb 3;18(2):e0265054. doi: 10.1371/journal.pone.0265054 (PMC9897591; doi:10.1371/journal.pone.0265054)
Supplement: S12 Table — Chi-square and t-test results for the percent frequency occurrence of, and average distance in meters to, the nearest conspecific and competitor for each bin of distance intervals. An asterisk denotes significance at the alpha level with * < 0.05, ** < 0.01, *** < 0.005, and **** < 0.001. (PDF) [file pone.0265054.s014.pdf]

S12 Table. Statistical results to accompany Fig 7 in the main text. Chi-square and *t*-test results for the percent frequency occurrence of, and average distance in meters to, the nearest conspecific and competitor for each bin of distance intervals. An asterisk denotes significance at the alpha level with * < 0.05, ** < 0.01, *** < 0.005, and **** < 0.001.

Region & Species	Distance Interval (m)	Conspecific *Percent frequency occurrence / Average distance to (m)*	Competitor *Percent frequency occurrence / Average distance to (m)*		Significance
Etosha Lion	*0 – 5000*	*47%*	*53%*	*χ^2 = 0.35, df = 1, p > 0.05*	
	1000 – 5000	35%<br>3396 ± 688	89%<br>3560 ± 450	χ^2 = 23.85, df = 1, p < 0.001<br>*t* = -0.66, df = 11, p > 0.05	****
	500 – 1000	3%<br>730 ± 54	5%<br>763 ± 62	χ^2= 0.30, df = 1, p > 0.05<br>*t* = -1.08, df = 5, p > 0.05	
	200 – 500	3%<br>327 ± 17	3%<br>376 ± 38	χ^2 = 0.01, df = 1, p > 0.05<br>*t* = -3.86, df = 12, p < 0.005	 ***
	100 – 200	3%<br>156 ± 26	2%<br>154 ± 18	χ^2 = 0.27, df = 1, p > 0.05<br>*t* = 0.11, df = 4, p > 0.05	
	50 – 100	5%<br>69 ± 3	1%<br>80 ± 11	χ^2 = 3.29, df = 1, p > 0.05<br>*t* = -3.07, df = 11, p < 0.05	 *
	10 – 50	28%<br>27 ± 6	0.3%<br>32 ± 8	χ^2 = 27.12, df = 1, p < 0.001<br>*t* = -1.28, df = 8, p > 0.05	****
	0 – 10	23%<br>6 ± 3	0.01%<br>8	χ^2 = 22.84, df = 1, p < 0.001<br>*t* = N/A	****
Chobe Lion	*0 – 5000*	*37%*	*63%*	*χ^2 = 7.08, df = 1, p < 0.01*	**
	1000 – 5000	85%<br>3315 ± 781	95%<br>3730 ± 407	χ^2 = 0.61, df = 1, p > 0.05<br>*t* = -1.22, df = 6, p > 0.05	
	500 – 1000	4%<br>785 ± 92	3%<br>823 ± 94	χ^2 = 0.29, df = 1, p > 0.05<br>*t* = -0.74, df = 9, p > 0.05	
	200 – 500	3%<br>307 ± 49	1%<br>334 ± 32	χ^2 = 0.49, df = 1, p > 0.05<br>*t* = -1.08, df = 7, p > 0.05	
	100 – 200	1%<br>152 ± 13	0.4%<br>148 ± 10	χ^2 = 0.55, df = 1, p > 0.05<br>*t* = 0.51, df = 5, p > 0.05	
	50 – 100	1%<br>71 ± 14	0.2%<br>77 ± 15	χ^2 = 0.55, df = 1, p > 0.05<br>*t* = -0.72, df = 4, p > 0.05	
	10 – 50	3%<br>34 ± 9	0.4%<br>33 ± 2	χ^2 = 2.55, df = 1, p > 0.05<br>*t* = 0.33, df = 2, p > 0.05	
	0 – 10	3%<br>6 ± 2	0<br>NA	χ^2 = 3.24, df = 1, p > 0.05<br>*t* = N/A	
Etosha Hyena	*0 – 5000*	*6%*	*94%*	*χ^2 = 76.53, df = 1, p < 0.001*	****
	1000 – 5000	92.5%<br>3502 ± 343	89%<br>3560 ± 450	χ^2 = 0.07, df = 1, p > 0.05<br>*t* = -0.37, df = 13, p > 0.05	
	500 – 1000	3.9%<br>804 ± 121	4.7%<br>763 ± 62	χ^2 = 0.09, df = 1, p > 0.05<br>*t* = 0.78, df = 6, p > 0.05	
	200 – 500	1.8%<br>339 ± 48	3.2%<br>376 ± 38	χ^2 = 0.40, df = 1, p > 0.05<br>*t* = -1.56, df = 6, p > 0.05	
	100 – 200	0.7%<br>158 ± 20	1.9%<br>154 ± 18	χ^2 = 0.61, df = 1, p > 0.05<br>*t* = 0.29, df = 5, p > 0.05	
	50 – 100	0.3%<br>65 ± 4	0.8%<br>80 ± 11	χ^2 = 0.19, df = 1, p > 0.05<br>*t* = -3.37, df = 4, p < 0.05	 *

	10 – 50	0.6%<br>27 ± 13	0.3%<br>32 ± 8	$\varkappa^2$ = 0.12, df = 1, p > 0.05<br>*t* = -0.64, df = 4, p > 0.05	
	0 – 10	0.2%<br>3	0.01%<br>8	$\varkappa^2$ = 0.21, df = 1, p > 0.05<br>*t* = N/A	
Chobe Hyena	*0 – 5000*	*26%*	*74%*	*$\varkappa^2$ = 23.37, df = 1, p < 0.001*	****
	1000 – 5000	71.8%<br>2842 ± 1192	95.3%<br>3730 ± 407	$\varkappa^2$ = 3.31, df = 1, p > 0.05<br>t = -7.24, df = 13, p < 0.001	 ****
	500 – 1000	12.8%<br>749 ± 142	2.8%<br>823 ± 94	$\varkappa^2$ = 6.43, df = 1, p < 0.05<br>*t* = -2.28, df = 10, p < 0.05	*<br>*
	200 – 500	8.3%<br>341 ± 87	1.2%<br>334 ± 32	$\varkappa^2$ = 5.28, df = 1, p < 0.05<br>*t* = 0.49, df = 9, p > 0.05	*
	100 – 200	2.6%<br>150 ± 30	0.4%<br>148 ± 10	$\varkappa^2$ = 1.59, df = 1, p > 0.05<br>*t* = 0.26, df = 16, p > 0.05	
	50 – 100	2.2%<br>68 ± 14	0.2%<br>77 ± 15	$\varkappa^2$ = 1.66, df = 1, p > 0.05<br>*t* = -1.09, df = 4, p > 0.05	
	10 – 50	1.7%<br>26 ± 12	0.1%<br>33 ± 2	$\varkappa^2$ = 1.57, df = 1, p > 0.05<br>*t* = -2.50, df = 12, p < 0.05	 *
	0 – 10	0.6%<br>6	0<br>NA	$\varkappa^2$ = 0.63, df = 1, p > 0.05<br>*t* = N/A	
