# Supplementary material for: Coursing hyenas and stalking lions: The potential for inter- and intraspecific interactions
Source: PLoS One. 2023 Feb 3;18(2):e0265054. doi: 10.1371/journal.pone.0265054 (PMC9897591; doi:10.1371/journal.pone.0265054)
Supplement: S13 Table — T-tests of consecutive time points among dyads (indicating longer time duration) at various distance intervals (a), and the frequency occurrences of dyads for different consecutive time points (b). An asterisk denotes significance at the alpha level with * < 0.05, ** < 0.01, *** < 0.005, and **** < 0.001. (PDF) [file pone.0265054.s015.pdf]

S13 Table. Statistical results to accompany Fig 8 in the main text. *T*-tests of consecutive time points among dyads (indicating longer time duration) at various distance intervals (a), and the frequency occurrences of dyads for different consecutive time points (b). An asterisk denotes significance at the alpha level with * < 0.05, ** < 0.01, *** < 0.005, and **** < 0.001.

(a)

Dyads	Distance Intervals (m)		Significance
LN-LN vs LN-HY	0-100	t = 4.31, df = 5, p < 0.01	**
	100-200	t = 2.94, df = 6, p < 0.05	*
	200-500	t = 3.19, df = 10, p < 0.01	**
	500-1000	t = 2.65, df = 8, p < 0.05	*
	1000-2000	t = 1.50, df = 9, p = 0.167	
HY-HY vs LN-HY	0-100	t = 1.27, df = 5, p = 0.257	
	100-200	t = 1.10, df = 5, p = 0.317	
	200-500	t = 1.51, df = 6, p = 0.186	
	500-1000	t = 1.89, df = 6, p = 0.107	
	1000-2000	t = 1.79, df = 8, p = 0.112	
LN-LN vs HY-HY	0-100	t = 1.70, df = 9, p = 0.121	
	100-200	t = 0.45, df = 8, p = 0.665	
	200-500	t = -0.46, df = 6, p = 0.663	
	500-1000	t = -0.94, df = 5, p = 0.386	
	1000-2000	t = -0.92, df = 7, p = 0.388	

(b)

Dyads	Consecutive Time Points		Significance
LN-LN vs LN-HY	1	t = 0.73, df = 5, p = 0.497	
	2	t = 1.47, df = 8, p = 0.180	
	3-5	t = 6.47, df = 6, p < 0.001	****
	6-10	t = 1.93, df = 6, p = 0.106	
	11-30	t = 2.70, df = 8, p < 0.05	*
	>30	t = 6.60, df = 4, p < 0.005	***
HY-HY vs LN-HY	1	t = 4.83, df = 8, p < 0.005	***
	2	t = 3.37, df = 7, p < 0.05	*
	3-5	t = 2.38, df = 4, p = 0.074	
	6-10	t = 1.36, df = 5, p = 0.240	
	11-30	t = -0.15, df = 8, p = 0.888	
	>30	t = -3.50, df = 4, p < 0.05	*
LN-LN vs HY-HY	1	t = -5.91, df = 6, p < 0.005	***
	2	t = -2.47, df = 6, p < 0.05	*
	3-5	t = -0.30, df = 5, p = 0.778	
	6-10	t = 0.23, df = 6, p = 0.828	
	11-30	t = 2.70, df = 8, p < 0.05	*
	>30	t = 6.99, df = 4, p < 0.005	***
