# Supplementary material for: Coursing hyenas and stalking lions: The potential for inter- and intraspecific interactions
Source: PLoS One. 2023 Feb 3;18(2):e0265054. doi: 10.1371/journal.pone.0265054 (PMC9897591; doi:10.1371/journal.pone.0265054)
Supplement: S14 Table — Nocturnal (30min fixes) and dusk/dawn (5min fixes) periods of lion and spotted hyena activity (AMVs), speed (m/s), and path tortuosity (radian) from (a) inside and outside of competitor and conspecific core use areas, and (b) at distance intervals in meters to the nearest competitor and conspecific. Collared individuals were from the Etosha National Park, Namibia (ENP), the Chobe National Park and Linyanti Conservancy, Botswana (CNP). (PDF) [file pone.0265054.s016.pdf]

S14 Table. Movement and activity metrics within competitor and conspecific core use areas. Nocturnal (30min fixes) and dusk/dawn (5min fixes) periods of lion and spotted hyena activity (AMVs), speed (m/s), and path tortuosity (radian) from (a) inside and outside of competitor and conspecific core use areas, and (b) at distance intervals in meters to the nearest competitor and conspecific. Collared individuals were from the Etosha National Park, Namibia (ENP), the Chobe National Park and Linyanti Conservancy, Botswana (CNP).

				Competitor						Conspecific					
				Activity		Speed (m/s)		Tortuosity		Activity		Speed (m/s)		Tortuosity	
				Lion	Hyena	Lion	Hyena	Lion	Hyena	Lion	Hyena	Lion	Hyena	Lion	Hyena
(a)	Nocturnal	ENP	Inside	36.60 ± 42.69	82.95 ± 61.26	0.183 ± 0.26	0.417 ± 0.39	0.055 ± 1.91	-0.016 ± 1.57	36.73 ± 40.62	93.43 ± 67.68	0.205 ± 0.25	0.435 ± 0.40	-0.056 ± 1.99	-0.079 ± 1.59
			Outside	34.41 ± 40.46	73.75 ± 68.48	0.186 ± 0.25	0.385 ± 0.41	0.059 ± 1.90	0.027 ± 1.80	35.78 ± 41.07	74.25 ± 66.84	0.192 ± 0.25	0.367 ± 0.41	0.138 ± 1.87	0.018 ± 1.74
		CNP	Inside	28.52 ± 39.35	78.76 ± 53.32	0.152 ± 0.22	0.438 ± 0.36	0.032 ± 2.41	0.049 ± 1.68	41.08 ± 32.77	43.99 ± 44.83	0.178 ± 0.21	0.233 ± 0.32	0.113 ± 2.88	0.131 ± 2.03
			Outside	27.55 ± 34.61	61.11 ± 56.42	0.141 ± 0.22	0.297 ± 0.36	-0.222 ± 2.43	0.041 ± 1.94	31.50 ± 36.45	74.89 ± 59.10	0.146 ± 0.23	0.334 ± 0.38	-0.123 ± 2.33	0.027 ± 1.80
	Dusk/dawn	ENP	Inside	37.89 ± 50.69	85.40 ± 68.64	0.217 ± 0.33	0.470 ± 0.49	0.073 ± 2.37	-0.015 ± 1.40	37.21 ± 47.90	95.73 ± 76.36	0.226 ± 0.32	0.478 ± 0.53	-0.369 ± 2.63	-0.048 ± 1.27
			Outside	33.80 ± 47.92	78.17 ± 75.01	0.204 ± 0.31	0.422 ± 0.52	0.029 ± 2.51	0.022 ± 1.65	34.45 ± 48.82	78.05 ± 73.44	0.200 ± 0.31	0.415 ± 0.51	0.088 ± 2.34	0.017 ± 1.62
		CNP	Inside	34.29 ± 50.88	84.32 ± 61.43	0.165 ± 0.28	0.527 ± 0.49	0.282 ± 1.79	0.002 ± 1.11	33.52 ± 41.75	50.97 ± 56.15	0.155 ± 0.25	0.268 ± 0.41	0.203 ± 1.39	0.091 ± 1.59
			Outside	30.66 ± 43.43	65.34 ± 64.85	0.157 ± 0.28	0.363 ± 0.47	0.263 ± 1.62	0.006 ± 1.38	37.16 ± 46.29	77.58 ± 67.21	0.173 ± 0.30	0.407 ± 0.50	0.097 ± 1.66	-0.004 ± 1.21
(b)	Nocturnal	ENP	0-100	22.33 ± 30.74	72.87 ± 41.51	0.100 ± 0.22	0.136 ± 0.17	-2.912 ± 1.66	-2.176 ± 1.56	26.26 ± 33.29	113.48 ± 26.97	0.127 ± 0.19	0.375 ± 0.38	0.169 ± 2.07	3.102 ± 1.52
			100-200	23.00 ± 30.16	79.20 ± 56.20	0.044 ± 0.11	0.124 ± 0.21	2.775 ± 1.58	-0.119 ± 2.20	53.78 40.41	127.18 ± 31.86	0.313 ± 0.26	0.179 ± 0.09	0.170 ± 1.12	1.254 ± 1.74
			200-300	29.52 45.83	81.61 ± 60.14	0.139 ± 0.25	0.291 ± 0.28	1.047 ± 1.65	-1.823 ± 1.38	41.75 ± 41.82	111.72 ± 102.84	0.245 ± 0.26	0.109 ± 0.10	-0.155 ± 1.52	-1.110 ± 1.23
			300-400	31.41 ± 35.05	70.41 ± 60.52	0.080 ± 0.15	0.266 ± 0.33	-2.159 ± 1.73	1.608 ± 1.70	34.01 ± 39.56	67.92 ± 59.42	0.212 ± 0.29	0.340 ± 0.56	0.014 ± 1.46	-0.412 ± 1.52
			400-500	18.67 ± 28.12	80.84 ± 49.37	0.059 ± 0.12	0.267 ± 0.24	2.305 ± 1.27	0.708 ± 1.38	51.92 ± 44.42	66.87 ± 1.00	0.289 ± 0.27	0.139 ± 0.13	-0.001 ± 1.21	1.268 ± 1.48
			500-600	40.03 ± 45.91	68.73 ± 77.49	0.094 ± 0.15	0.255 ± 0.25	0.055 ± 1.12	0.636 ± 1.56	26.49 ± 31.14	135.17 ± 35.09	0.105 ± 0.19	0.234 ± 0.30	0.897 ± 1.56	-0.660 ± 0.98
			600-700	31.88 ± 37.33	93.37 ± 49.59	0.151 ± 0.23	0.345 ± 0.28	0.626 ± 1.65	-0.773 ± 1.62	53.74 ± 47.67	178.92 ± 23.74	0.224 ± 0.28	0.680 ± 0.77	-0.011 ± 1.34	-1.060 ± 1.41
			700-800	36.89 ±	68.23 ±	0.135 ±	0.335 ±	0.401 ±	0.481 ±	48.83 ±	80.93 ±	0.226 ±	0.363 ±	0.293 ±	0.831 ±

			40.20	60.23	0.23	0.36	1.71	1.22	50.73	88.70	0.26	0.37	1.51	1.05
		**800-900**	29.80 ± 33.87	68.39 ± 65.28	0.185 ± 0.28	0.344 ± 0.34	1.147 ± 1.65	-0.020 ± 1.64	40.74 ± 38.65	-	0.271 ± 0.25	-	0.093 ± 1.57	2.407 ± 2.32
		**900-1000**	34.28 ± 42.46	80.51 ± 56.58	0.150 ± 0.23	0.292 ± 0.30	-0.223 ± 1.89	0.439 ± 1.73	35.72 ± 45.01	117.53 ± 92.47	0.168 ± 0.24	0.326 ± 0.43	1.761 ± 1.59	-0.008 ± 0.89
		**0-100**	9.71 ± 4.73	-	0.041 ± 0.03	0.088 ± 0.09	2.799 ± 1.58	-2.147 ± 1.30	-	72.09 ± 53.20	0.024 ± 0.09	0.120 ± 0.18	-2.162 ± 2.16	0.036 ± 2.12
		**100-200**	28.57 ± 36.33	116.35 ± 58.80	0.014 ± 0.02	0.407 ± 0.23	-2.134 ± 1.50	1.204 ± 1.43	46.86 ± 28.76	49.40 ± 41.43	0.128 ± 0.02	0.221 ± 0.32	-2.342 ± 1.71	-1.825 ± 2.57
		**200-300**	37.47 ± 48.78	75.89 ± 89.11	0.082 ± 0.20	0.288 ± 0.46	0.380 ± 1.25	0.323 ± 1.47	51.45 ± 48.20	39.26 ± 45.29	0.069 ± 0.05	0.114 ± 0.16	1.911 ± 1.45	1.526 ± 2.41
		**300-400**	14.20 ± 15.97	33.23 ± 33.74	0.022 ± 0.02	0.285 ± 0.60	-0.762 ± 1.46	-0.185 ± 1.41	11.42 ± 24.22	73.31 ± 56.89	0.101 ± 0.34	0.137 ± 0.19	-2.775 ± 2.46	1.033 ± 2.75
Nocturnal	CNP	**400-500**	37.89 ± 57.70	131.33 ± 56.82	0.128 ± 0.25	0.163 ± 0.19	1.460 ± 1.67	-0.080 ± 1.34	51.54 ± 42.14	53.33 ± 56.82	0.073 ± 0.07	0.108 ± 0.30	-0.739 ± 2.30	1.125 ± 2.15
		**500-600**	26.63 ± 47.72	66.80 ± 47.18	0.115 ± 0.25	0.535 ± 0.34	-1.710 ± 1.62	-0.887 ± 1.43	-	29.50 ± 23.29	0.057 ± 0.11	0.141 ± 0.24	-0.340 ± 2.08	-1.412 ± 1.80
		**600-700**	2.08 ± 2.92	127.08 ± 102.00	0.079 ± 0.11	0.267 ± 0.40	-0.844 ± 2.36	-0.259 ± 1.30	-	47.18 ± 52.83	0.031 ± 0.04	0.124 ± 0.19	2.567 ± 1.82	-1.847 ± 1.91
		**700-800**	12.64 ± 23.05	49.68 ± 57.51	0.041 ± 0.10	0.245 ± 0.29	-1.433 ± 2.15	-0.696 ± 1.71	-	63.15 ± 51.95	0.126 ± 0.19	0.267 ± 0.33	-0.078 ± 1.86	0.744 ± 1.94
		**800-900**	32.19 ± 58.03	71.89 ± 48.63	0.076 ± 0.19	0.282 ± 0.35	2.083 ± 2.08	0.826 ± 1.90	21.33 ± 35.93	41.41 ± 37.79	0.053 ± 0.13	0.111 ± 0.28	0.739 ± 1.60	-1.765 ± 1.72
		**900-1000**	19.45 ± 24.51	77.99 ± 35.51	0.207 ± 0.38	0.185 ± 0.17	0.120 ± 1.87	0.289 ± 1.61	-	28.13 ± 38.93	0.024 ± 0.04	0.127 ± 0.26	0.929 ± 1.76	2.164 ± 2.10
		**0-100**	32.26 ± 43.67	54.04 ± 48.55	0.063 ± 0.14	0.135 ± 0.15	-3.037 ± 2.03	-0.485 ± 2.02	26.84 ± 37.69	-	0.108 ± 0.21	0.010 ± 0.01	-1.533 ± 2.39	-0.658 ± 1.46
		**100-200**	23.32 ± 50.22	44.29 ± 57.19	0.082 ± 0.24	0.171 ± 0.22	3.130 ± 1.73	1.038 ± 1.87	53.66 ± 49.73	-	0.412 ± 0.34	0.139 ± 0.01	0.076 ± 1.14	-0.754 ± 1.17
		**200-300**	35.13 ± 52.09	88.49 ± 73.61	0.155 ± 0.26	0.318 ± 0.30	1.620 ± 1.79	0.002 ± 1.64	46.28 ± 50.56	137.00 ± 110.31	0.337 ± 0.34	0.239 ± 0.17	0.145 ± 1.43	0.350 ± 0.94
		**300-400**	31.35 ± 42.61	68.58 ± 64.74	0.085 ± 0.21	0.294 ± 0.42	-2.571 ± 1.94	-0.647 ± 1.72	50.00 ± 51.91	164.00 ± 128.69	0.302 ± 0.39	0.508 ± 0.11	0.102 ± 1.92	-0.028 ± 0.82
Dusk/dawn	ENP	**400-500**	33.79 ± 44.56	81.96 ± 59.40	0.135 ± 0.26	0.282 ± 0.29	-2.666 ± 1.88	0.047 ± 1.50	79.47 ± 47.28	-	0.396 ± 0.37	0.070 ± 0.06	0.058 ± 1.48	-0.264 ± 0.93
		**500-600**	20.41 ± 38.26	84.33 ± 71.13	0.188 ± 0.33	0.406 ± 0.36	2.414 ± 1.77	0.280 ± 1.38	35.86 ± 46.90	104.56 ± 94.04	0.184 ± 0.34	0.328 ± 0.43	-0.131 ± 2.03	0.571 ± 1.10
		**600-700**	42.27 ± 59.37	61.78 ± 69.45	0.257 ± 0.36	0.390 ± 0.50	0.906 ± 1.37	-0.116 ± 1.61	50.93 ± 58.36	149.38 ± 106.21	0.254 ± 0.43	0.506 ± 0.53	0.624 ± 1.71	-0.522 ± 0.68
		**700-800**	27.42 ± 41.12	57.03 ± 66.58	0.162 ± 0.33	0.339 ± 0.44	-2.842 ± 1.70	0.105 ± 2.02	49.20 ± 57.70	-	0.319 ± 0.40	0.168 ± 0.06	-0.057 ± 1.57	0.265 ± 0.68
		**800-900**	46.56 ± 56.70	99.41 ± 77.77	0.160 ± 0.27	0.405 ± 0.43	-0.322 ± 1.65	0.188 ± 1.27	70.72 ± 58.96	-	0.325 ± 0.40	0.080 ± 0.16	-0.351 ± 1.47	0.971 ± 0.96

		**900-1000**	43.79 ± 49.08	69.08 ± 49.65	0.154 ± 0.28	0.319 ± 0.37	-1.089 ± 2.11	0.171 ± 1.62	56.68 ± 50.48	-	0.257 ± 0.35	0.102 ± 0.02	-0.440 ± 1.86	0.090 ± 0.79
Dusk/dawn	CNP	**0-100**	79.60 ± 26.02	57.60 ± 56.21	0.163 ± 0.03	0.150 ± 0.25	-0.878 ± 2.18	-0.290 ± 1.30	76.71 ± 34.34	69.85 ± 71.64	0.048 ± 0.15	0.157 ± 0.39	0.053 ± 1.32	-0.330 ± 1.74
		**100-200**	16.46 ± 28.87	34.03 ± 50.63	0.071 ± 0.15	0.312 ± 0.49	0.973 ± 1.47	-1.014 ± 1.32	123.84 ± 35.98	63.42 ± 60.98	0.118 ± 0.22	0.209 ± 0.30	0.401 ± 1.18	-0.143 ± 1.49
		**200-300**	39.34 ± 33.27	92.50 ± 20.07	0.121 ± 0.22	0.290 ± 0.25	-0.262 ± 1.35	-0.132 ± 0.91	70.69 ± 57.12	53.06 ± 47.97	0.079 ± 0.15	0.175 ± 0.34	-0.687 ± 1.32	-1.362 ± 1.74
		**300-400**	13.44 ± 43.89	57.25 ± 80.96	0.133 ± 0.43	0.340 ± 0.67	-0.070 ± 1.09	0.141 ± 1.14	90.79 ± 48.49	58.01 ± 59.28	0.117 ± 0.19	0.169 ± 0.27	-0.402 ± 1.19	-0.104 ± 1.31
		**400-500**	10.83 ± 39.96	157.00 ± 138.59	0.103 ± 0.18	0.272 ± 0.40	-2.849 ± 1.64	0.112 ± 0.96	125.31 ± 51.26	44.76 ± 51.58	0.169 ± 0.20	0.125 ± 0.23	-1.092 ± 1.09	-0.170 ± 1.42
		**500-600**	29.50 ± 0.10	90.50 ± 26.87	0.064 ± 0.01	0.521 ± 0.07	-0.280 ± 1.06	-0.230 ± 0.83	45.55 ± 52.24	54.46 ± 52.76	0.073 ± 0.21	0.146 ± 0.26	-0.137 ± 1.10	0.083 ± 1.53
		**600-700**	22.89 37.56	106.17 ± 27.62	0.054 ± 0.14	0.598 ± 0.28	0.124 ± 1.43	0.124 ± 0.69	22.16 ± 37.07	59.17 ± 56.41	0.075 ± 0.20	0.149 ± 0.27	-0.229 ± 1.21	0.057 ± 1.43
		**700-800**	31.77 ± 42.21	73.66 ± 52.78	0.150 ± 0.36	0.377 ± 0.35	-0.250 ± 1.91	-0.188 ± 1.12	56.44 ± 49.32	46.21 ± 53.27	0.114 ± 0.18	0.187 ± 0.31	0.307 ± 1.12	-1.366 ± 1.69
		**800-900**	18.41 ± 10.20	83.09 ± 43.51	0.032 ± 0.05	0.321 ± 0.38	-0.579 ± 1.34	0.221 ± 1.22	46.08 ± 33.59	56.63 ± 58.35	0.080 ± 0.19	0.193 ± 0.31	-0.222 ± 1.25	0.273 ± 1.40
		**900-1000**	15.39 43.24	90.68 ± 34.54	0.234 ± 0.50	0.266 ± 0.43	1.541 ± 1.87	-0.042 ± 1.24	86.99 ± 41.31	61.38 ± 58.80	0.115 ± 0.19	0.233 ± 0.31	-0.474 ± 1.33	-0.313 ± 1.24
