# Supplementary material for: Coursing hyenas and stalking lions: The potential for inter- and intraspecific interactions
Source: PLoS One. 2023 Feb 3;18(2):e0265054. doi: 10.1371/journal.pone.0265054 (PMC9897591; doi:10.1371/journal.pone.0265054)
Supplement: S1 Fig — Individuals of the Etosha National Park, Namibia (left) are separated from individuals of the Chobe National Park, Linyanti Conservancy, and Okavango Delta, Botswana (right) at the bold line indicated on 6 April 2015. Males are denoted with an asterisk. (PDF) [file pone.0265054.s017.pdf]

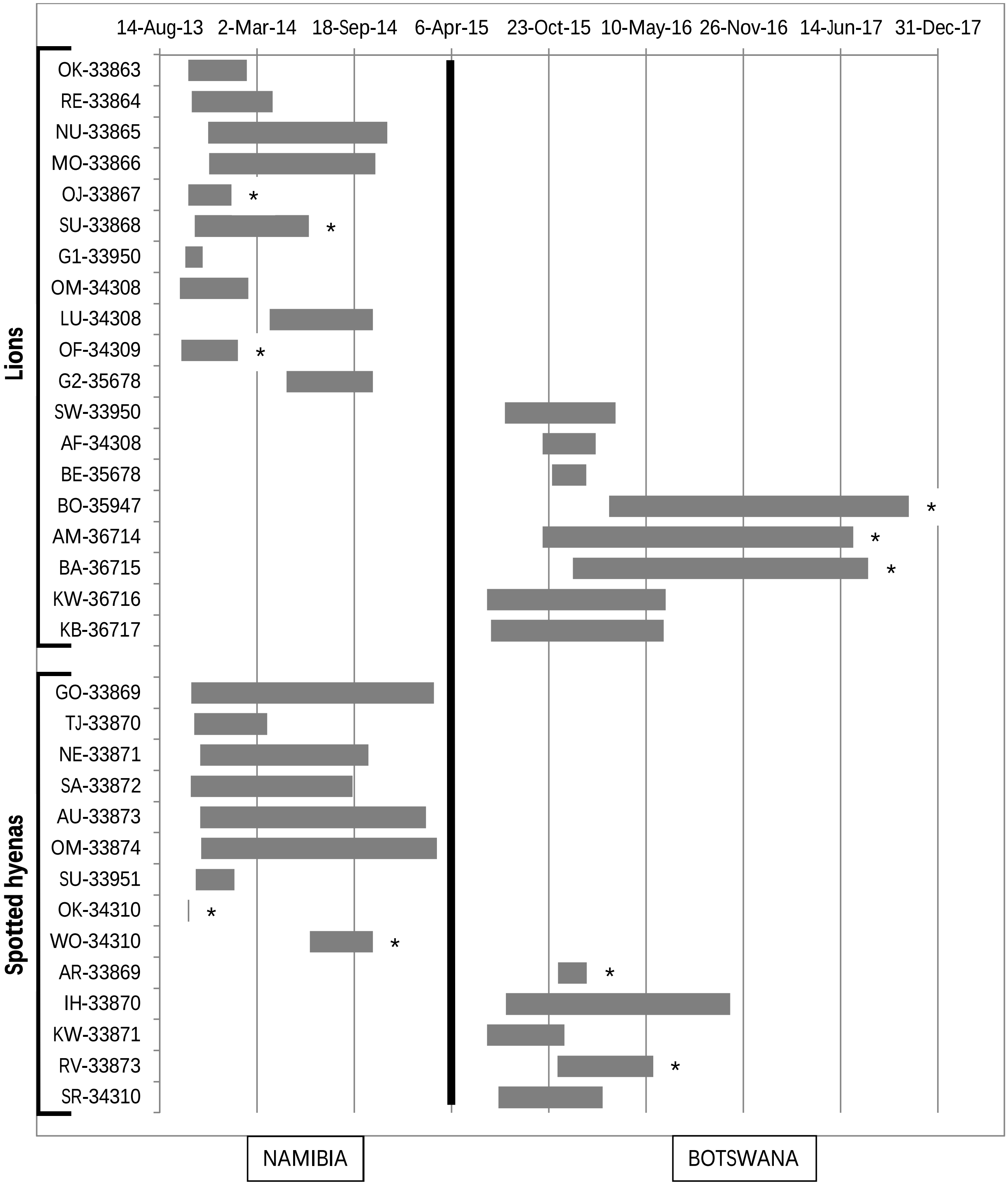

S1 Fig. Temporal schedule of collar overlap for lions and spotted hyenas. Individuals of the Etosha National Park, Namibia (left) are separated from individuals of the Chobe National Park, Linyanti Conservancy, and Okavango Delta, Botswana (right) at the bold line indicated on 6 April 2015. Males are denoted with an asterisk.
