# Supplementary material for: Coursing hyenas and stalking lions: The potential for inter- and intraspecific interactions
Source: PLoS One. 2023 Feb 3;18(2):e0265054. doi: 10.1371/journal.pone.0265054 (PMC9897591; doi:10.1371/journal.pone.0265054)
Supplement: S2 Fig — Utilization distributions were constructed with the KDE (far left and second from right) and LoCoH a-method (second from left and far right). Panels represent the 95% isopleth of the individual’s home range for the dry season (left two panels) and wet season (right two panels). Unique identifiers are depicted vertically on the left of each row of maps. Maps indicate the individual’s home range on a satellite image of the (a) Etosha National Park with the salt pan visible; (b) Chobe National Park with the Chobe river from west to east; (c) Linyanti Conservancy with the Linyanti river from southwest to northeast; and the (d) NG32 concession in the Okavango Delta on the southwestern tip of Chief’s Island. Map source: Google Imagery, TerraMetrics. (PDF) [file pone.0265054.s018.pdf]

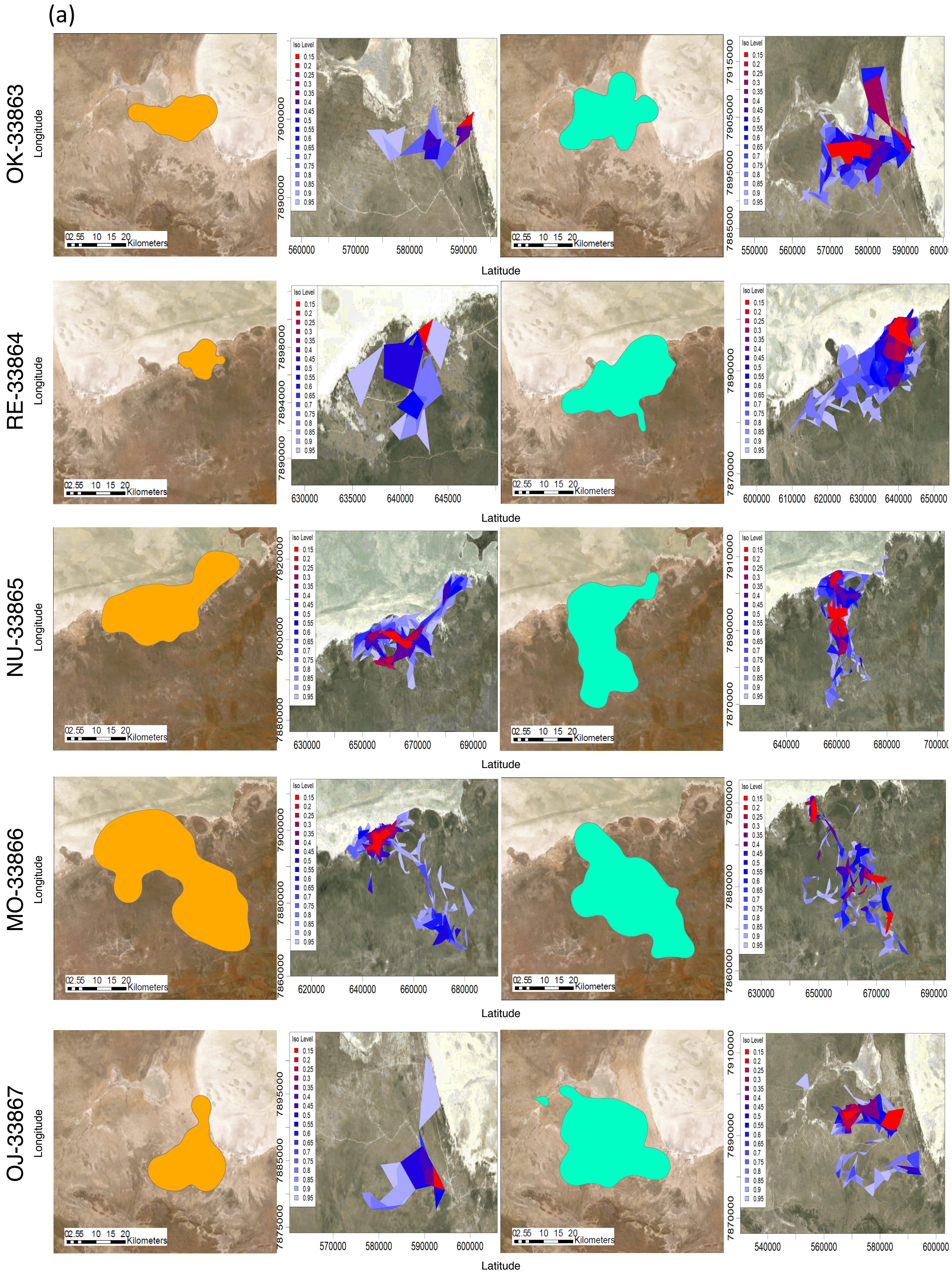

(a)
OK-33863
RE-33864
NU-33865
MO-33866
OJ-33867
Longitude
Latitude
Iso Level
0.15
0.2
0.25
0.3
0.35
0.4
0.45
0.5
0.55
0.6
0.65
0.7
0.75
0.8
0.85
0.9
0.95
02.55 10 15 20
Kilometers

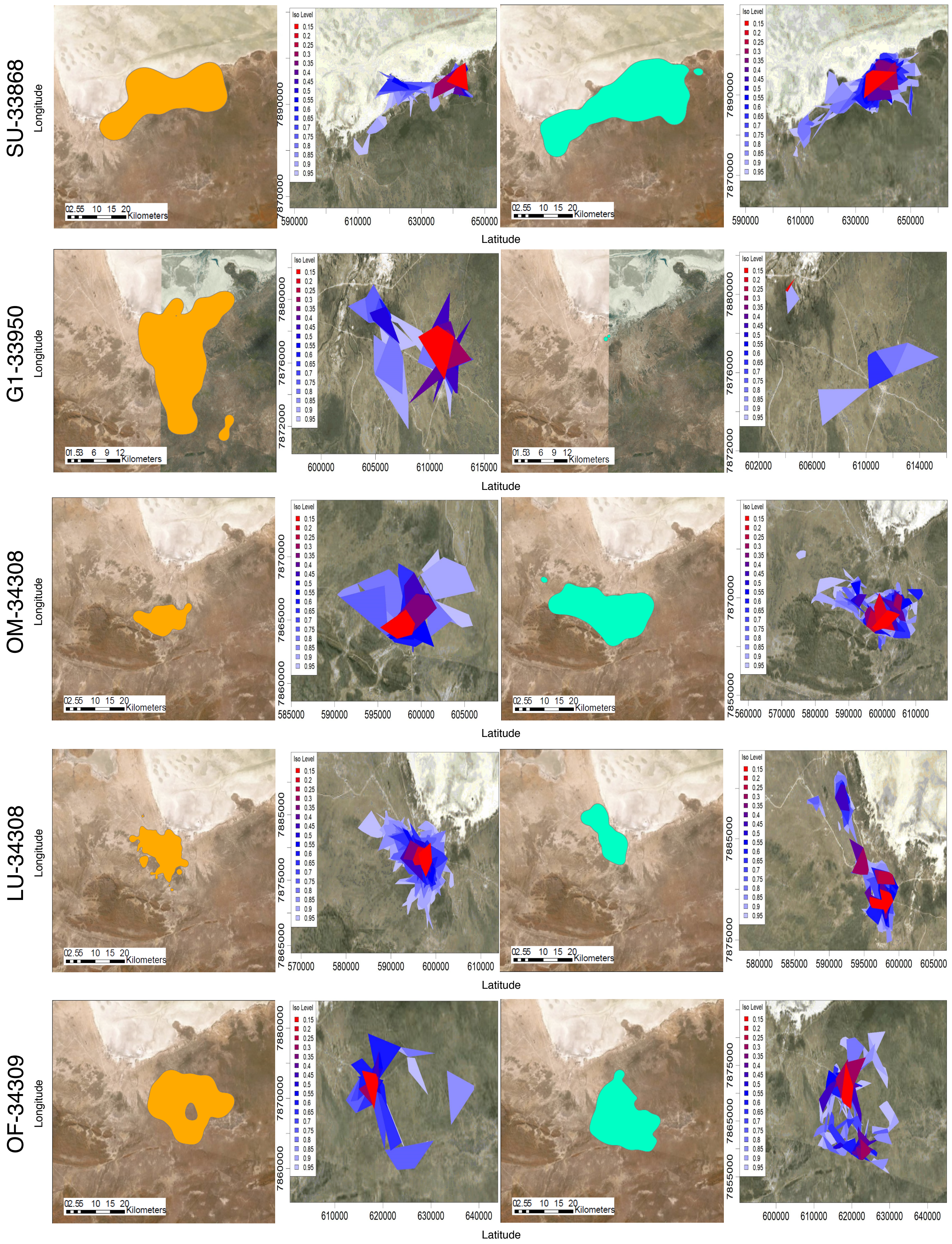
SU-33868
G1-33950
OM-34308
LU-34308
OF-34309
Longitude
Latitude
Iso Level
0.15
0.2
0.25
0.3
0.35
0.4
0.45
0.5
0.55
0.6
0.65
0.7
0.75
0.8
0.85
0.9
0.95
Kilometers
02.55 10 15 20
01.53 6 9 12
590000 610000 630000 650000
7870000 7890000
600000 605000 610000 615000
7872000 7876000 7880000
602000 606000 610000 614000
585000 590000 595000 600000 605000
7860000 7865000 7870000
560000 570000 580000 590000 600000 610000
7850000 7870000
570000 580000 590000 600000 610000
7865000 7875000 7885000
580000 585000 590000 595000 600000 605000
7875000 7885000
610000 620000 630000 640000
7860000 7870000 7880000
600000 610000 620000 630000 640000
7855000 7865000 7875000

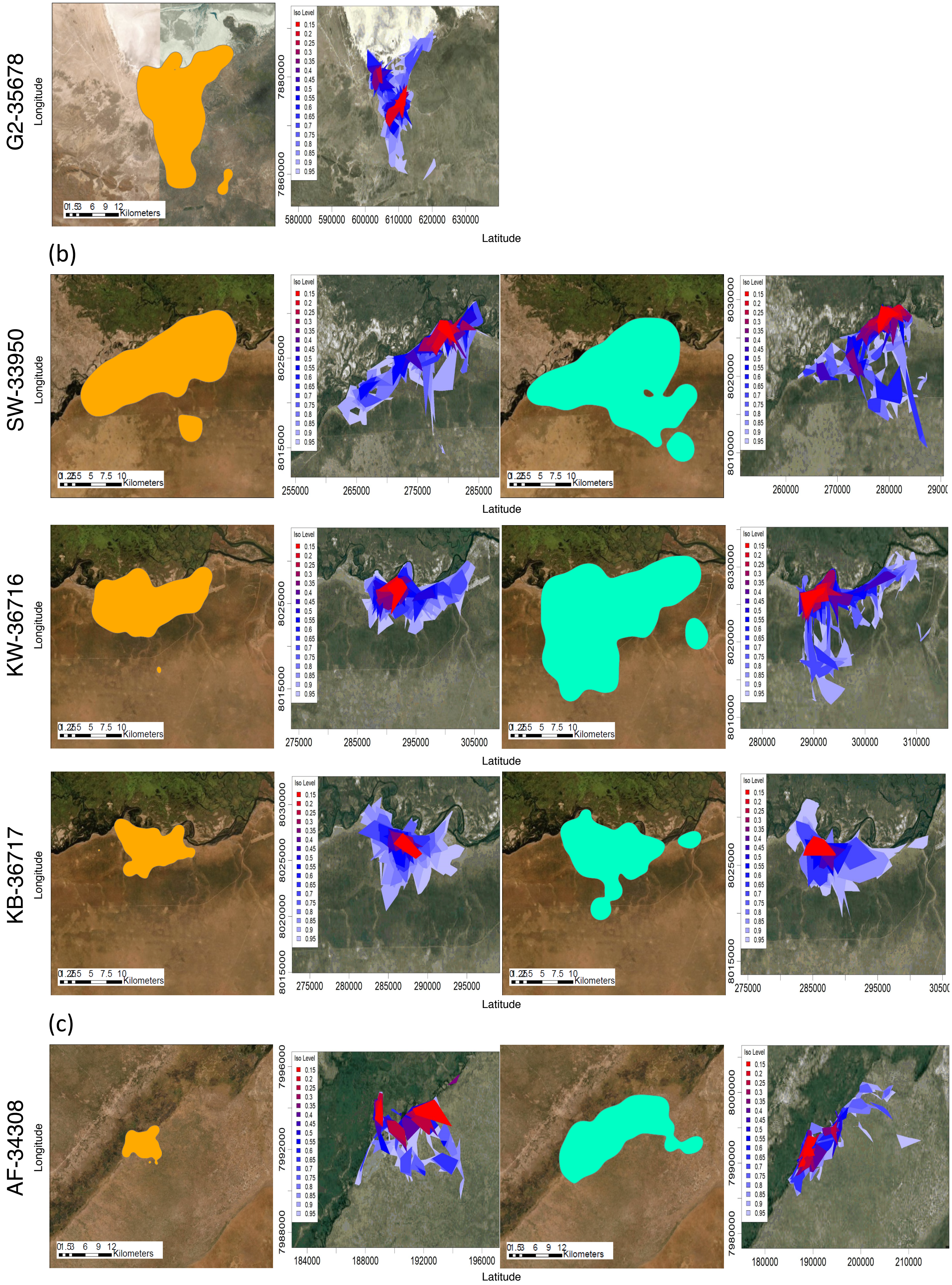
G2-35678
Longitude
Iso Level
Kilometers
Latitude
(b)
SW-33950
KW-36716
KB-36717
(c)
AF-34308

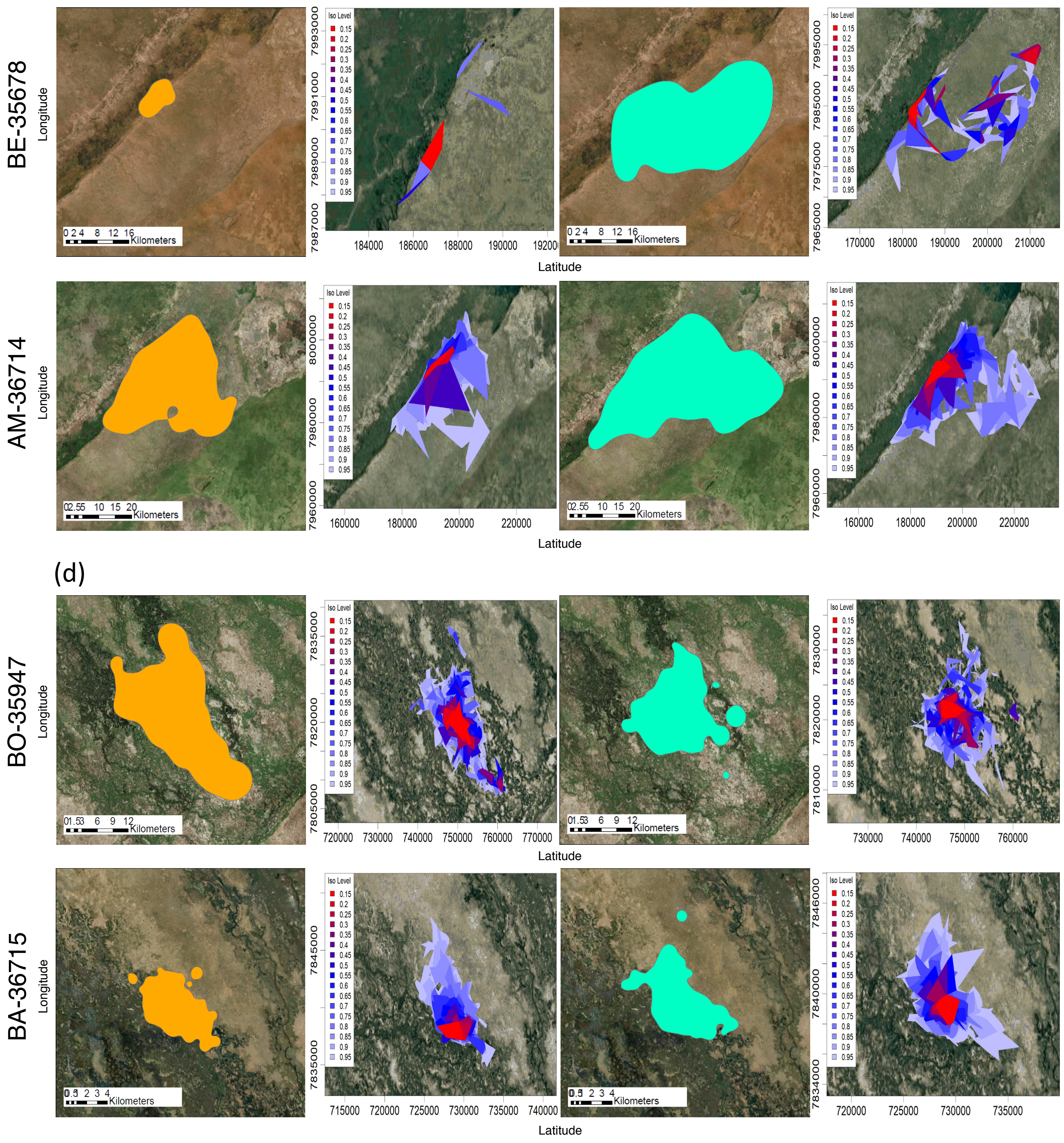

S2 Fig. Lion nocturnal space use. Utilization distributions were constructed with the KDE (far left and second from right) and LoCoH a-method (second from left and far right). Panels represent the 95% isopleth of the individual's home range for the dry season (left two panels) and wet season (right two panels). Unique identifiers are depicted vertically on the left of each row of maps. Maps indicate the individual's home range on a satellite image of the (a) Etosha National Park with the salt pan visible; (b) Chobe National Park with the Chobe river from west to east; (c) Linyanti Conservancy with the Linyanti river from southwest to northeast; and the (d) NG32 concession in the Okavango Delta on the southwestern tip of Chief's Island. Map source: Google Imagery, TerraMetrics.
