# Supplementary material for: Coursing hyenas and stalking lions: The potential for inter- and intraspecific interactions
Source: PLoS One. 2023 Feb 3;18(2):e0265054. doi: 10.1371/journal.pone.0265054 (PMC9897591; doi:10.1371/journal.pone.0265054)
Supplement: S3 Fig — Utilization distributions were constructed with the KDE (far left and second from right) and LoCoH a-method (second from left and far right). Panels represent the 95% isopleth of the individual’s home range for the dry season (left two panels) and wet season (right two panels). Unique identifiers are depicted vertically on the left of each row of maps. Maps indicate the individual’s home range on a satellite image of the (a) Etosha National Park with the salt pan visible; (b) Chobe National Park with the Chobe river from west to east; (c) Linyanti Conservancy with the Linyanti river from southwest to northeast. Map source: Google Imagery, TerraMetrics. (PDF) [file pone.0265054.s019.pdf]

(a)

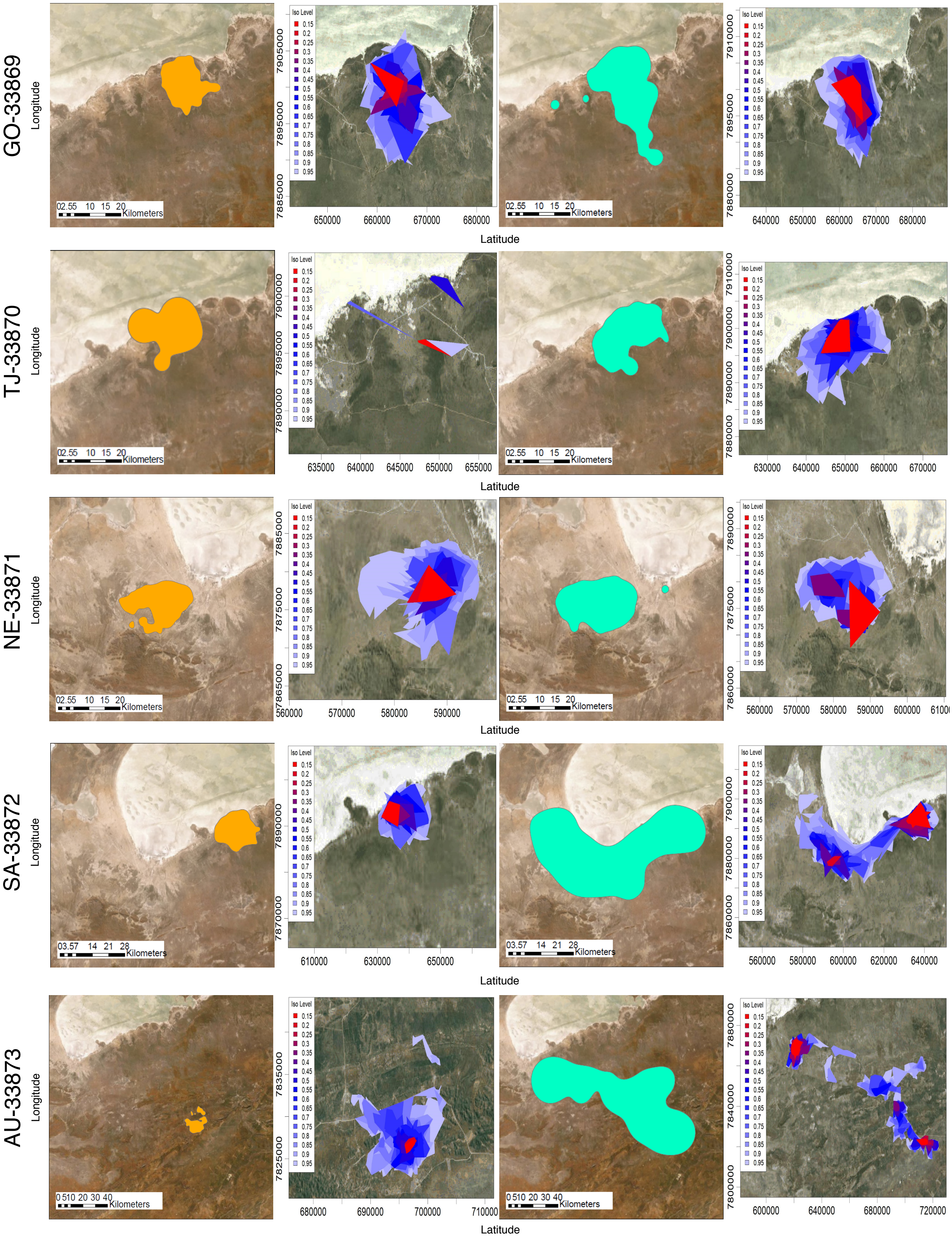
GO-33869
TJ-33870
NE-33871
SA-33872
AU-33873
Longitude
Latitude
Iso Level
0.15
0.2
0.25
0.3
0.35
0.4
0.45
0.5
0.55
0.6
0.65
0.7
0.75
0.8
0.85
0.9
0.95
Kilometers

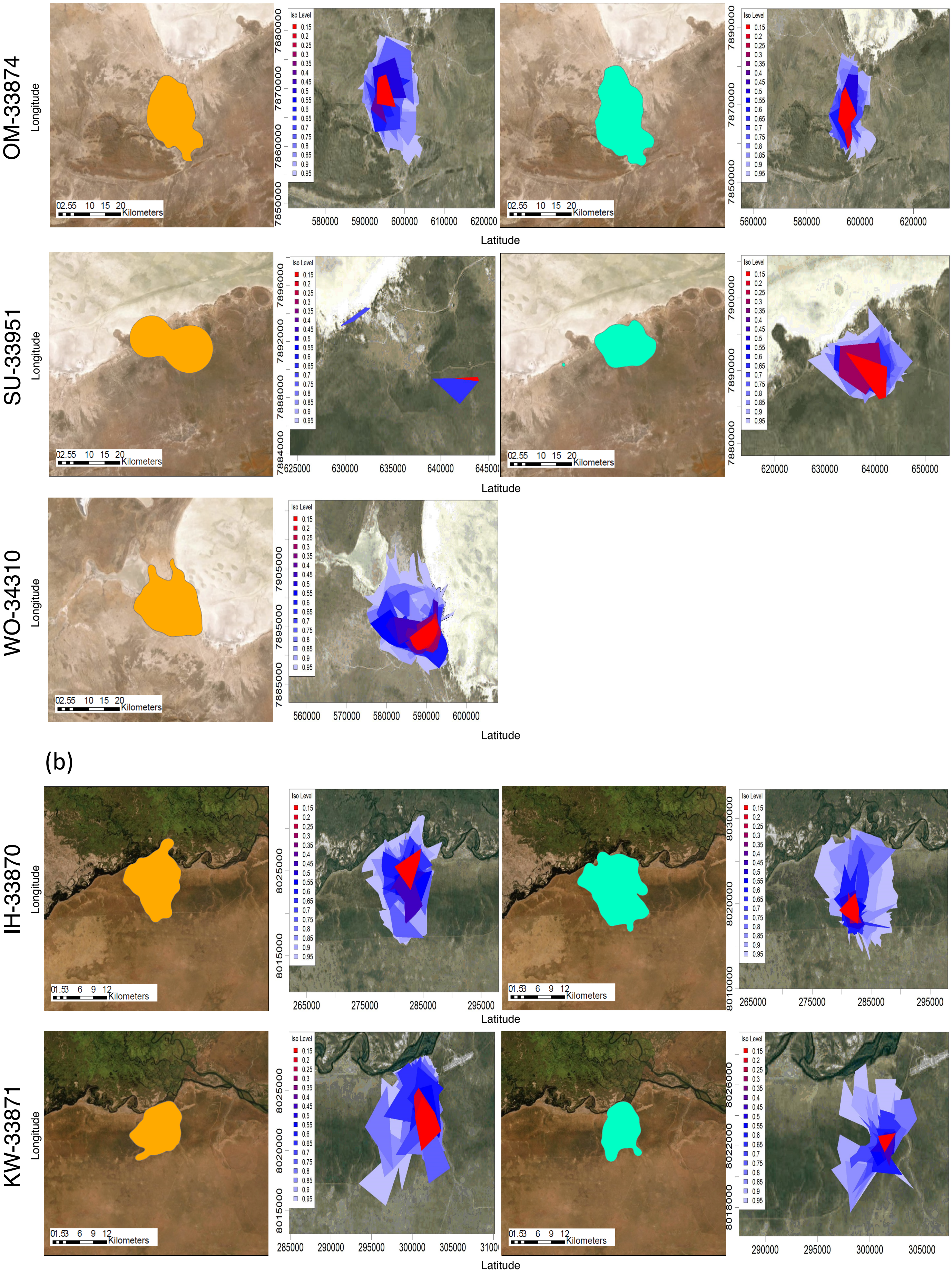
OM-33874
SU-33951
WO-34310
(b)
IH-33870
KW-33871
Longitude
Latitude
Iso Level
0.15
0.2
0.25
0.3
0.35
0.4
0.45
0.5
0.55
0.6
0.65
0.7
0.75
0.8
0.85
0.9
0.95
02.55 10 15 20 Kilometers
01.53 6 9 12 Kilometers
580000 590000 600000 610000 620000
7850000 7860000 7870000 7880000
560000 580000 600000 620000
7850000 7870000 7890000
625000 630000 635000 640000 645000
7884000 7888000 7892000 7896000
620000 630000 640000 650000
7880000 7890000 7900000
560000 570000 580000 590000 600000
7885000 7895000 7905000
265000 275000 285000 295000
8015000 8025000
8010000 8020000 8030000
285000 290000 295000 300000 305000
8015000 8020000 8025000
290000 295000 300000 305000
8018000 8022000 8026000

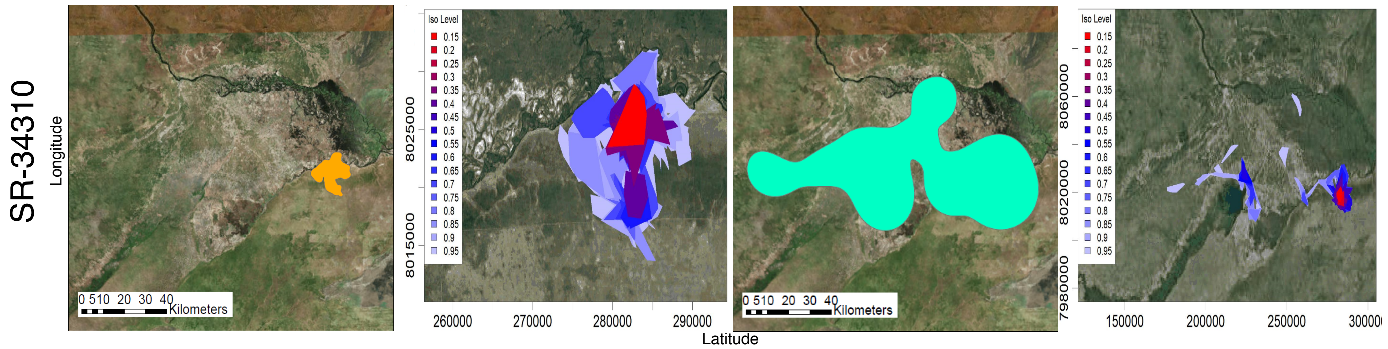

(c)

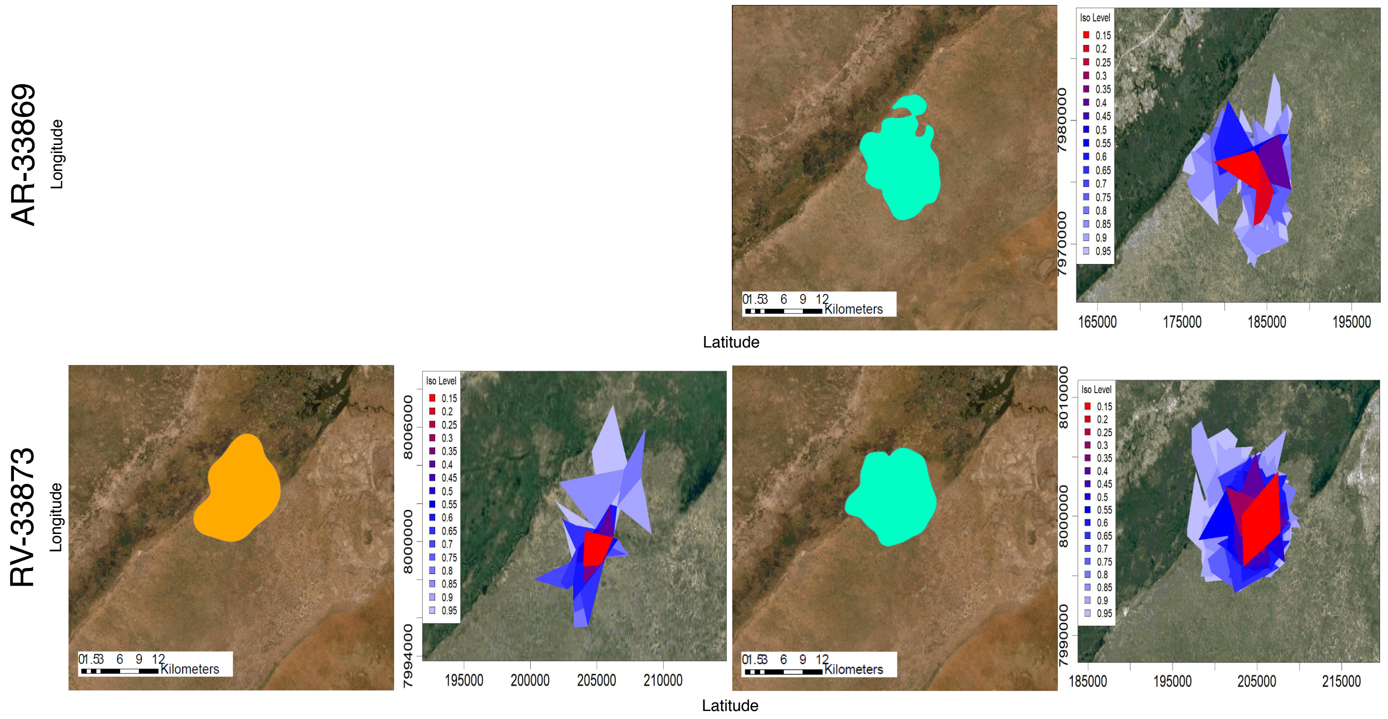

S3 Fig. Spotted hyena nocturnal space use. Utilization distributions were constructed with the KDE (far left and second from right) and LoCoH a-method (second from left and far right). Panels represent the 95% isopleth of the individual's home range for the dry season (left two panels) and wet season (right two panels). Unique identifiers are depicted vertically on the left of each row of maps. Maps indicate the individual's home range on a satellite image of the (a) Etosha National Park with the salt pan visible; (b) Chobe National Park with the Chobe river from west to east; and (c) Linyanti Conservancy with the Linyanti river from southwest to northeast. Map source: Google Imagery, TerraMetrics.
