# Supplementary material for: Coursing hyenas and stalking lions: The potential for inter- and intraspecific interactions
Source: PLoS One. 2023 Feb 3;18(2):e0265054. doi: 10.1371/journal.pone.0265054 (PMC9897591; doi:10.1371/journal.pone.0265054)
Supplement: S4 Fig — Both figures, males upper panels and females lower panels. Dry season = red lines, and wet season = blue lines. Solid lines represent the mean, and shaded bars are 95% CI. †No spotted hyenas were collared from the Okavango Delta, Botswana. (PDF) [file pone.0265054.s020.pdf]

(a)

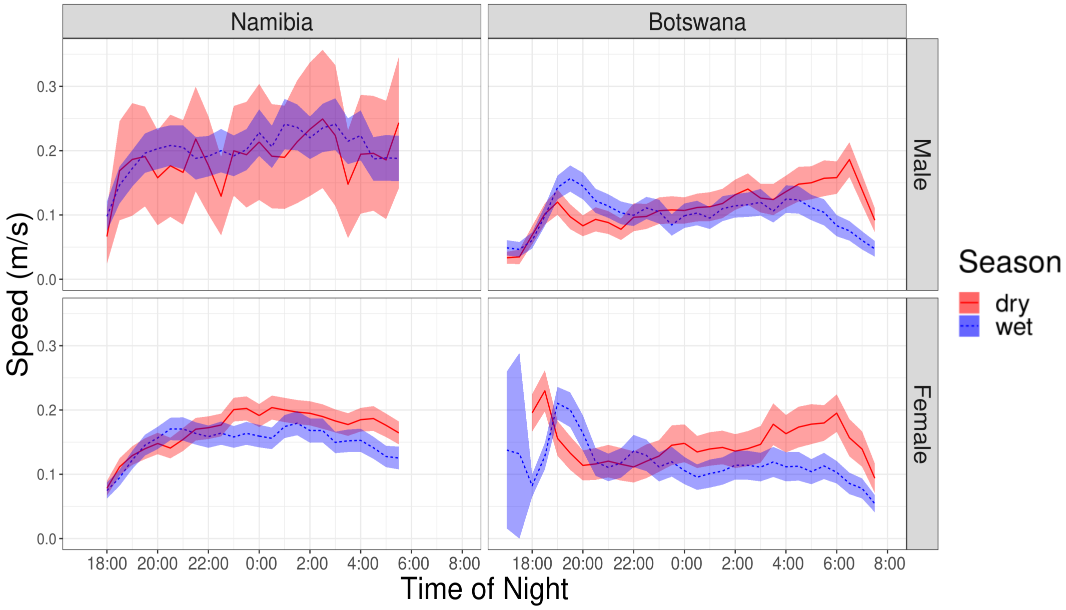

(b)

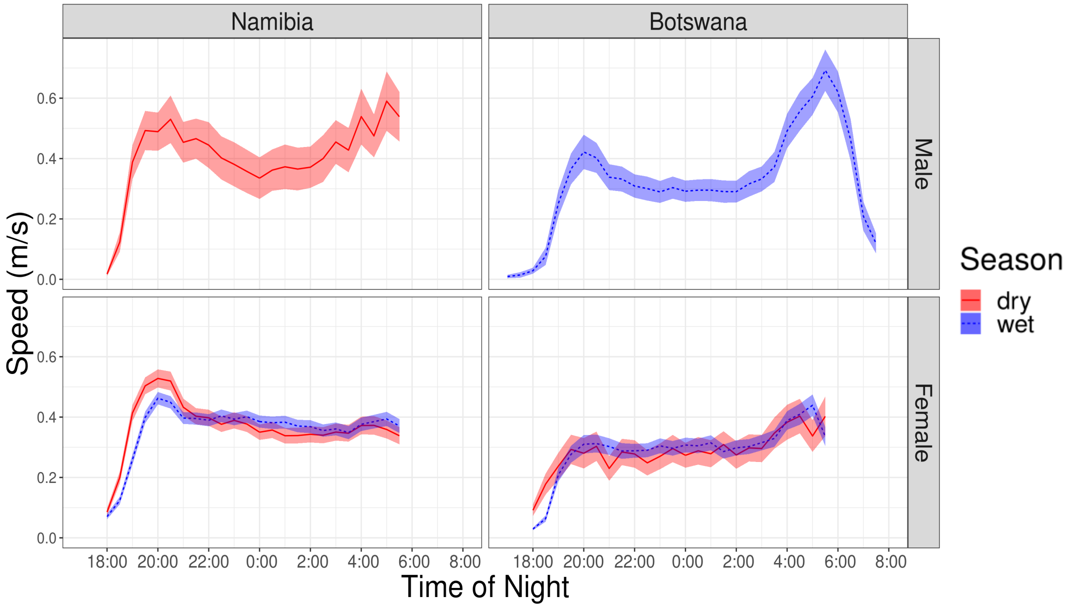

S4 Fig. Seasonal average speed (m/s) of (a) lions and (b) spotted hyenas from the Etosha National Park, Namibia (left panels) and the Chobe National Park, Linyanti Conservancy, and Okavango Delta†, Botswana (right panels) during nocturnal periods. Both figures, males upper panels and females lower panels. Dry season = red lines, and wet season = blue lines. Solid lines represent the mean, and shaded bars are 95% CI.

†*No spotted hyenas were collared from the Okavango Delta, Botswana.*
