# Supplementary material for: Coursing hyenas and stalking lions: The potential for inter- and intraspecific interactions
Source: PLoS One. 2023 Feb 3;18(2):e0265054. doi: 10.1371/journal.pone.0265054 (PMC9897591; doi:10.1371/journal.pone.0265054)
Supplement: S5 Fig — Violins depict the probability distribution, with black dots the mean and black lines the 95% confidence intervals. †No spotted hyenas were collared from the Okavango Delta, Botswana. (PDF) [file pone.0265054.s021.pdf]

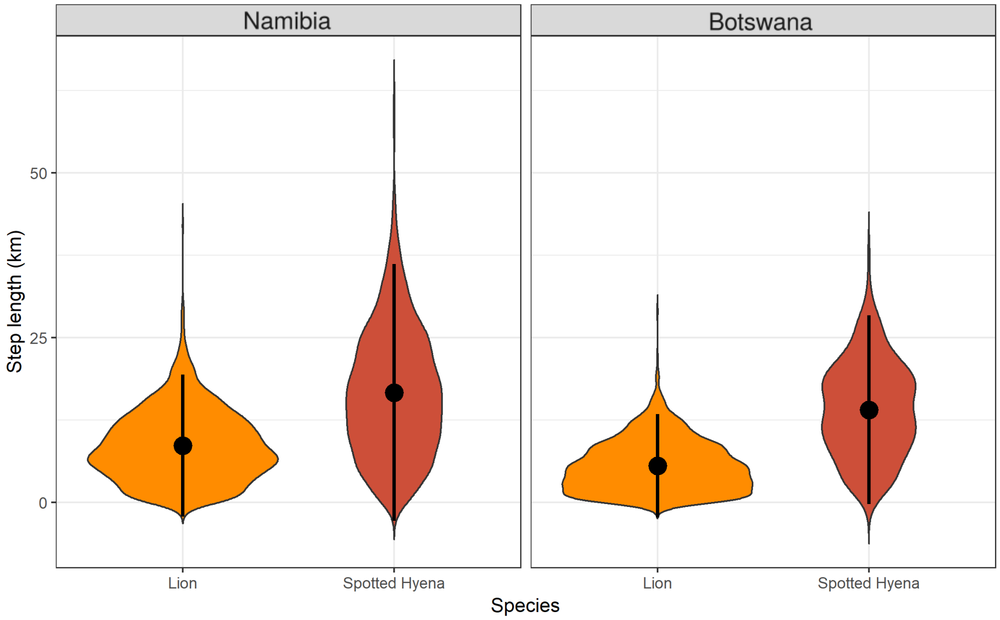

S5 Fig. Step lengths of lions and spotted hyenas from the Etosha National Park, Namibia (left panel) and the Chobe National Park, Linyanti Conservancy, and Okavango Delta†, Botswana (right panel). Violins depict the probability distribution, with black dots the mean and black lines the 95% confidence intervals.

†*No spotted hyenas were collared from the Okavango Delta, Botswana.*
