# Supplementary material for: Coursing hyenas and stalking lions: The potential for inter- and intraspecific interactions
Source: PLoS One. 2023 Feb 3;18(2):e0265054. doi: 10.1371/journal.pone.0265054 (PMC9897591; doi:10.1371/journal.pone.0265054)
Supplement: S7 Fig — (a) 24 hour cycle binned into six 4 hour periods. (b) Nocturnal cycle of 17h00-8h00 binned into hourly periods. Bars represent the mean and error bars the SE, only upper error bars are shown. Lion individuals (n = 8) represented by pink/purple/blue/blue-green colors. Spotted hyena individuals (n = 5) represented by green/yellow/orange colors. †No spotted hyenas were collared from the Okavango Delta, Botswana. (PDF) [file pone.0265054.s023.pdf]

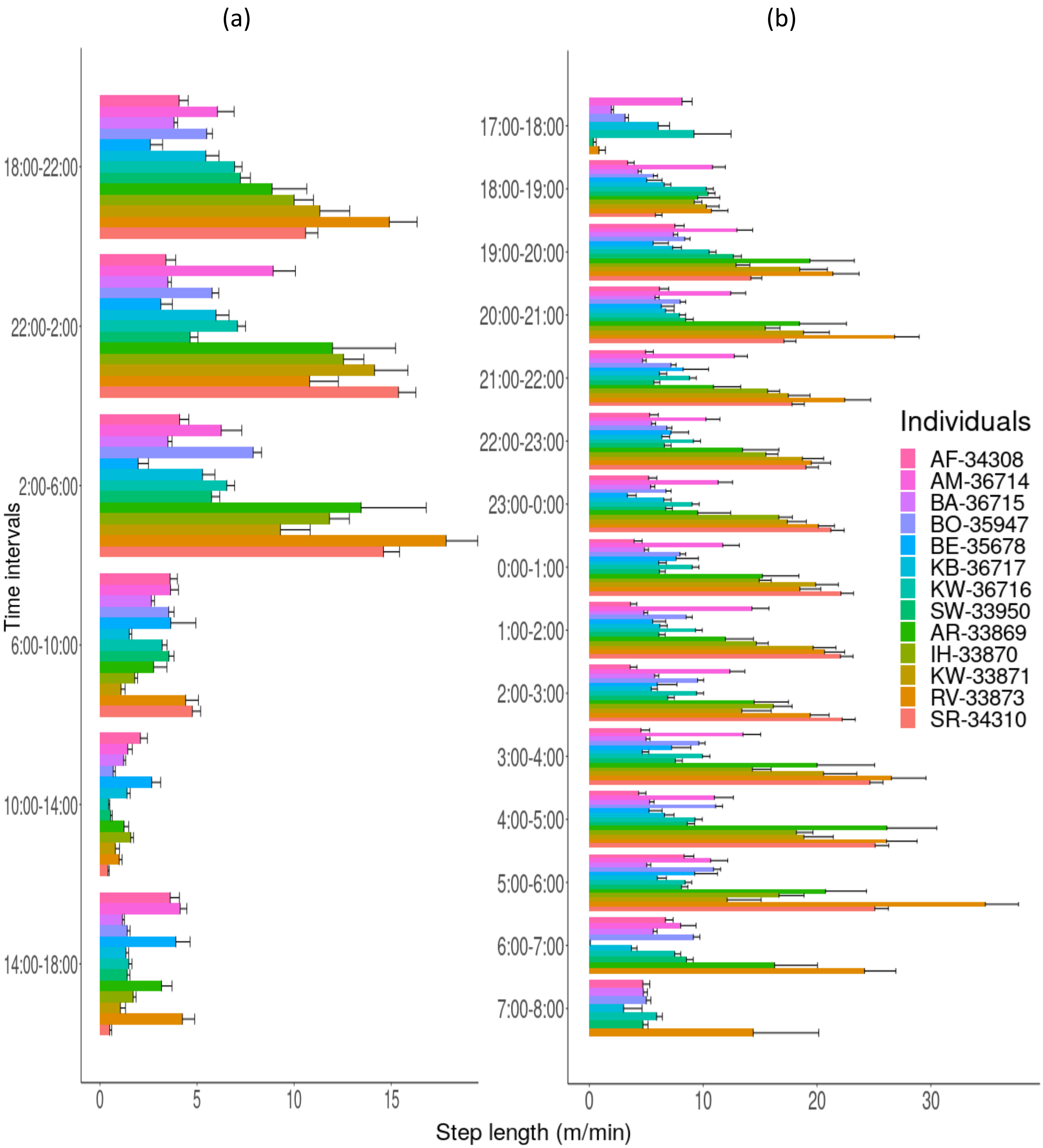

S7 Fig. Mean step length (m/min) of lions and spotted hyena individuals from the Chobe National Park, Linyanti Conservancy, and Okavango Delta†, Botswana. (a) 24-hour cycle binned into six 4-hour periods. (b) Nocturnal cycle of 17h00-8h00 binned into hourly periods. Bars represent the mean and error bars the SE, only upper error bars are shown. Lion individuals (n = 8) represented by pink/purple/blue/blue-green colours. Spotted hyena individuals (n = 5) represented by green/yellow/orange colours.

†*No spotted hyenas were collared from the Okavango Delta, Botswana.*
