# Supplementary material for: Coursing hyenas and stalking lions: The potential for inter- and intraspecific interactions
Source: PLoS One. 2023 Feb 3;18(2):e0265054. doi: 10.1371/journal.pone.0265054 (PMC9897591; doi:10.1371/journal.pone.0265054)
Supplement: S8 Fig — (PDF) [file pone.0265054.s024.pdf]

(a)

(b)

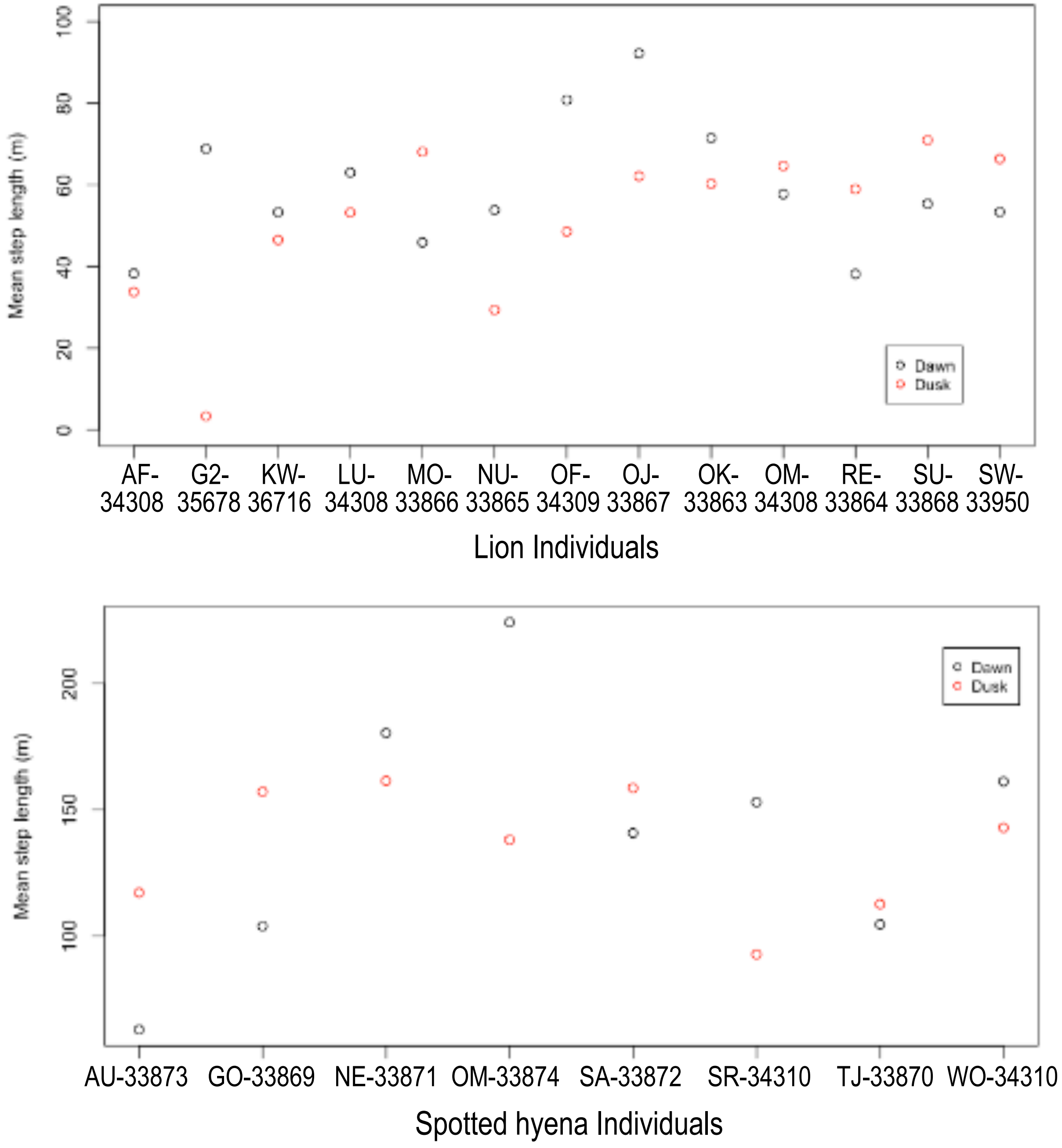

S8 Fig. Mean step length at dusk (red circles) and dawn (black circles) for lions (a) and spotted hyenas (b) from the Etosha National Park, Namibia and the Chobe National Park and Linyanti Conservancy, Botswana.
