# Supplementary material for: Coursing hyenas and stalking lions: The potential for inter- and intraspecific interactions
Source: PLoS One. 2023 Feb 3;18(2):e0265054. doi: 10.1371/journal.pone.0265054 (PMC9897591; doi:10.1371/journal.pone.0265054)
Supplement: S9 Fig — Males dark grey boxes, females white boxes. Boxplots show medians, 25% and 75% quartiles. Dashed lines indicate means. Whiskers indicate the IQR range. †No spotted hyenas were collared from the Okavango Delta, Botswana. (PDF) [file pone.0265054.s025.pdf]

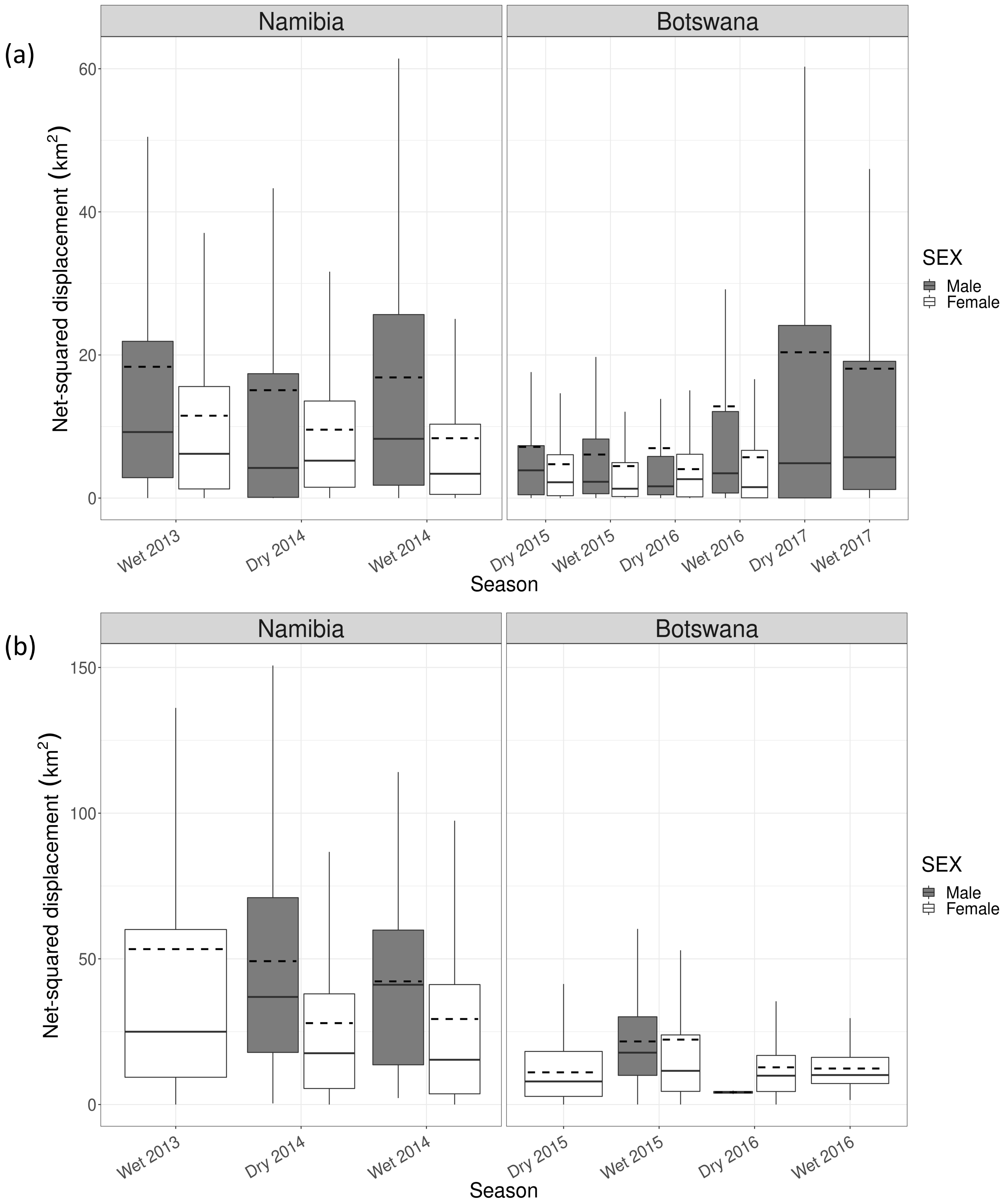

S9 Fig. Seasonal mean net-squared displacement (km^2) over 24-hour cycles for (a) lions and (b) spotted hyenas across each season from the Etosha National Park, Namibia (left panels) and the Chobe National Park, Linyanti Conservancy, and Okavango Delta†, Botswana (right panels). Males dark grey boxes, females white boxes. Boxplots show medians, 25% and 75% quartiles. Dashed lines indicate means. Whiskers indicate the IQR range.

†*No spotted hyenas were collared from the Okavango Delta, Botswana.*
