# Supplementary material for: Coursing hyenas and stalking lions: The potential for inter- and intraspecific interactions
Source: PLoS One. 2023 Feb 3;18(2):e0265054. doi: 10.1371/journal.pone.0265054 (PMC9897591; doi:10.1371/journal.pone.0265054)
Supplement: S10 Fig — Both figures, males top panels and females bottom panels. Boxplots show medians, 25% and 75% quartiles. Dashed lines indicate means. Whiskers indicate the IQR range. †No spotted hyenas were collared from the Okavango Delta, Botswana. (PDF) [file pone.0265054.s026.pdf]

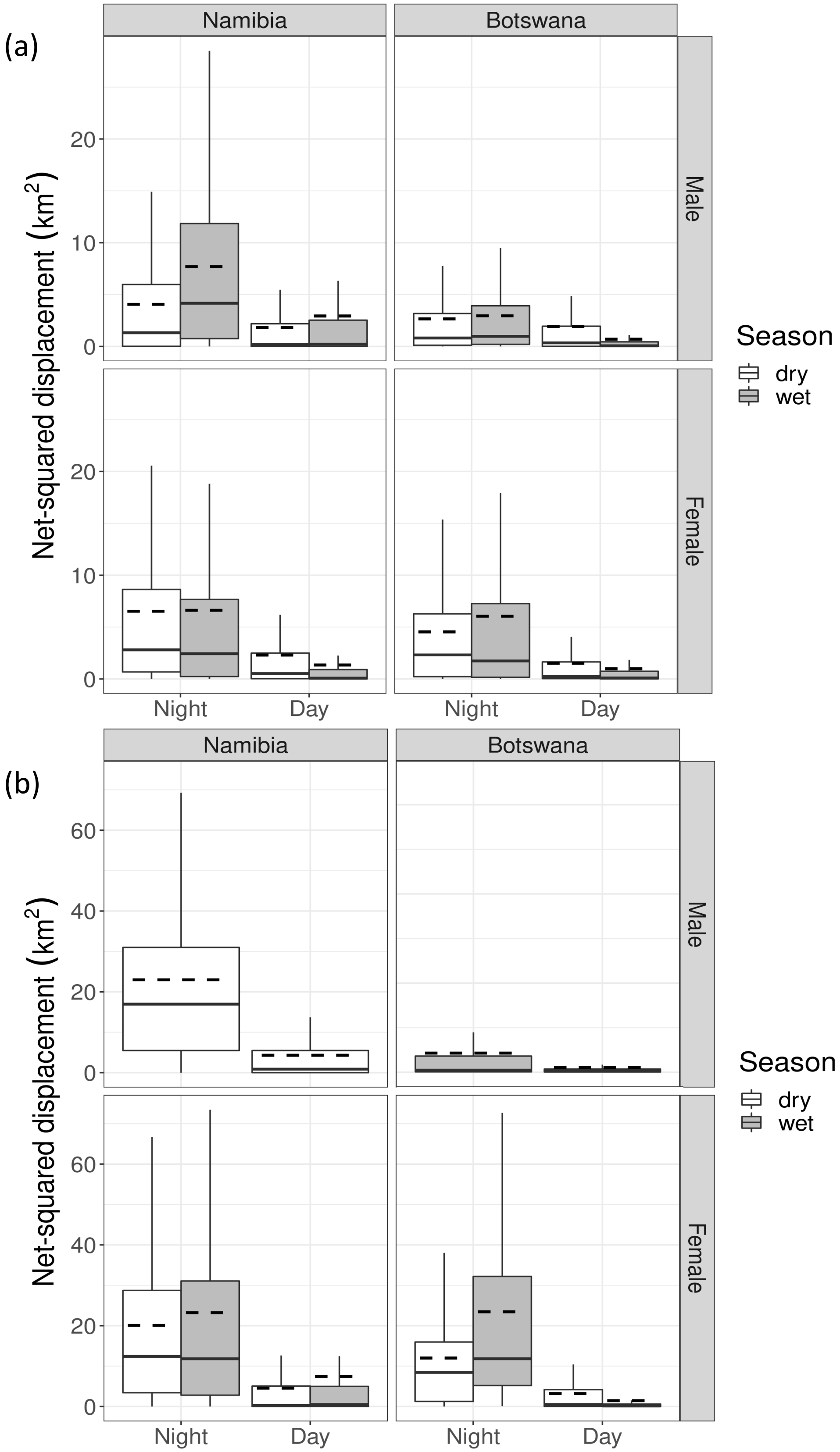

S10 Fig. Mean net-squared displacement (km^2) of (a) lions and (b) spotted hyenas between nocturnal (sunset – sunrise) and diurnal (sunrise – sunset) periods from the Etosha National Park, Namibia (left panels) and the Chobe National Park, Linyanti Conservancy, and Okavango Delta†, Botswana (right panels). Both figures, males top panels and females bottom panels. Boxplots show medians, 25% and 75% quartiles. Dashed lines indicate means. Whiskers indicate the IQR range.

†*No spotted hyenas were collared from the Okavango Delta, Botswana.*
