# Supplementary material for: Coursing hyenas and stalking lions: The potential for inter- and intraspecific interactions
Source: PLoS One. 2023 Feb 3;18(2):e0265054. doi: 10.1371/journal.pone.0265054 (PMC9897591; doi:10.1371/journal.pone.0265054)
Supplement: S11 Fig — Frequency density of seasonal turning angles for (a) lions and (b) spotted hyenas from the Etosha National Park, Namibia (left panels) and the Chobe National Park, Linyanti Conservancy, and Okavango Delta†, Botswana (right panels) during nocturnal (sunset – sunrise) and diurnal (sunrise – sunset) cycles. Both figures, males in upper panels and females in lower panels. Dry season = red lines, wet season = blue lines. †No spotted hyenas were collared from the Okavango Delta, Botswana. (PDF) [file pone.0265054.s027.pdf]

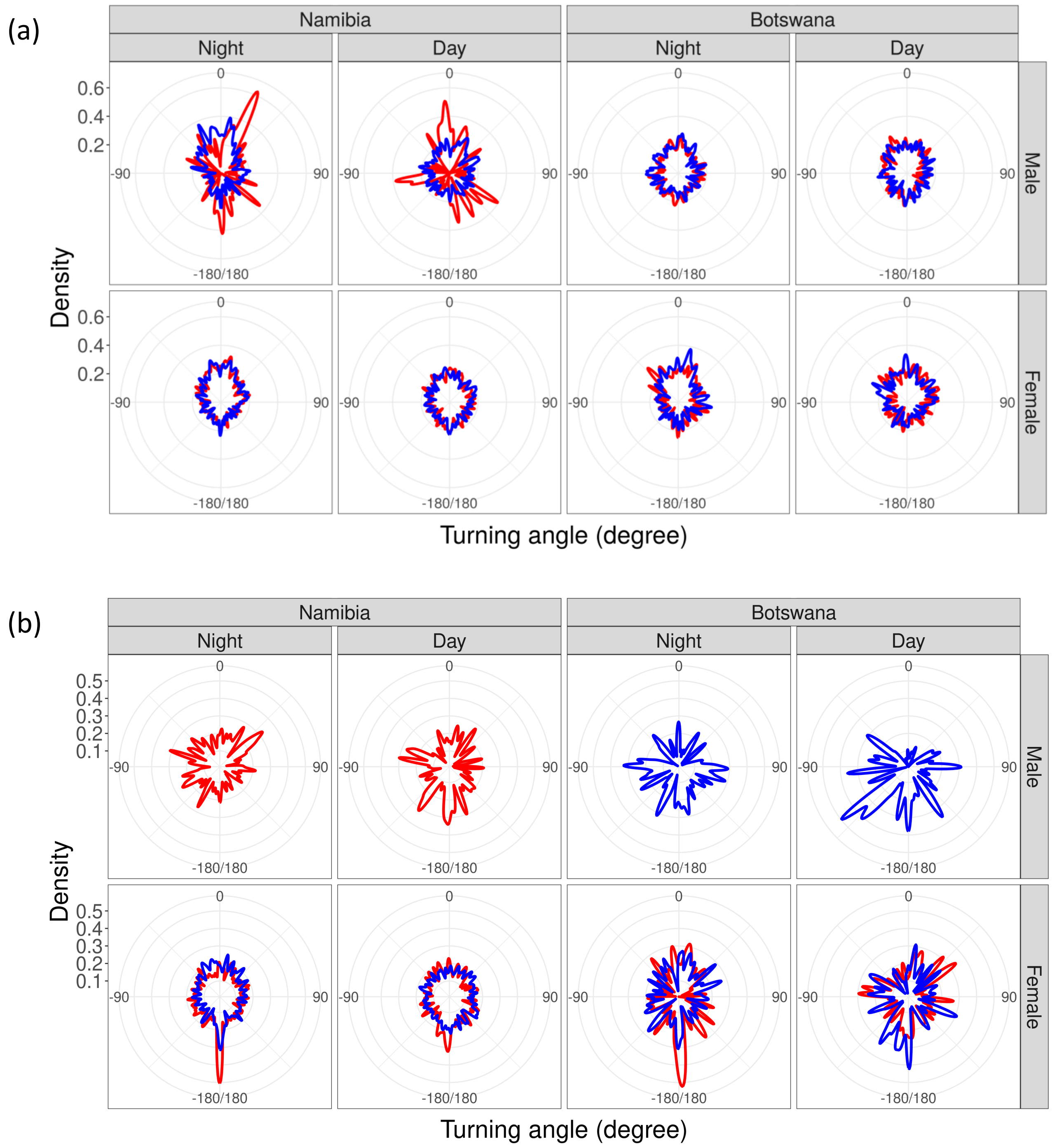

S11 Fig. Frequency density of seasonal turning angles for (a) lions and (b) spotted hyenas from the Etosha National Park, Namibia (left panels) and the Chobe National Park, Linyanti Conservancy, and Okavango Delta†, Botswana (right panels) during nocturnal (sunset – sunrise) and diurnal (sunrise – sunset) cycles. Both figures, males in upper panels and females in lower panels. Dry season = red lines, wet season = blue lines.

†*No spotted hyenas were collared from the Okavango Delta, Botswana.*
