# Supplementary material for: Coursing hyenas and stalking lions: The potential for inter- and intraspecific interactions
Source: PLoS One. 2023 Feb 3;18(2):e0265054. doi: 10.1371/journal.pone.0265054 (PMC9897591; doi:10.1371/journal.pone.0265054)
Supplement: S12 Fig — Frequency density of seasonal turning angles for (a) lions and (b) spotted hyenas during nocturnal (18h00-6h00 and 17h00-8h00) and dusk/dawn (19h00-21h00 and 4h00-6h00) periods. Etosha National Park, Namibia, left panels and Chobe National Park, Linyanti Conservancy, and Okavango Delta†, Botswana, right panels. Both figures, males upper panels and females lower panels. Dry season = red lines, wet season = blue lines. †No spotted hyenas were collared from the Okavango Delta, Botswana. (PDF) [file pone.0265054.s028.pdf]

(a)

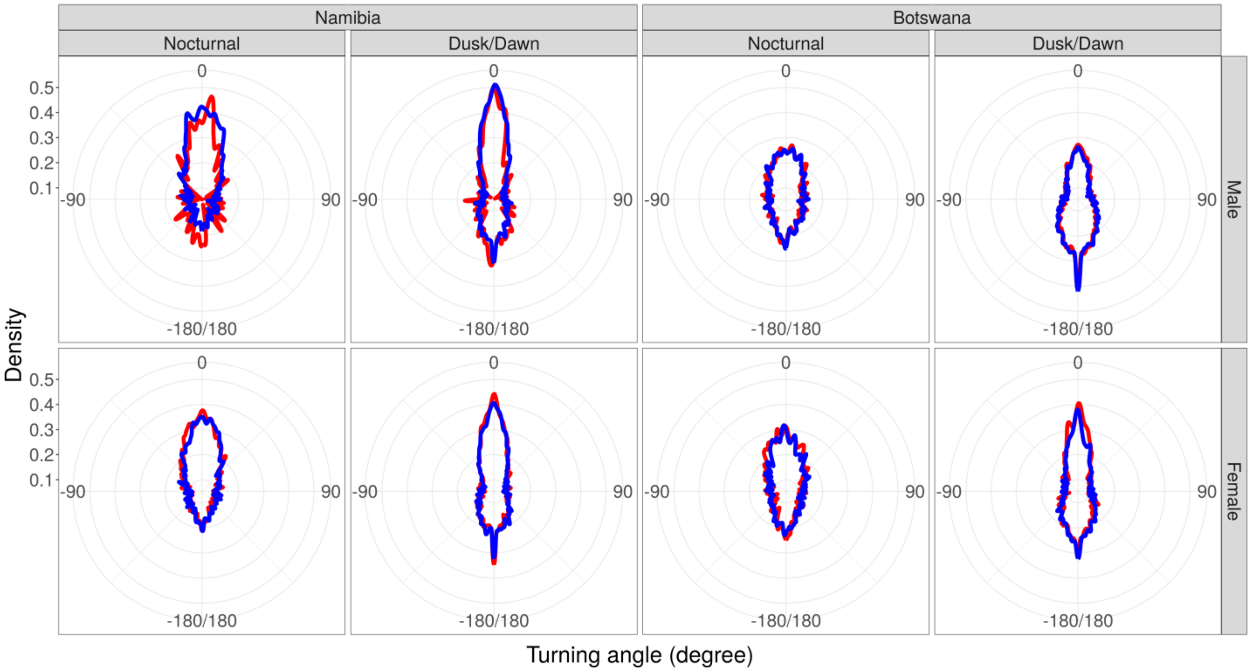

(b)

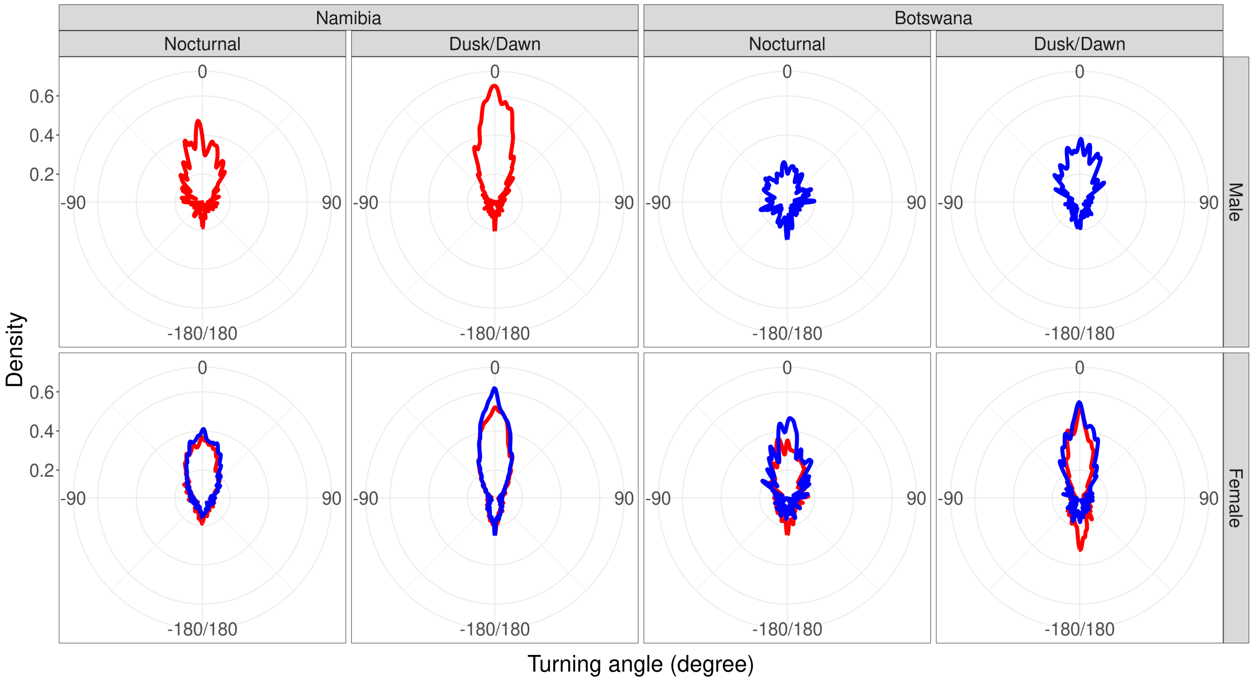

S12 Fig. Frequency density of seasonal turning angles for (a) lions and (b) spotted hyenas during nocturnal (18h00-6h00 and 17h00-8h00) and dusk/dawn (19h00-21h00 and 4h00-6h00) periods. Etosha National Park, Namibia, left panels and Chobe National Park, Linyanti Conservancy, and Okavango Delta†, Botswana, right panels. Both figures, males upper panels and females lower panels. Dry season = red lines, wet season = blue lines.

†*No spotted hyenas were collared from the Okavango Delta, Botswana.*
