# Supplementary material for: Coursing hyenas and stalking lions: The potential for inter- and intraspecific interactions
Source: PLoS One. 2023 Feb 3;18(2):e0265054. doi: 10.1371/journal.pone.0265054 (PMC9897591; doi:10.1371/journal.pone.0265054)
Supplement: S13 Fig — Frequency density of seasonal turning angles for (a) lions and (b) spotted hyenas during each hour of the nocturnal period (18h00-6h00) and during 4 hour blocks of the diurnal period (6h00-18h00). Species are from the Etosha National Park, Namibia (dry season = magenta lines; wet season = cyan lines), the Chobe National Park and Linyanti Conservancy, Botswana, with lions from the Okavango Delta, Botswana (dry season = red lines; wet season = blue lines). A double asterisk indicates a significant difference in the Watson’s Two-Sample Test of Homogeneity at p < 0.05, and a single asterisk approaches significance at 0.5 < p < 0.10. Placement of the asterisk(s) at the panel’s time label denotes the relatively more tortuous species for that time interval, with grey asterisk(s) for Namibia animals and black asterisk(s) for Botswana animals. (PDF) [file pone.0265054.s029.pdf]

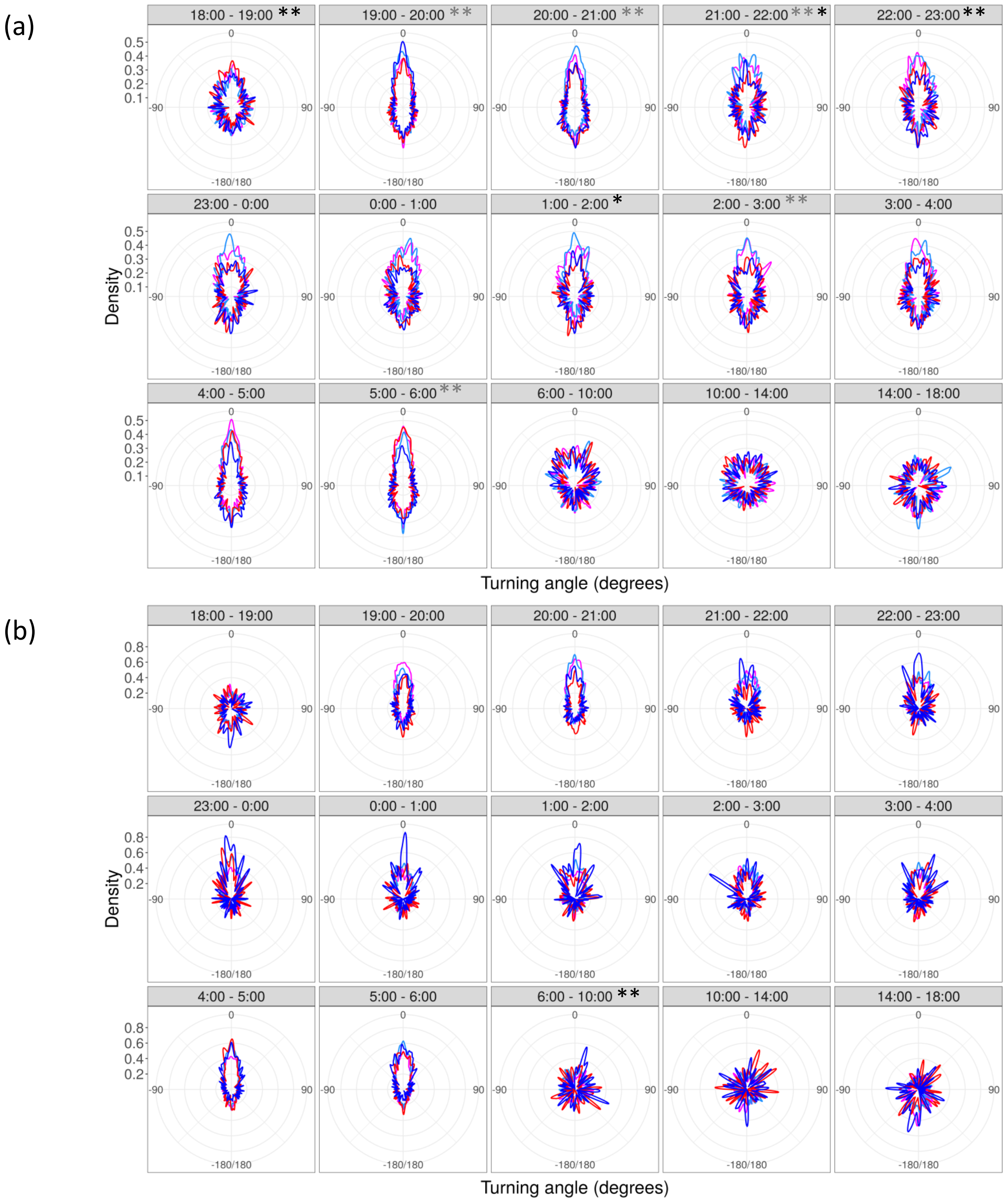

S13 Fig. Frequency density of seasonal turning angles for (a) lions and (b) spotted hyenas during each hour of the nocturnal period (18h00-6h00) and during 4-hour blocks of the diurnal period (6h00-18h00). Species are from the Etosha National Park, Namibia (dry season = magenta lines; wet season = cyan lines), the Chobe National Park and Linyanti Conservancy, Botswana, with lions from the Okavango Delta, Botswana (dry season = red lines; wet season = blue lines). A double asterisk indicates a significant difference in the Watson's Two-Sample Test of Homogeneity at $p < 0.05$, and a single asterisk approaches significance at $0.5 < p < 0.10$. Placement of the asterisk(s) at the panel's time label denotes the relatively more tortuous species for that time interval, with grey asterisk(s) for Namibia animals and black asterisk(s) for Botswana animals.
