# Supplementary material for: Coursing hyenas and stalking lions: The potential for inter- and intraspecific interactions
Source: PLoS One. 2023 Feb 3;18(2):e0265054. doi: 10.1371/journal.pone.0265054 (PMC9897591; doi:10.1371/journal.pone.0265054)
Supplement: S14 Fig — Frequency density of seasonal turning angles for (a) Etosha lions and (b) Chobe/ Linyanti spotted hyenas during the nocturnal period according to various moon phases. Darkest lunar phase (far left panel) increasing to brightest lunar phase (far right panel). (PDF) [file pone.0265054.s030.pdf]

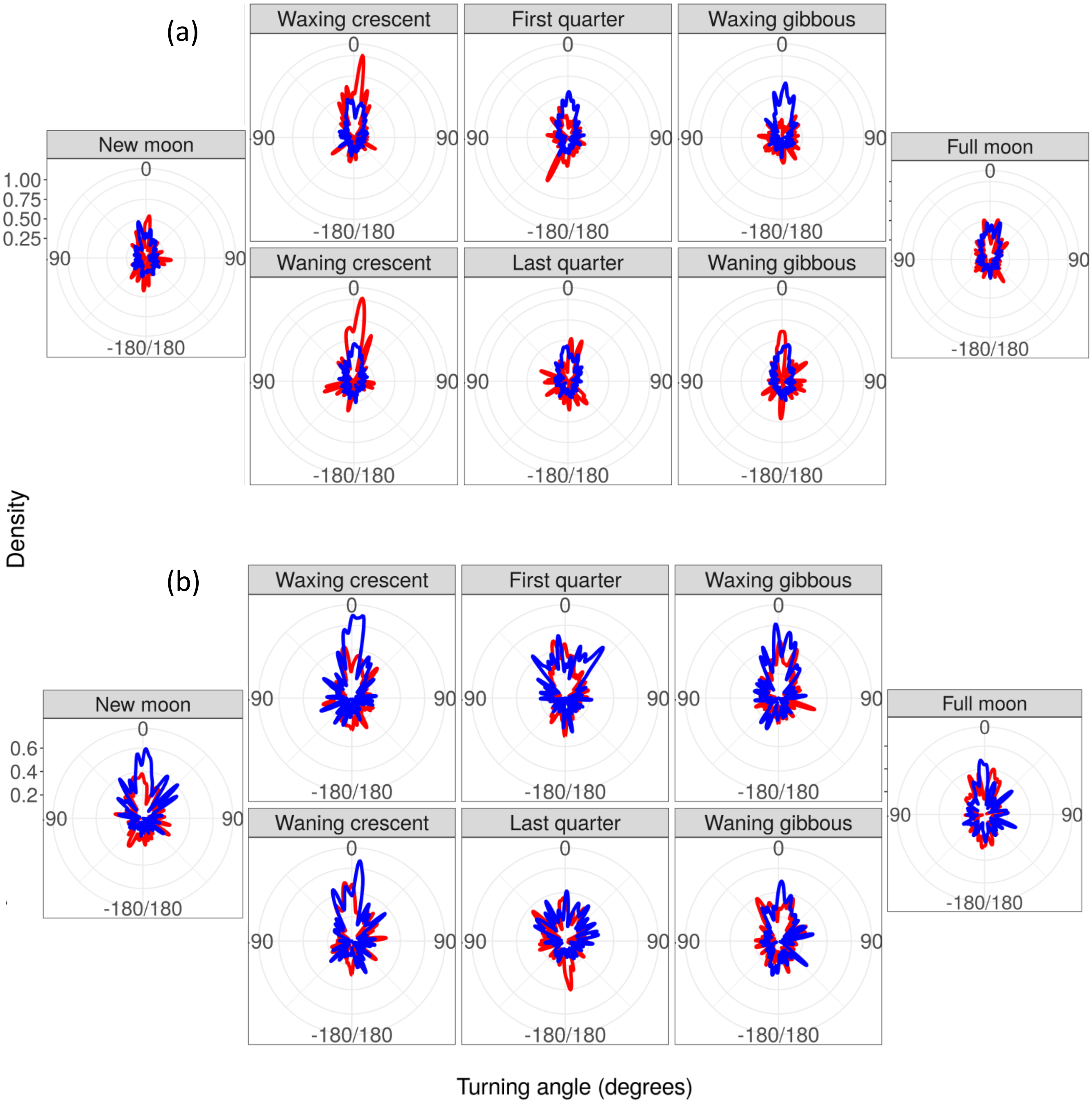

S14 Fig. Frequency density of seasonal turning angles for (a) Etosha lions and (b) Chobe/ Linyanti spotted hyenas during the nocturnal period according to various moon phases. Darkest lunar phase (far left panel) increasing to brightest lunar phase (far right panel).
