# Supplementary material for: Coursing hyenas and stalking lions: The potential for inter- and intraspecific interactions
Source: PLoS One. 2023 Feb 3;18(2):e0265054. doi: 10.1371/journal.pone.0265054 (PMC9897591; doi:10.1371/journal.pone.0265054)
Supplement: S15 Fig — Tortuosity of (a) lions and (b) spotted hyenas from the Etosha National Park, Namibia (i & ii) and the Chobe National Park and Linyanti Conservancy, Botswana (iii & iv). Path tortuosity is depicted at different distance intervals from competitors (i & iii), and conspecifics (ii & iv). (PDF) [file pone.0265054.s031.pdf]

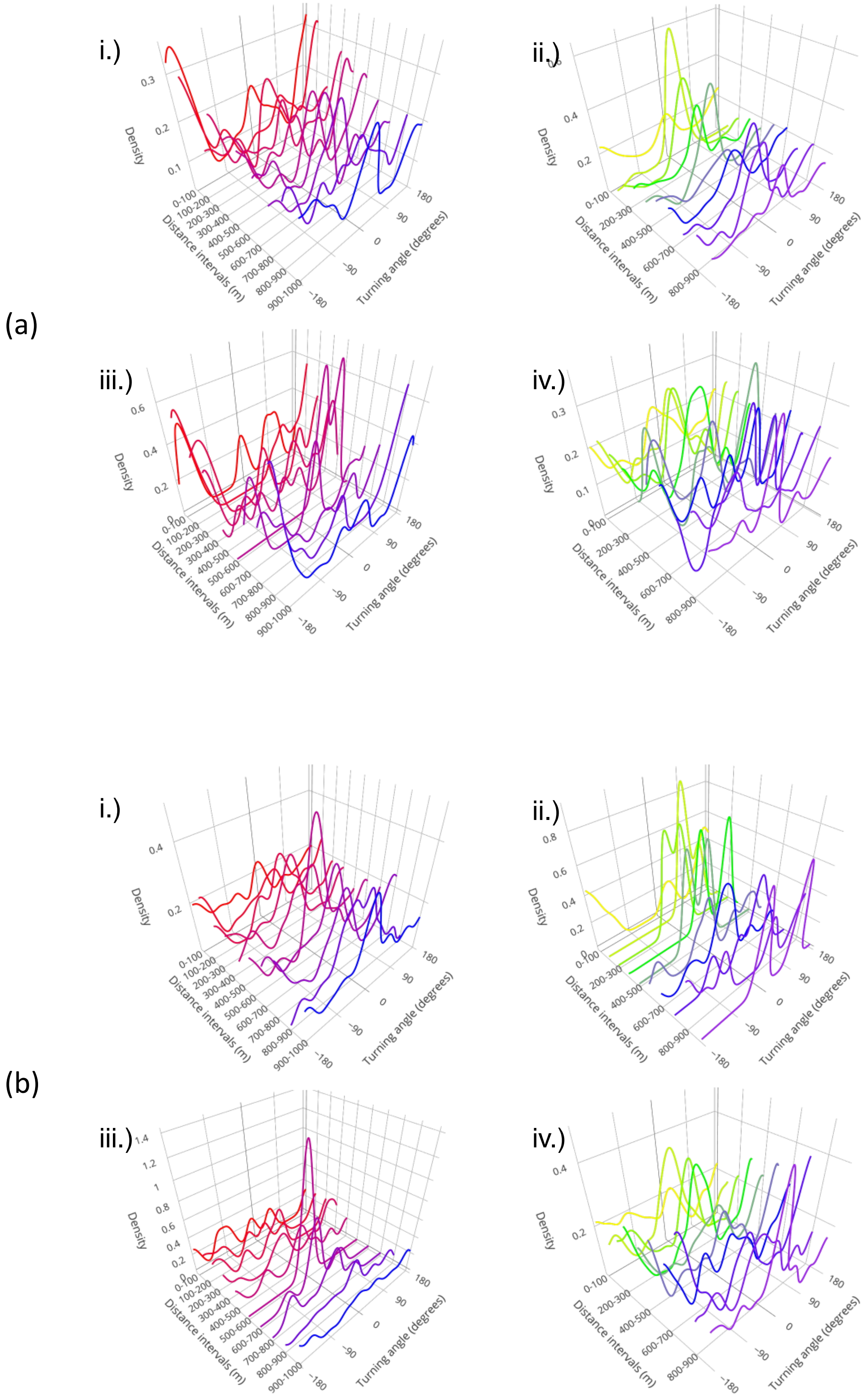

S15 Fig. Tortuosity of (a) lions and (b) spotted hyenas from the Etosha National Park, Namibia (i & ii) and the Chobe National Park and Linyanti Conservancy, Botswana (iii & iv). Path tortuosity is depicted at different distance intervals from competitors (i & iii), and conspecifics (ii & iv).
