# Supplementary material for: Coursing hyenas and stalking lions: The potential for inter- and intraspecific interactions
Source: PLoS One. 2023 Feb 3;18(2):e0265054. doi: 10.1371/journal.pone.0265054 (PMC9897591; doi:10.1371/journal.pone.0265054)
Supplement: S16 Fig — Time-use constructs of lion individuals from the (a) Etosha National Park, Namibia; (b) Chobe National Park; (c) Linyanti Conservancy; and (d) Okavango Delta, Botswana. Unique identifiers are depicted vertically on the left of each row of figures. α-LoCoH hulls of individual’s utilization distributions (far left). Hull parent points colored by visitation rate (nsv, number of separate visits; second from left), and duration of visit (mnlv, mean number of locations in the hull per visit; third from left). RD space scatterplots (second from right) with X-axis = visitation rate (nsv), and Y-axis = duration of visit (mnlv), provide a legend for revisitation/duration (RD) values for the map (far right). Points in the RD space have been jiggled to better see point density, and each point represents a hull. Points on the maps are colored by their location in the RD space. Separate visits are defined by an inter-visit gap period ≥ 12 hours. Hulls were created using the adaptive method. Duplicate points are offset by 1 map unit. (PDF) [file pone.0265054.s032.pdf]

(a)

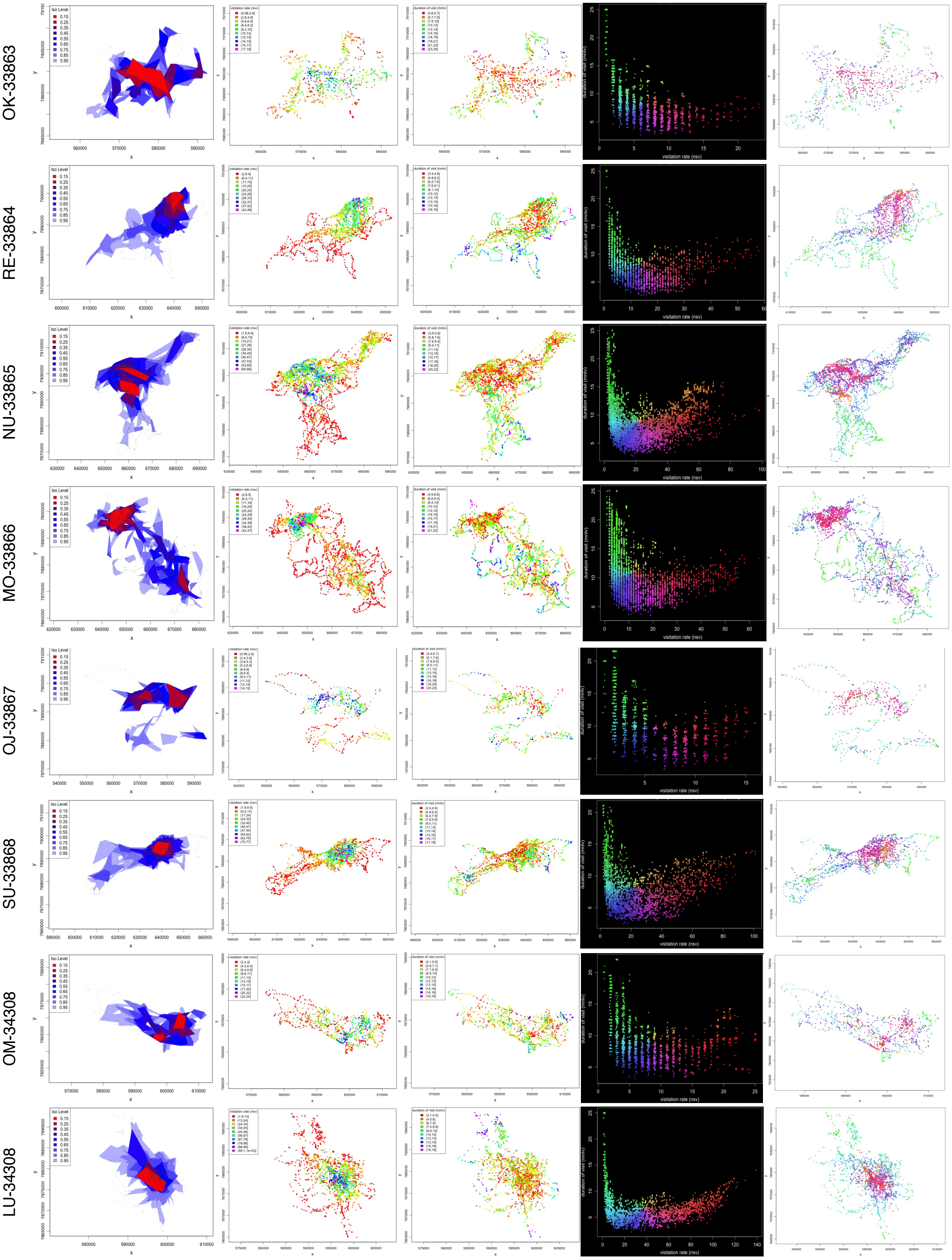
OK-33863
RE-33864
NU-33865
MO-33866
OJ-33867
SU-33868
OM-34308
LU-34308
Iso Level
visitation rate (nsv)
duration of visit (mnlv)
x
y

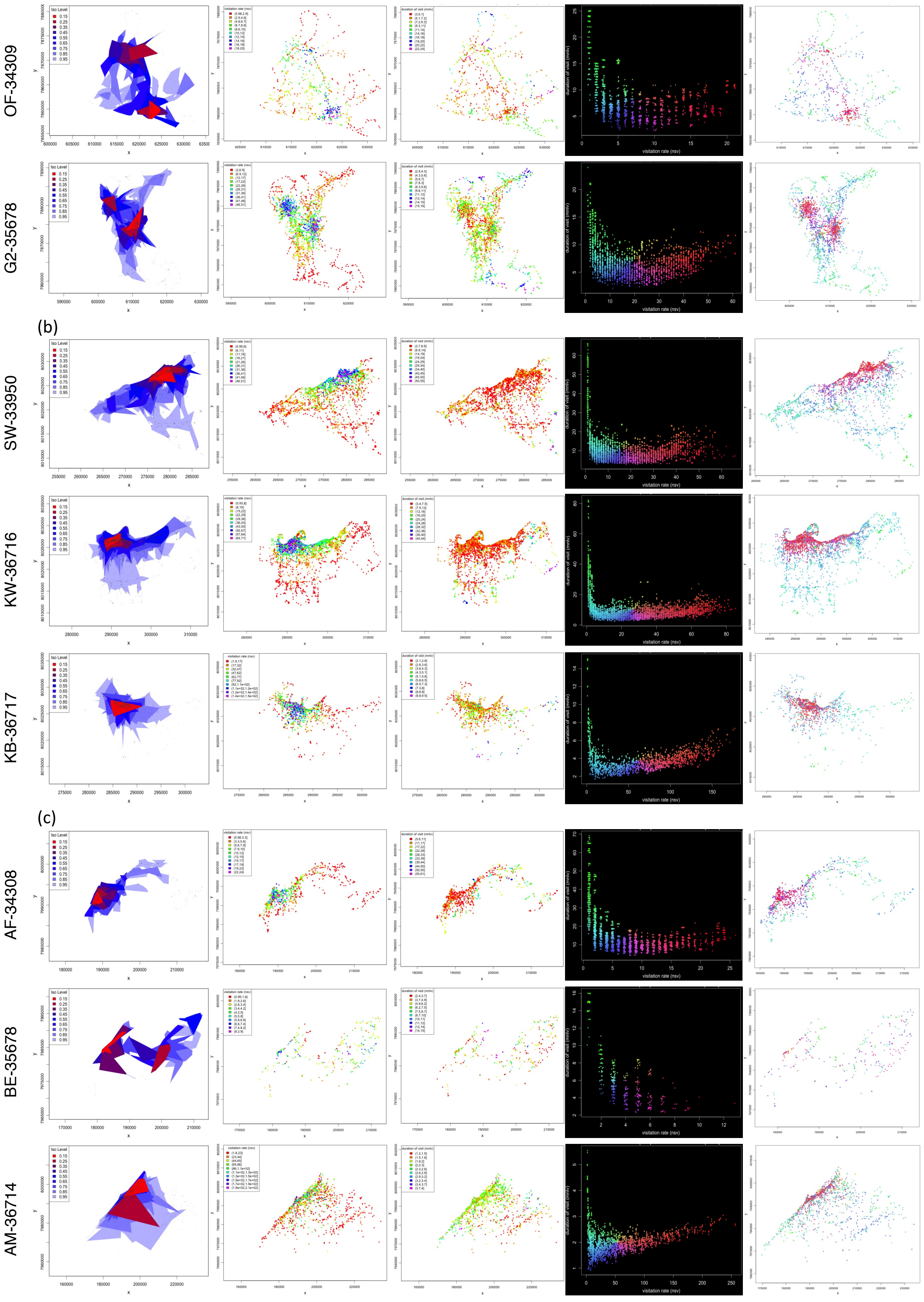
OF-34309
G2-35678
(b)
SW-33950
KW-36716
KB-36717
(c)
AF-34308
BE-35678
AM-36714
Iso Level
visitation rate (nsv)
duration of visit (mnlv)

(d)

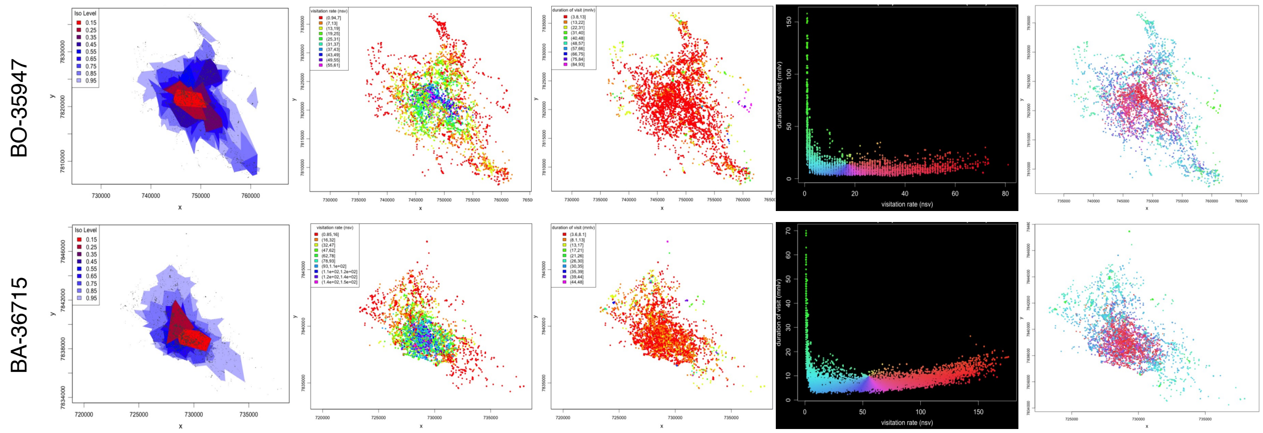

S16 Fig. Lion revisitation and duration (RD) space plots. Time-use constructs of lion individuals from the (a) Etosha National Park, Namibia; (b) Chobe National Park; (c) Linyanti Conservancy; and (d) Okavango Delta, Botswana. Unique identifiers are depicted vertically on the left of each row of figures. α-LoCoH hulls of individual's utilization distributions (far left). Hull parent points coloured by visitation rate (nsv, number of separate visits; second from left), and duration of visit (mnlv, mean number of locations in the hull per visit; third from left). RD space scatterplots (second from right) with X-axis = visitation rate (nsv), and Y-axis = duration of visit (mnlv), provide a legend for revisitation/duration (RD) values for the map (far right). Points in the RD space have been jiggled to better see point density, and each point represents a hull. Points on the maps are coloured by their location in the RD space. Separate visits are defined by an inter-visit gap period ≥ 12hours. Hulls were created using the adaptive method. Duplicate points are offset by 1 map unit.
