# Supplementary material for: Coursing hyenas and stalking lions: The potential for inter- and intraspecific interactions
Source: PLoS One. 2023 Feb 3;18(2):e0265054. doi: 10.1371/journal.pone.0265054 (PMC9897591; doi:10.1371/journal.pone.0265054)
Supplement: S18 Fig — Maps (left panels) depict the individual lion’s relocations as four (top row), six (middle row), and eight (bottom row) clusters in the (a) Etosha National Park, Namibia; (b) Chobe National Park; (c) Linyanti Conservancy; and (d) Okavango Delta, Botswana. Unique identifiers are depicted on top corner of each page. Relocations are color-coded according to the clusters indicated by the range of revisitation (number of separate visits) and duration (mean number of locations per visit) values in RD space plots (shown in central panels). Clusters in the RD space were determined with the k-prototype algorithm and are based on ecogeographical variables attached to each relocation. The smaller plots (right panels) present the distribution and percent category of clusters for each of the ecogeographical variables selected from the factor analysis of mixed data (FAMD). (PDF) [file pone.0265054.s034.pdf]

(a.1) OK-33863

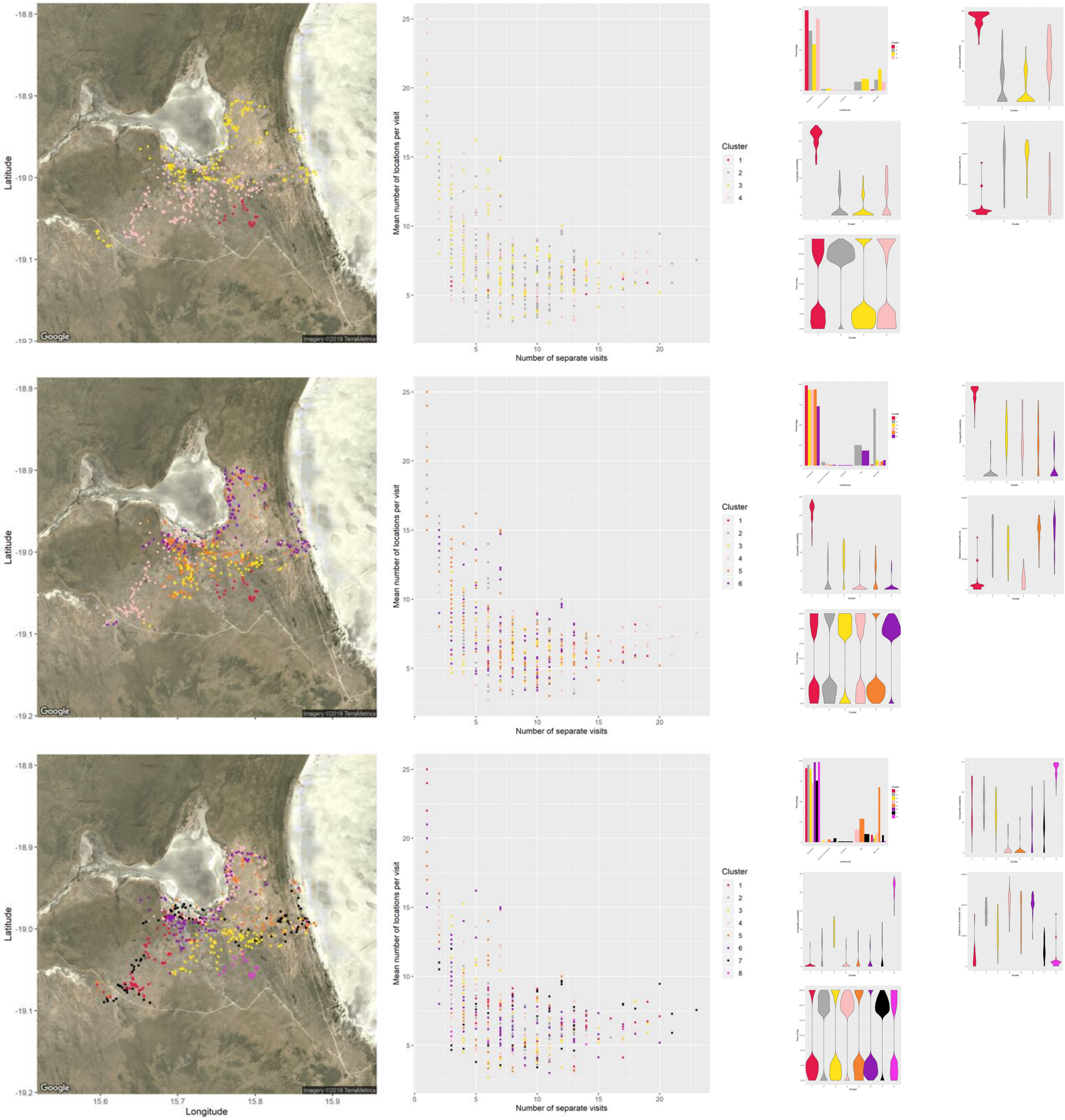

Latitude
Longitude
-18.8
-18.9
-19.0
-19.1
-19.2
15.6
15.7
15.8
15.9
Google
Imagery ©2019 TerraMetrics
Mean number of locations per visit
Number of separate visits
0
5
10
15
20
25
Cluster
1
2
3
4
5
6
7
8

(a.2) RE-33864

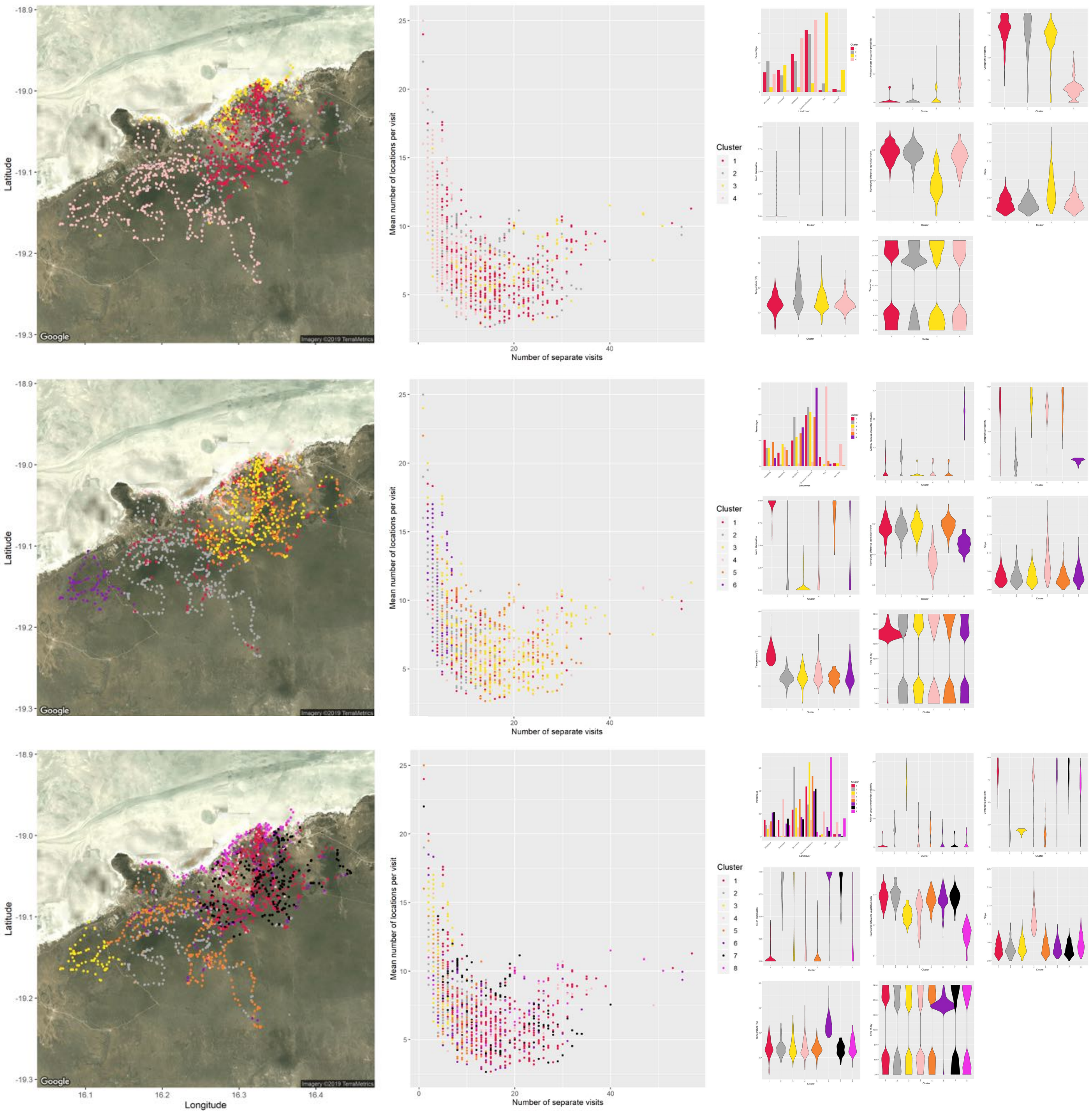

Latitude
Longitude
-18.9
-19.0
-19.1
-19.2
-19.3
16.1
16.2
16.3
16.4
Google
Imagery ©2019 TerraMetrics
Mean number of locations per visit
Number of separate visits
0
20
40
5
10
15
20
25
Cluster
1
2
3
4
5
6
7
8

(a.3) NU-33865

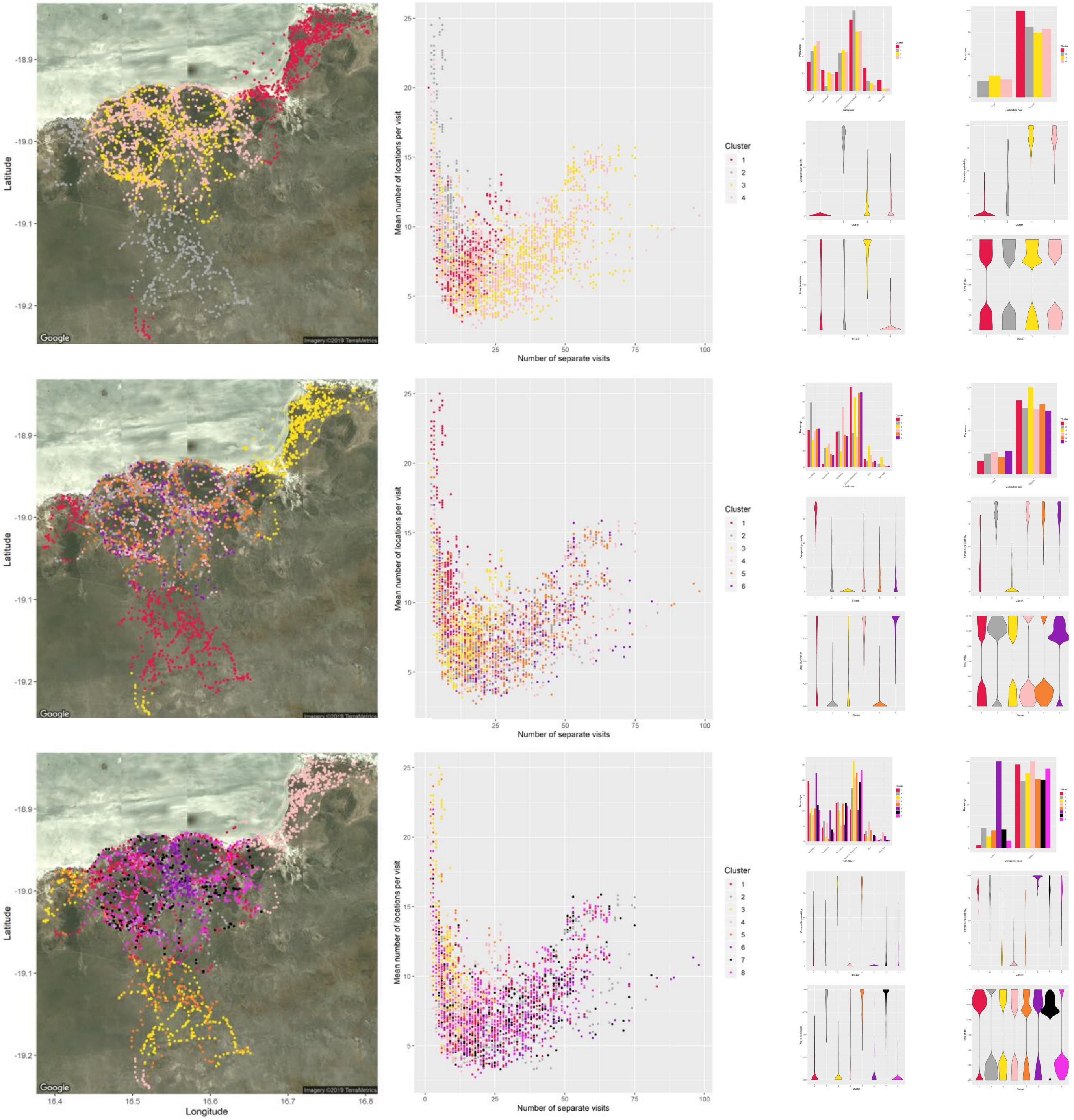

Latitude
Longitude
-18.9
-19.0
-19.1
-19.2
16.4
16.5
16.6
16.7
16.8
Google
Imagery ©2019 TerraMetrics
Mean number of locations per visit
Number of separate visits
0
5
10
15
20
25
50
75
100
Cluster
1
2
3
4
5
6
7
8

(a.4) MO-33866

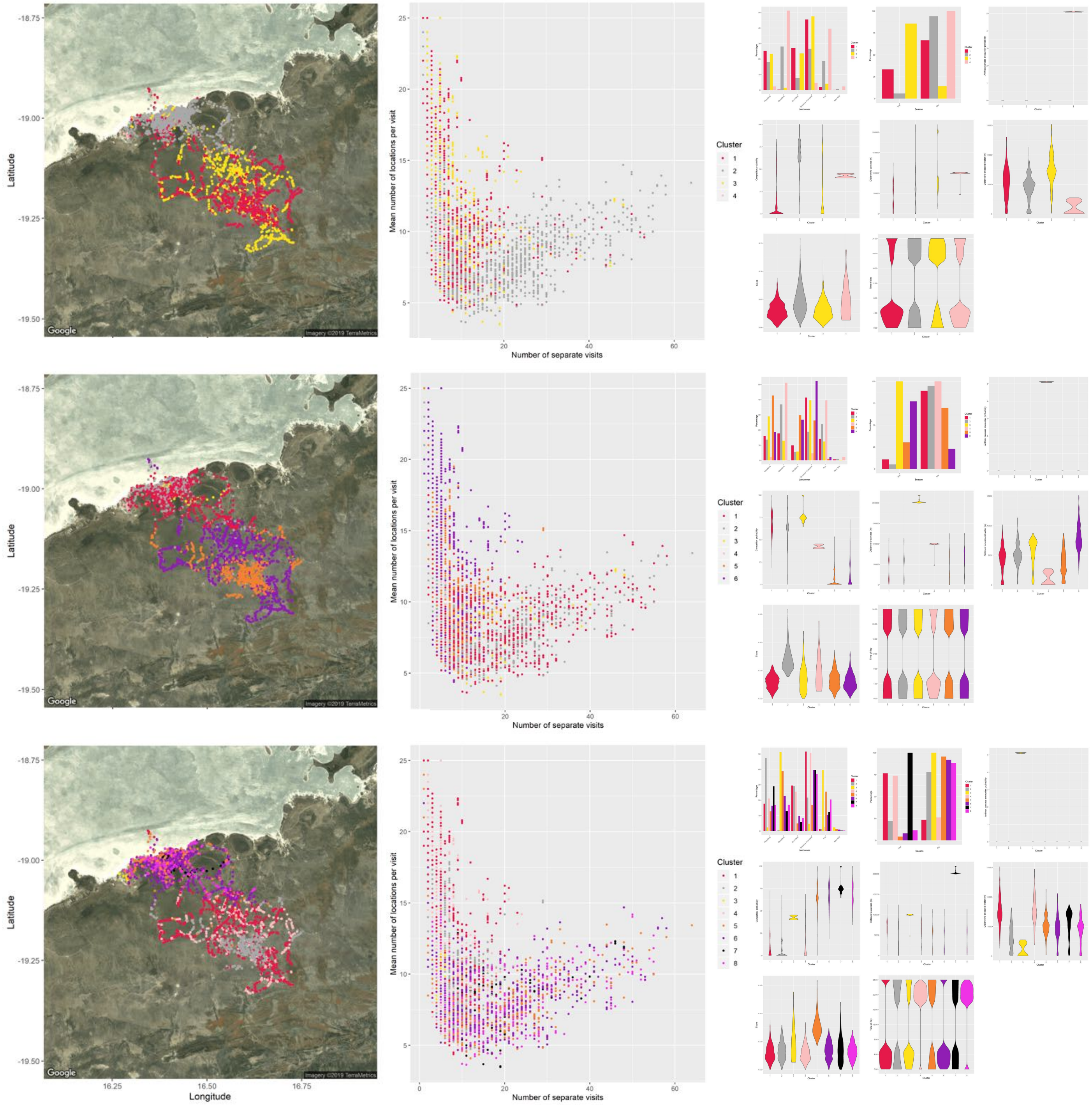

-18.75
-19.00
-19.25
-19.50
Latitude
Google
Imagery ©2019 TerraMetrics
25
20
15
10
5
Mean number of locations per visit
20
40
60
Number of separate visits
Cluster
1
2
3
4
5
6
7
8
16.25
16.50
16.75
Longitude

(a.5) OJ-33867

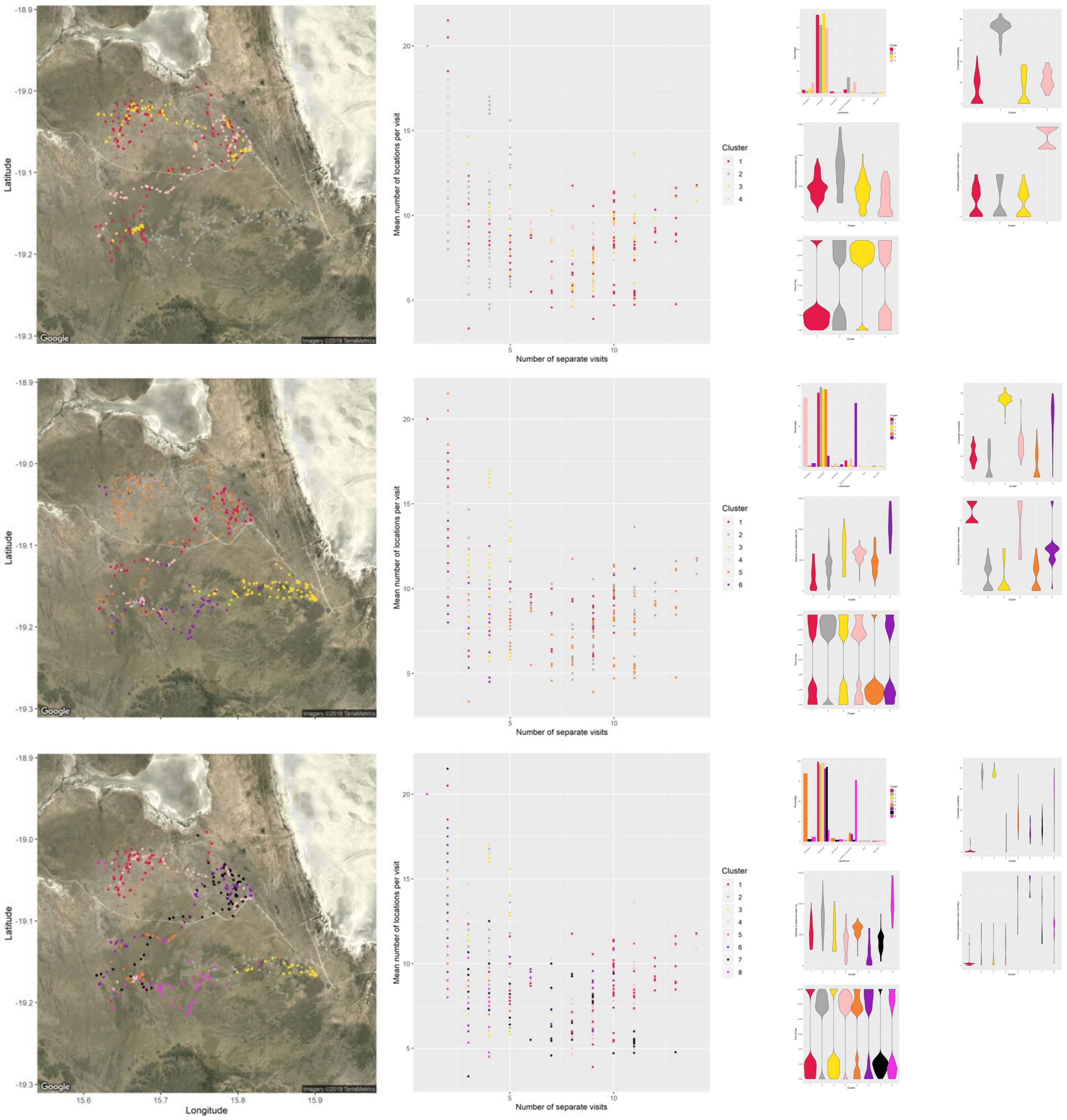

Latitude
Longitude
15.6
15.7
15.8
15.9
-18.9
-19.0
-19.1
-19.2
-19.3
Google
Imagery ©2019 TerraMetrics
Mean number of locations per visit
Number of separate visits
Cluster
1
2
3
4
5
6
7
8

(a.6) SU-33868

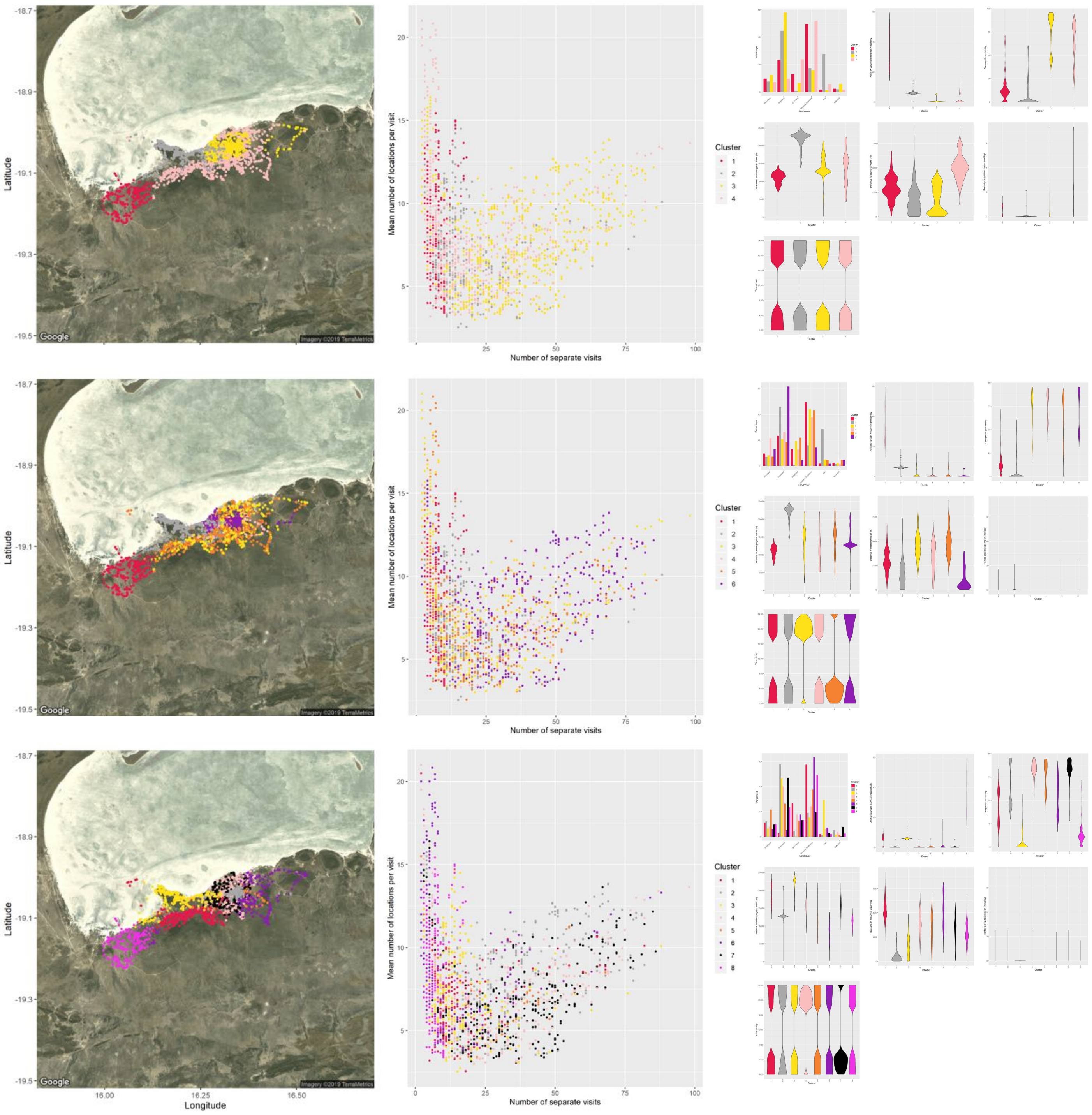
Latitude
Longitude
-18.7
-18.9
-19.1
-19.3
-19.5
16.00
16.25
16.50
Google
Imagery ©2019 TerraMetrics
Mean number of locations per visit
Number of separate visits
0
25
50
75
100
5
10
15
20
Cluster
1
2
3
4
5
6
7
8

(a.7) OM-34308

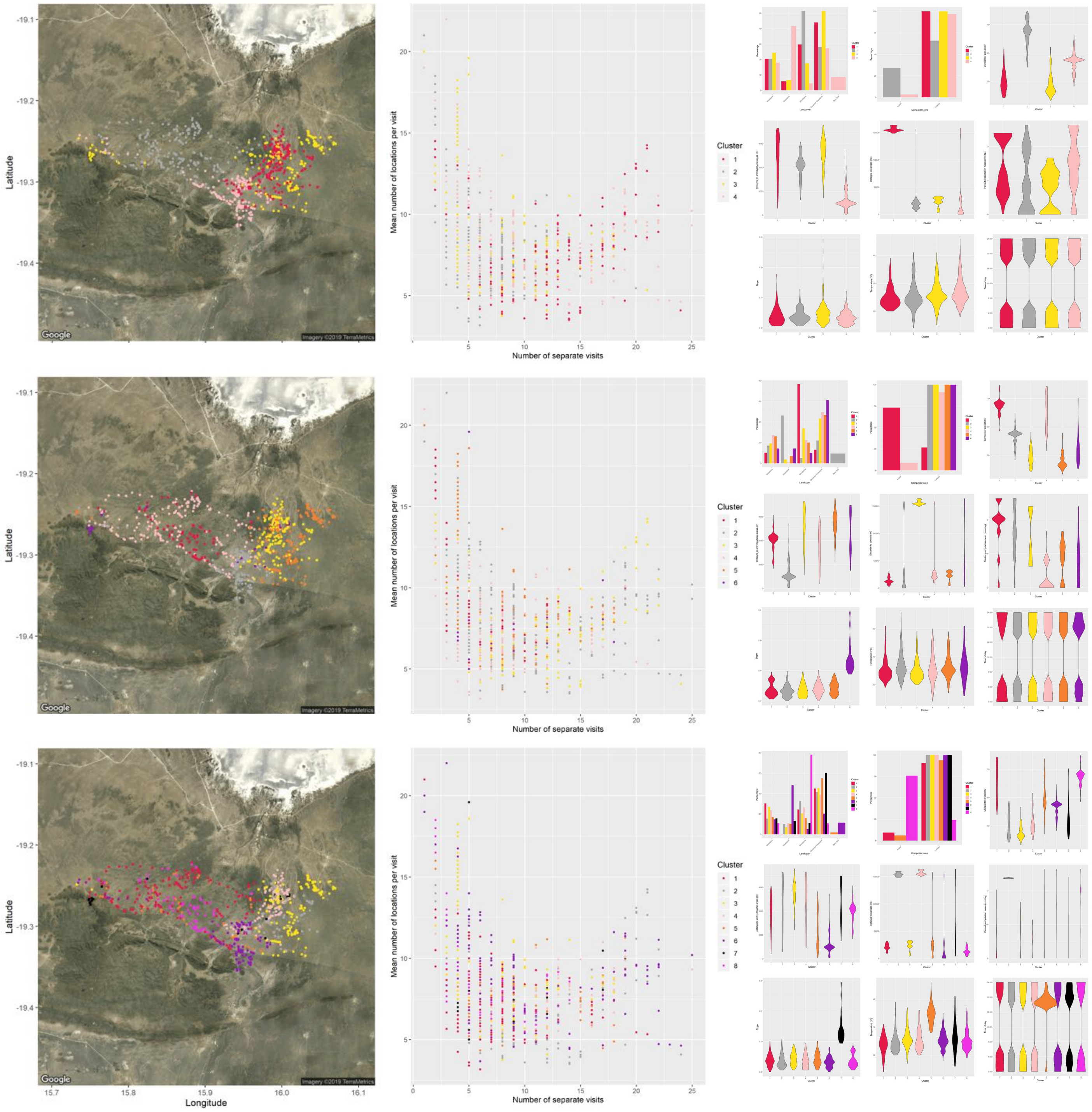

Latitude
-19.1
-19.2
-19.3
-19.4
Longitude
15.7
15.8
15.9
16.0
16.1
Google
Imagery ©2019 TerraMetrics
Mean number of locations per visit
Number of separate visits
0
5
10
15
20
25
Cluster
1
2
3
4
5
6
7
8

(a.8) LU-34308

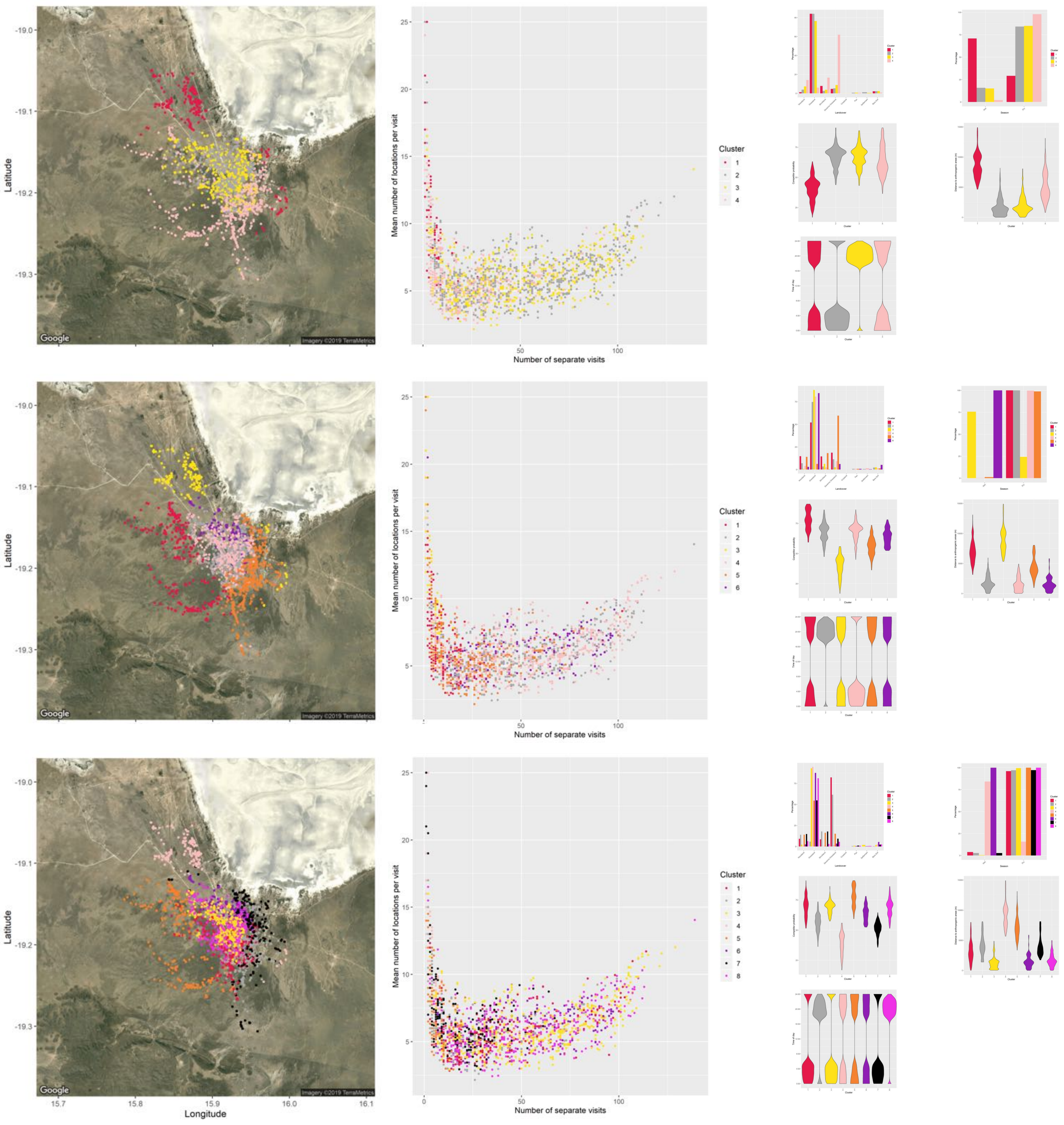

Latitude
-19.0
-19.1
-19.2
-19.3
Longitude
15.7
15.8
15.9
16.0
16.1
Google
Imagery ©2019 TerraMetrics
Mean number of locations per visit
25
20
15
10
5
Number of separate visits
0
50
100
Cluster
1
2
3
4
5
6
7
8

(a.9) OF-34309

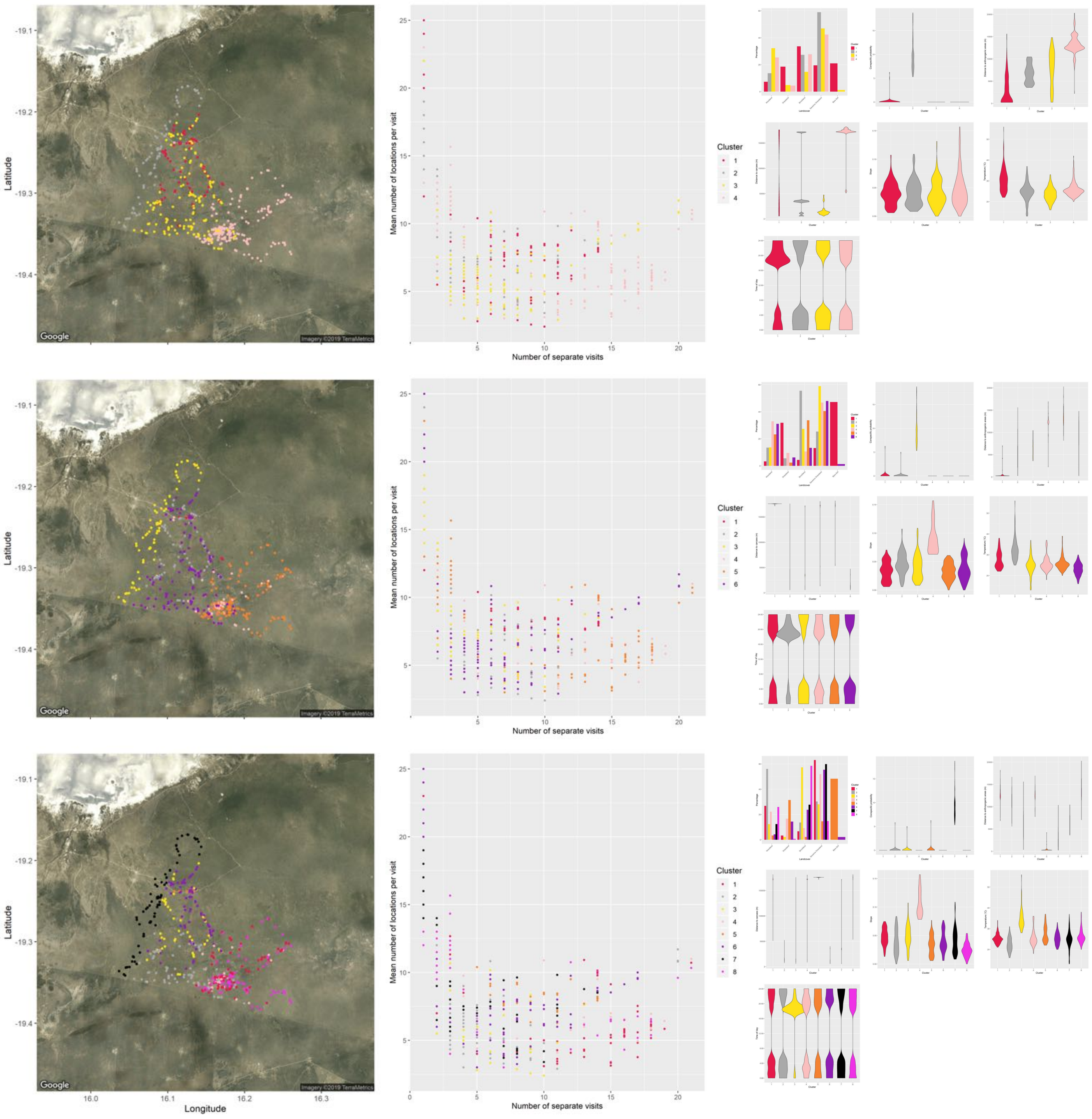

Latitude
-19.1
-19.2
-19.3
-19.4
Google
Imagery ©2019 TerraMetrics
Mean number of locations per visit
Number of separate visits
Cluster
1
2
3
4
5
6
7
8
Longitude
16.0
16.1
16.2
16.3

(a.10) G2-35678

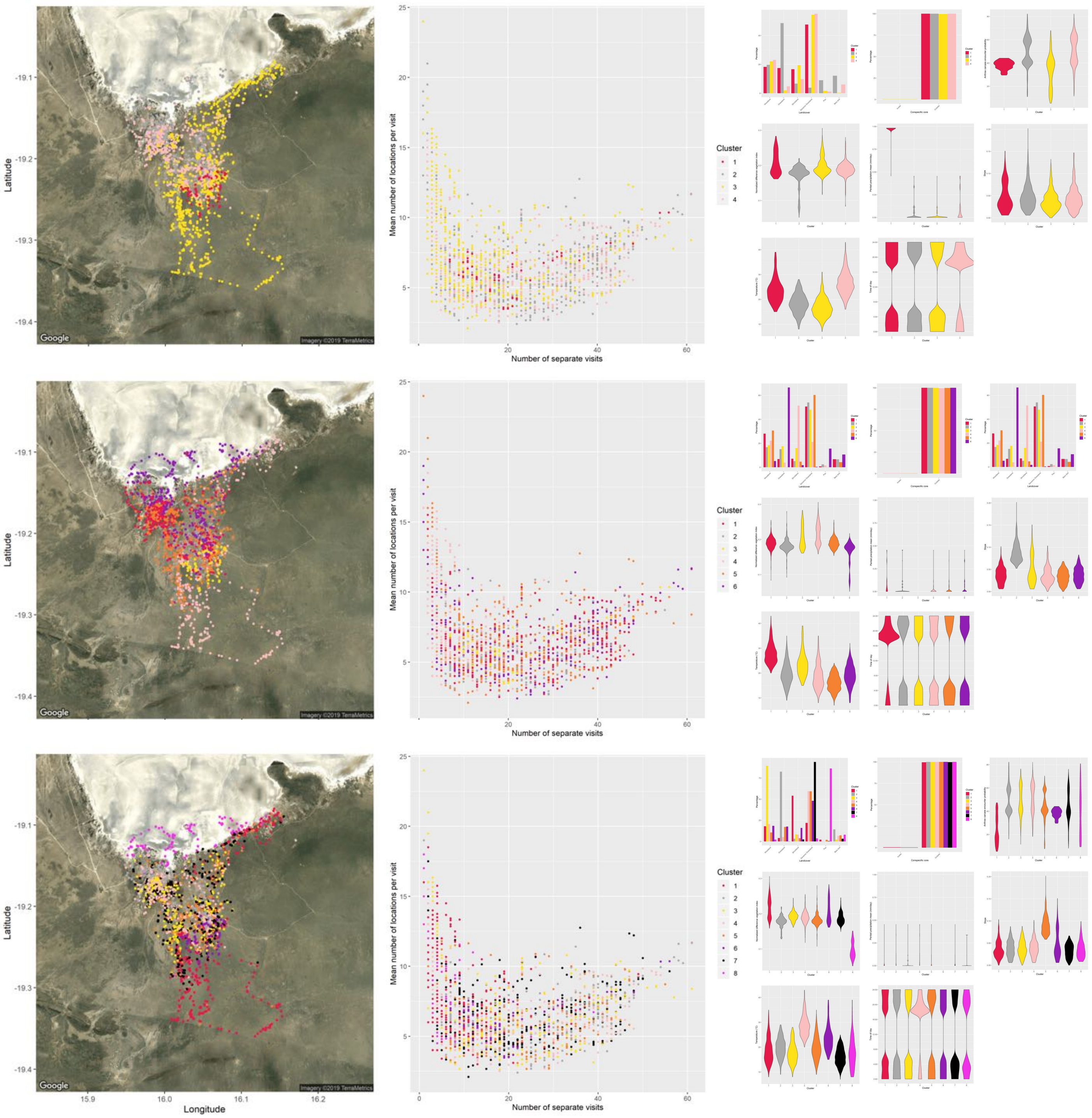
Latitude
Longitude
-19.1
-19.2
-19.3
-19.4
15.9
16.0
16.1
16.2
Google
Imagery ©2019 TerraMetrics
Mean number of locations per visit
Number of separate visits
0
20
40
60
5
10
15
20
25
Cluster
1
2
3
4
5
6
7
8

(b.1) SW-33950

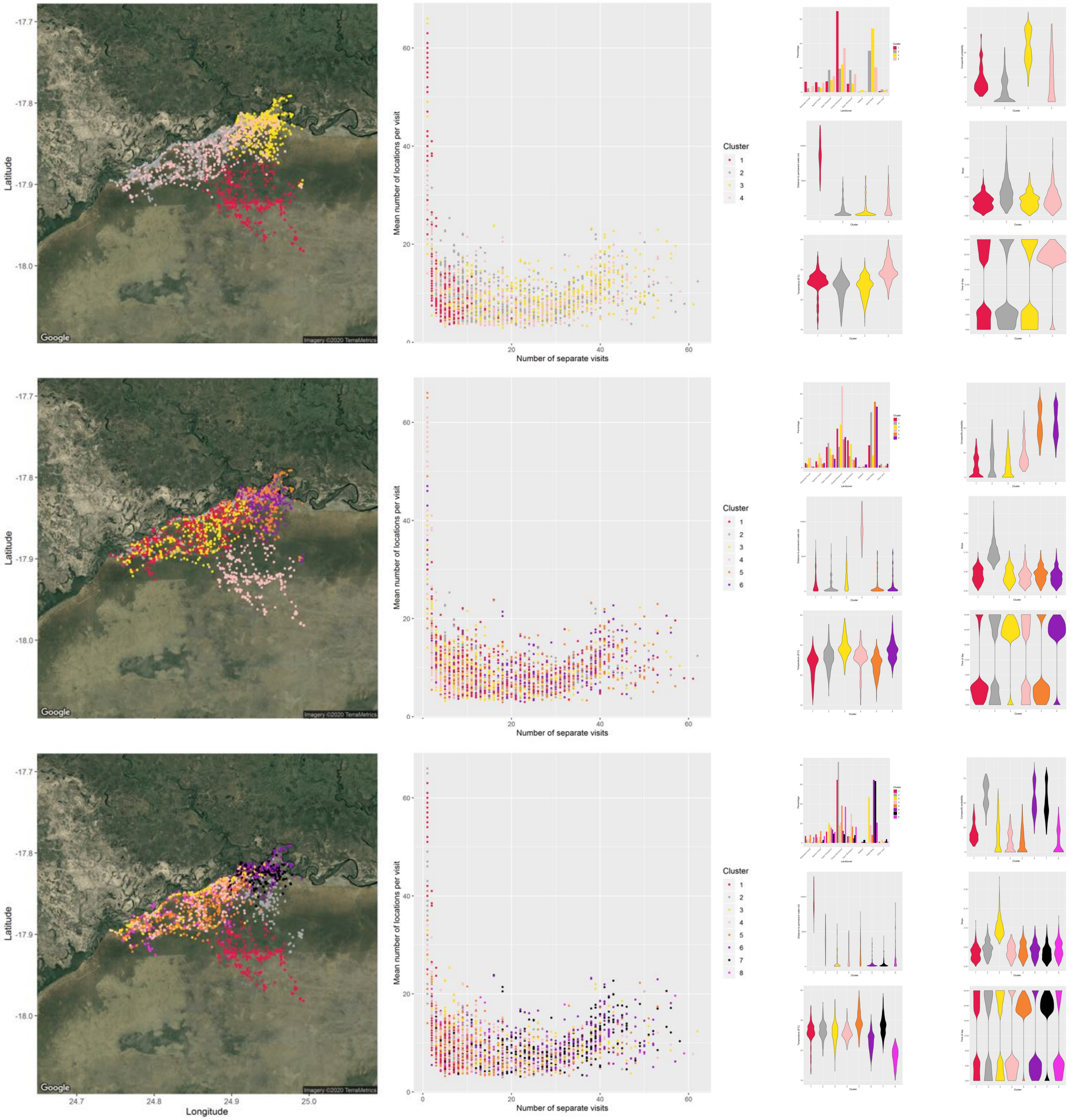

Latitude
Longitude
-17.7
-17.8
-17.9
-18.0
24.7
24.8
24.9
25.0
Google
Imagery ©2020 TerraMetrics
Mean number of locations per visit
Number of separate visits
0
20
40
60
Cluster
1
2
3
4
5
6
7
8

(b.2) KW-36716

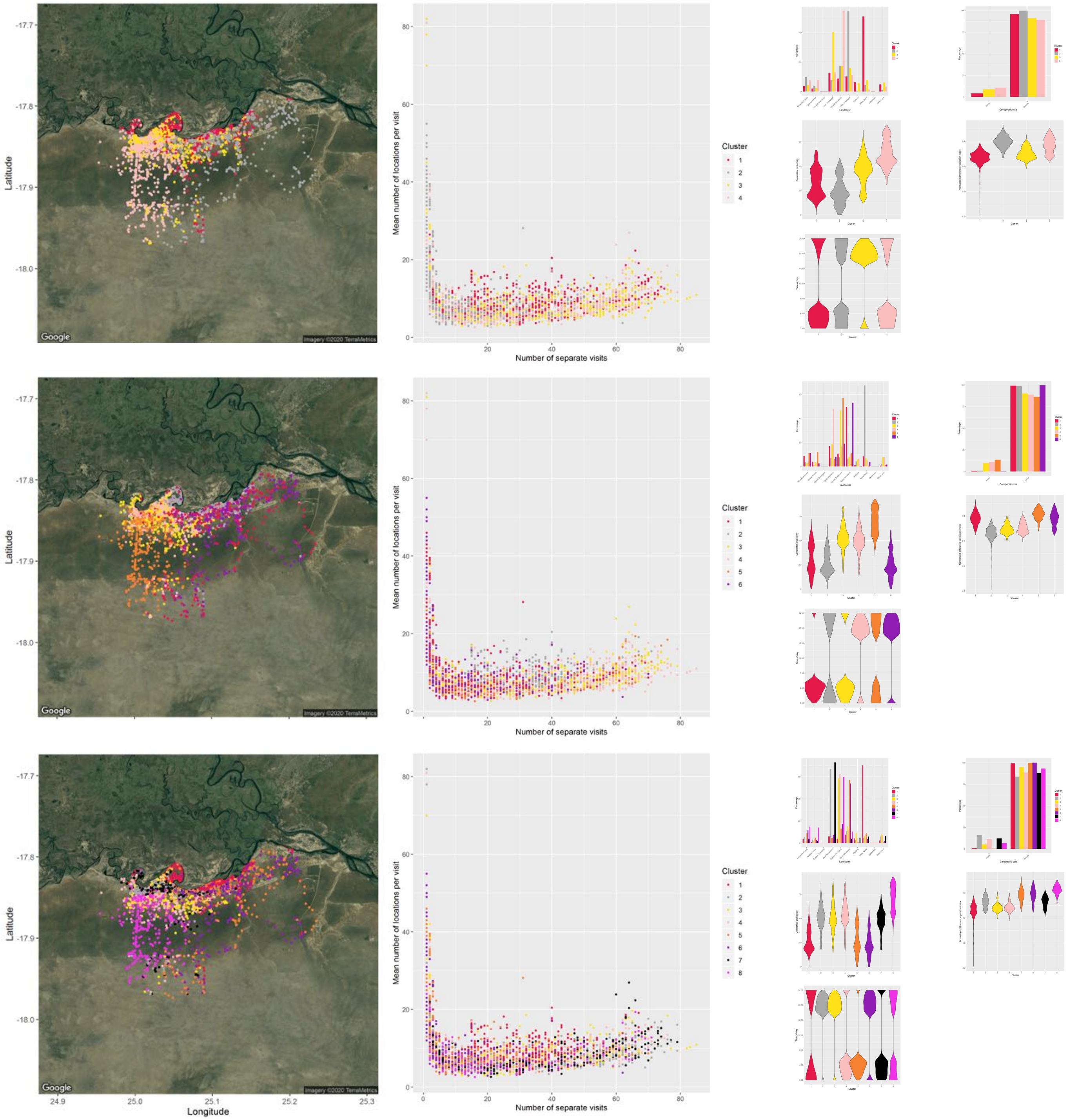
Latitude
Longitude
Mean number of locations per visit
Number of separate visits
Cluster
Google
Imagery ©2020 TerraMetrics

(b.3) KB-36717

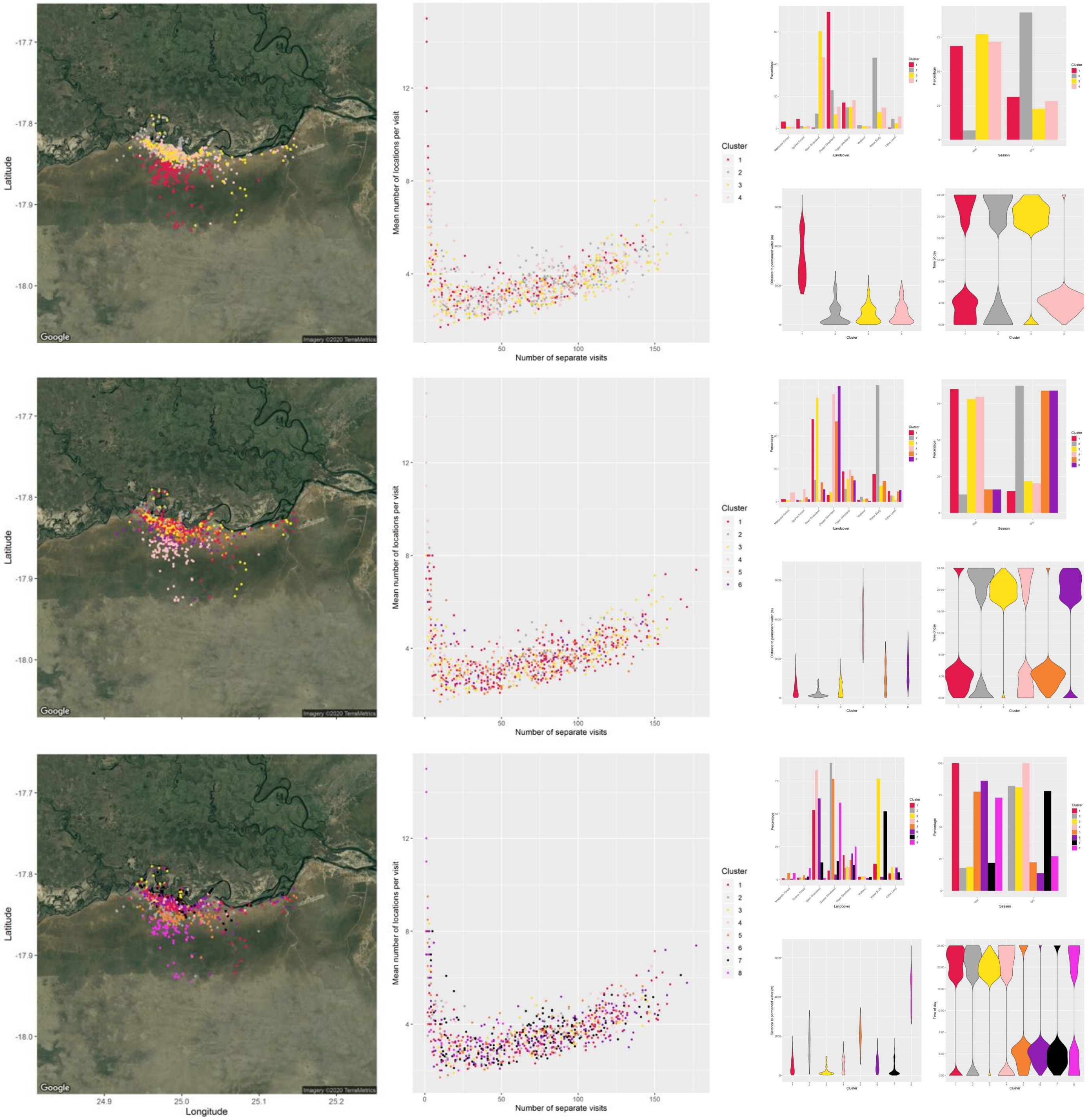

Latitude
Longitude
-17.7
-17.8
-17.9
-18.0
24.9
25.0
25.1
25.2
Google
Imagery ©2020 TerraMetrics
Mean number of locations per visit
Number of separate visits
0
50
100
150
4
8
12
Cluster
1
2
3
4
5
6
7
8
Percentage
Landcover
Season
Distance to permanent water (m)
Time of day

(c.1) AF-34308

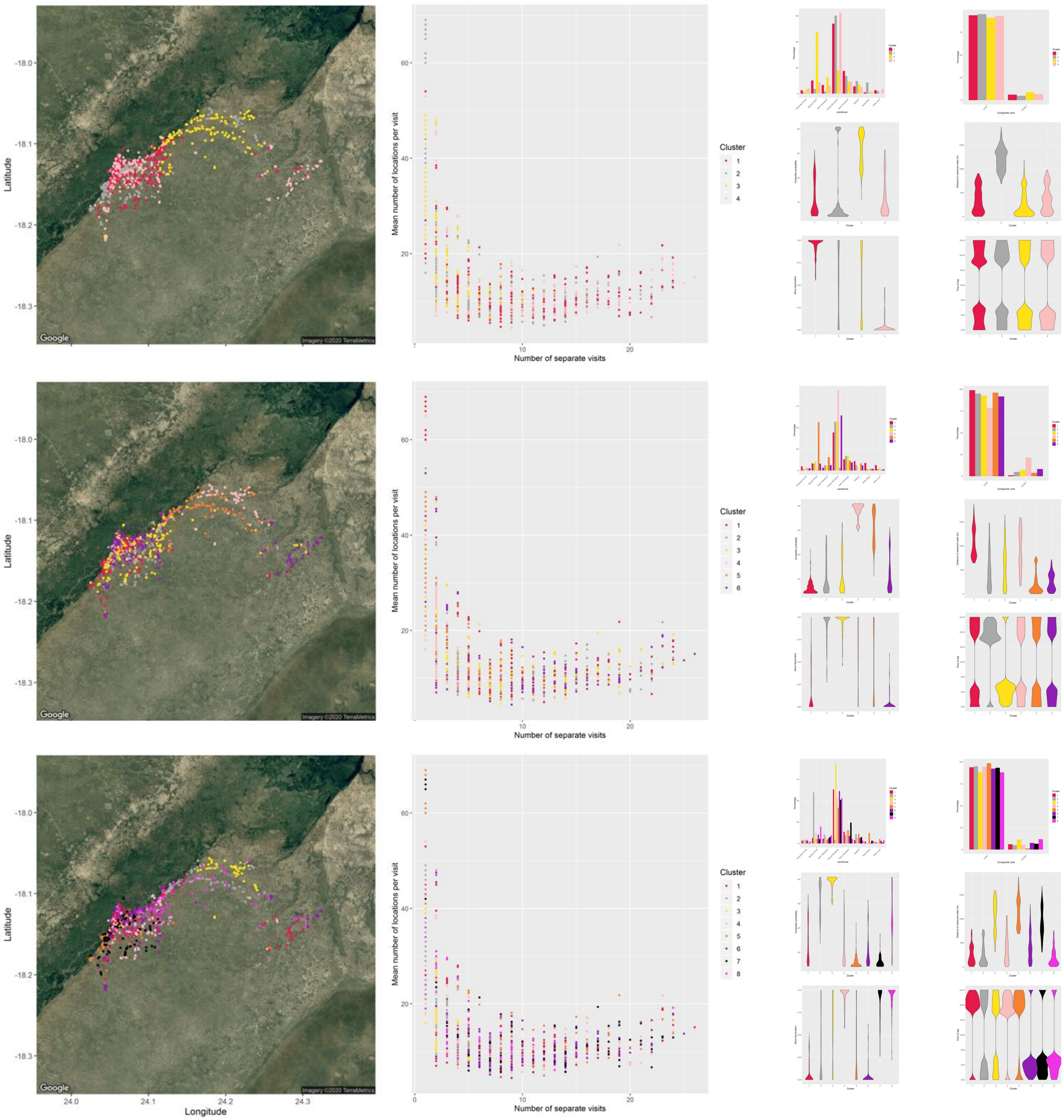

Latitude
-18.0
-18.1
-18.2
-18.3
Longitude
24.0
24.1
24.2
24.3
Google
Imagery ©2020 TerraMetrics
Mean number of locations per visit
60
40
20
Number of separate visits
10
20
Cluster
1
2
3
4
5
6
7
8

(c.2) BE-35678

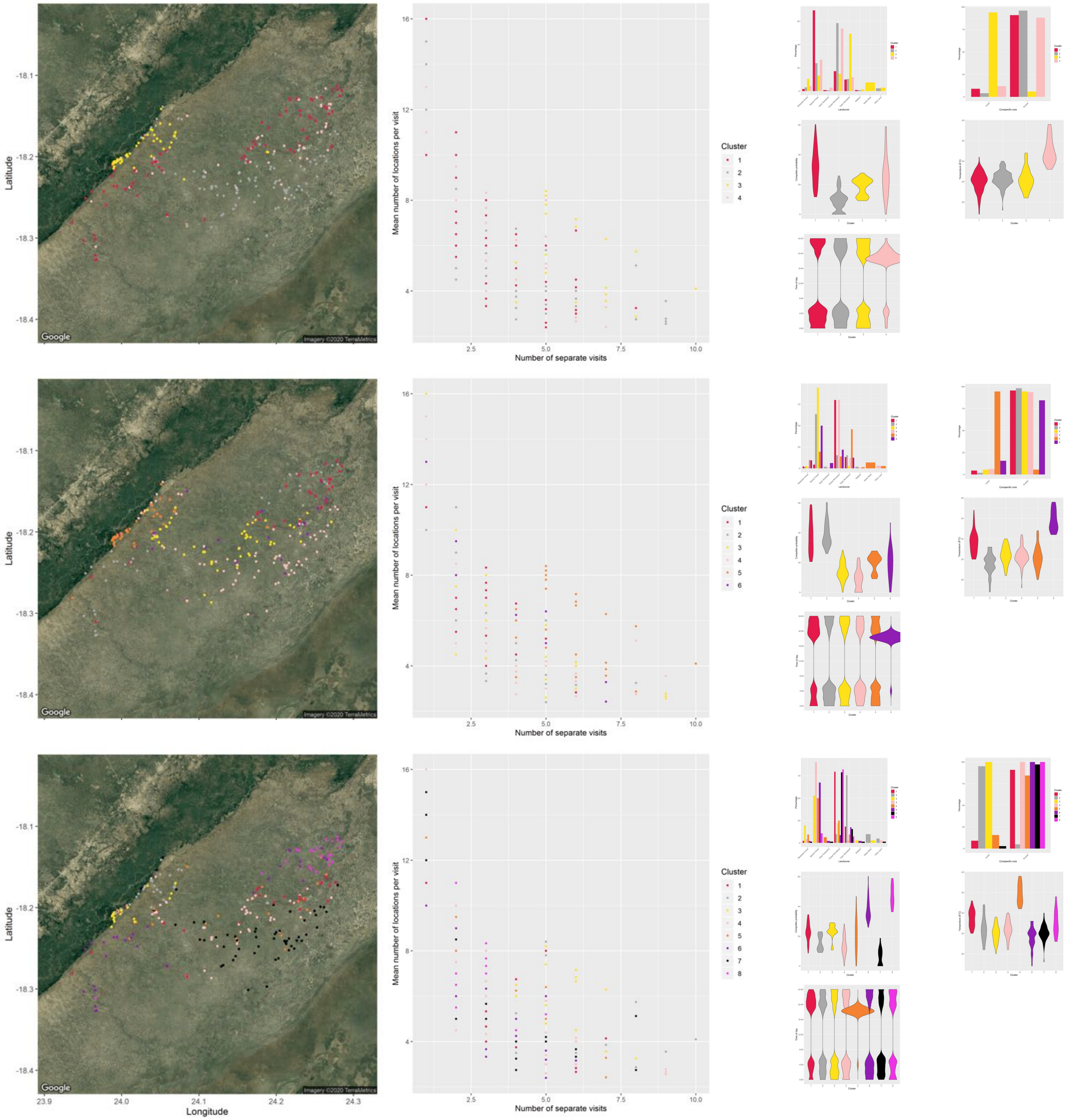

Latitude
Longitude
-18.1
-18.2
-18.3
-18.4
23.9
24.0
24.1
24.2
24.3
Google
Imagery ©2020 TerraMetrics
Mean number of locations per visit
Number of separate visits
4
8
12
16
2.5
5.0
7.5
10.0
Cluster
1
2
3
4
5
6
7
8

(c.3) AM-36714

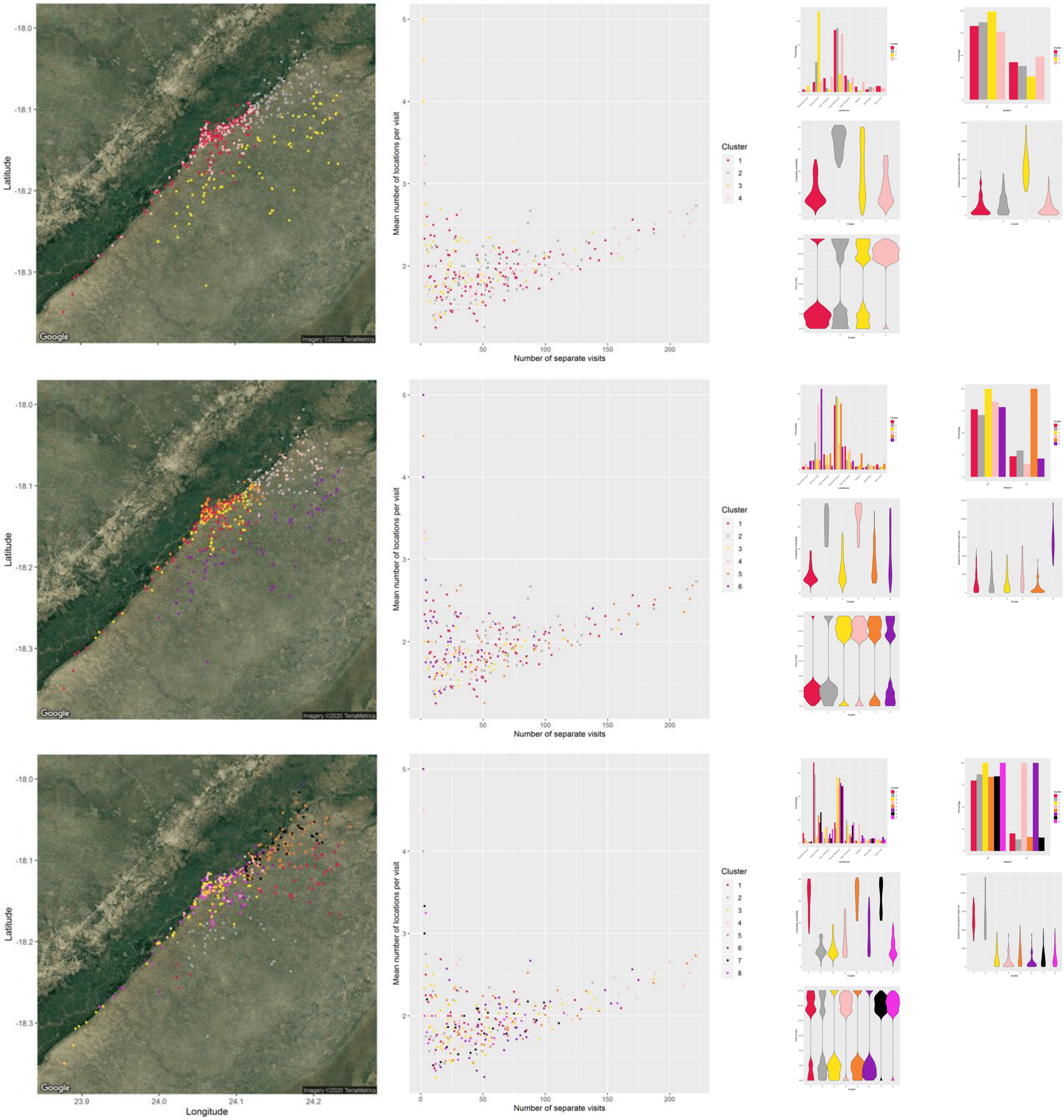

Latitude
Longitude
Google
Imagery ©2020 TerraMetrics
-18.0
-18.1
-18.2
-18.3
23.9
24.0
24.1
24.2
Mean number of locations per visit
Number of separate visits
0
50
100
150
200
Cluster
1
2
3
4
5
6
7
8

(d.1) BO-35947

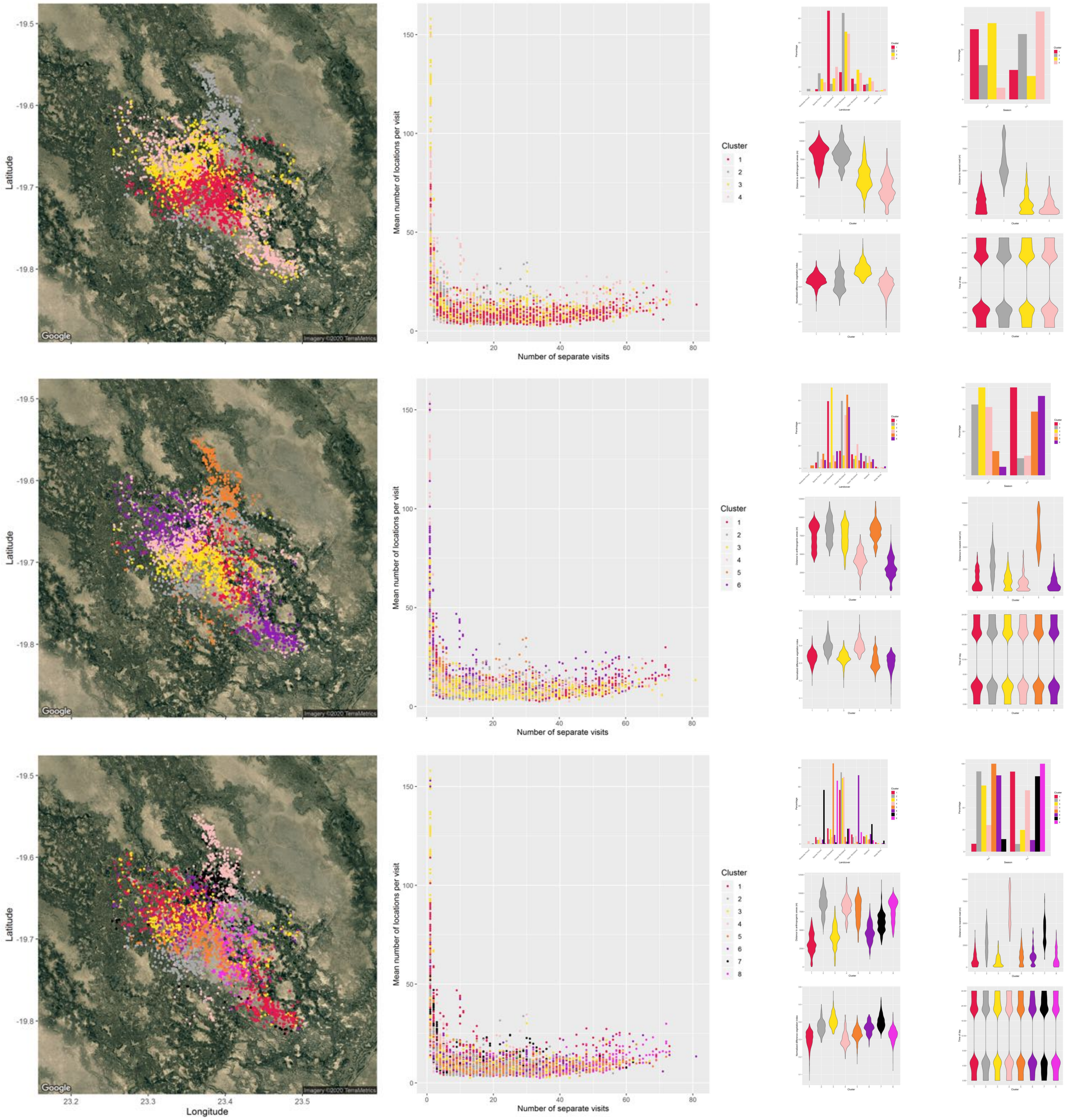

Latitude
Longitude
-19.5
-19.6
-19.7
-19.8
23.2
23.3
23.4
23.5
Google
Imagery ©2020 TerraMetrics
Mean number of locations per visit
Number of separate visits
0
20
40
60
80
50
100
150
Cluster
1
2
3
4
5
6
7
8

(d.2) BA-36715

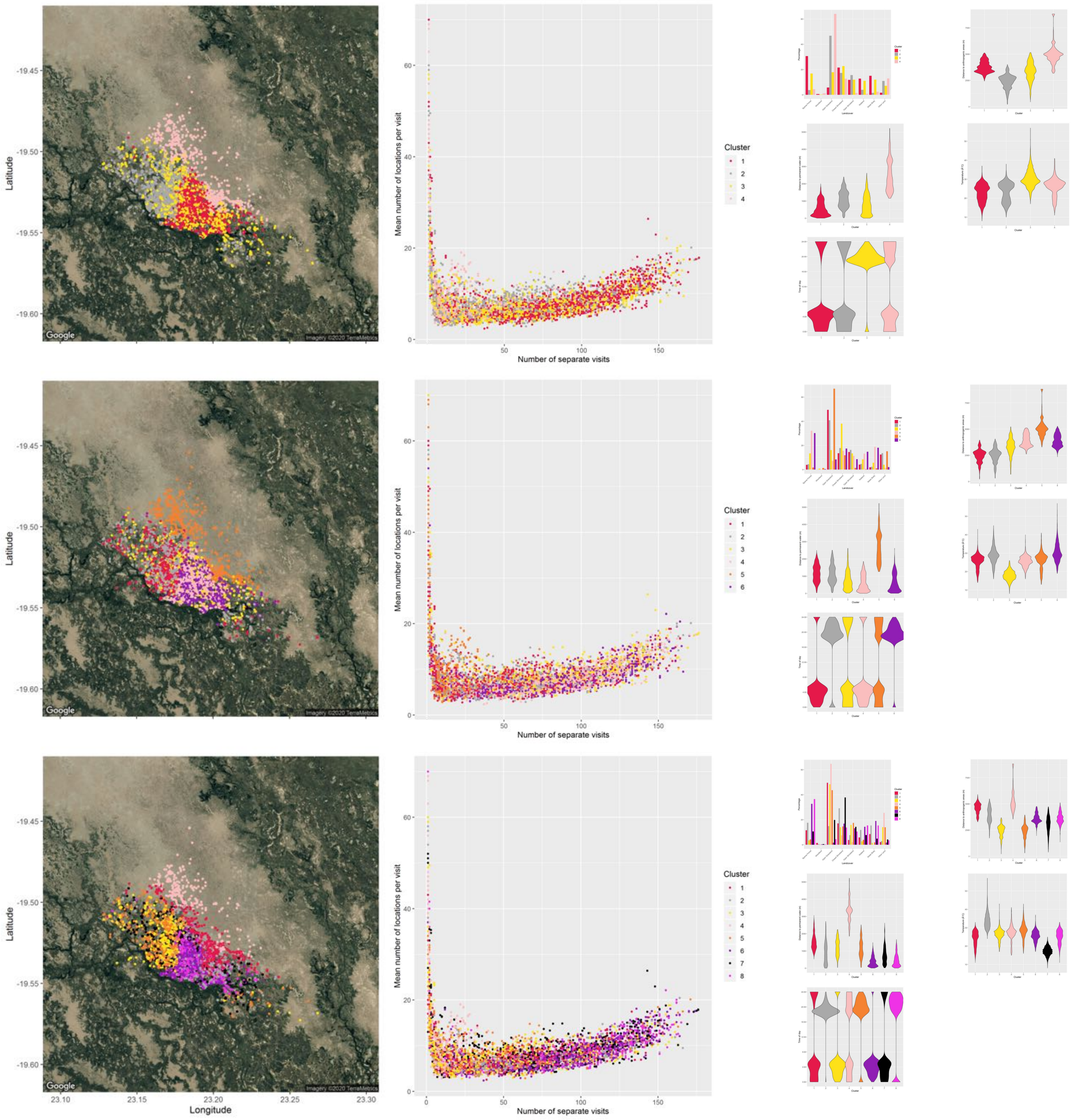

S18 Fig. Cluster analyses of lion revisitation and duration. Maps (left panels) depict the individual lion's relocations as four (top row), six (middle row), and eight (bottom row) clusters in the (a) Etosha National Park, Namibia; and (b) Chobe National Park; (c) Linyanti Conservancy; and (d) Okavango Delta, Botswana. Unique identifiers are depicted on top corner of each page. Relocations are colour-coded according to the clusters indicated by the range of revisitation (number of separate visits) and duration (mean number of locations per visit) values in RD space plots (shown in central panels). Clusters in the RD space were determined with the *k*-prototype algorithm and are based on ecogeographical variables attached to each relocation. The smaller plots (right panels) present the distribution and percent category of clusters for each of the ecogeographical variables selected from the factor analysis of mixed data (FAMD).
