# Supplementary material for: Coursing hyenas and stalking lions: The potential for inter- and intraspecific interactions
Source: PLoS One. 2023 Feb 3;18(2):e0265054. doi: 10.1371/journal.pone.0265054 (PMC9897591; doi:10.1371/journal.pone.0265054)
Supplement: S19 Fig — Maps (left panels) depict the individual spotted hyena’s relocations as four (top row), six (middle row), and eight (bottom row) clusters in the (a) Etosha National Park, Namibia; (b) Chobe National Park; and (c) Linyanti Conservancy, Botswana. Unique identifiers are depicted on top corner of each page. Relocations are color-coded according to the clusters indicated by the range of revisitation (number of separate visits) and duration (mean number of locations per visit) values in RD space plots (shown in central panels). Clusters in the RD space were determined with the k-prototype algorithm and are based on ecogeographical variables attached to each relocation. The smaller plots (right panels) present the distribution and percent category of clusters for each of the ecogeographical variables selected from the factor analysis of mixed data (FAMD). (PDF) [file pone.0265054.s035.pdf]

(a.1) GO-33869

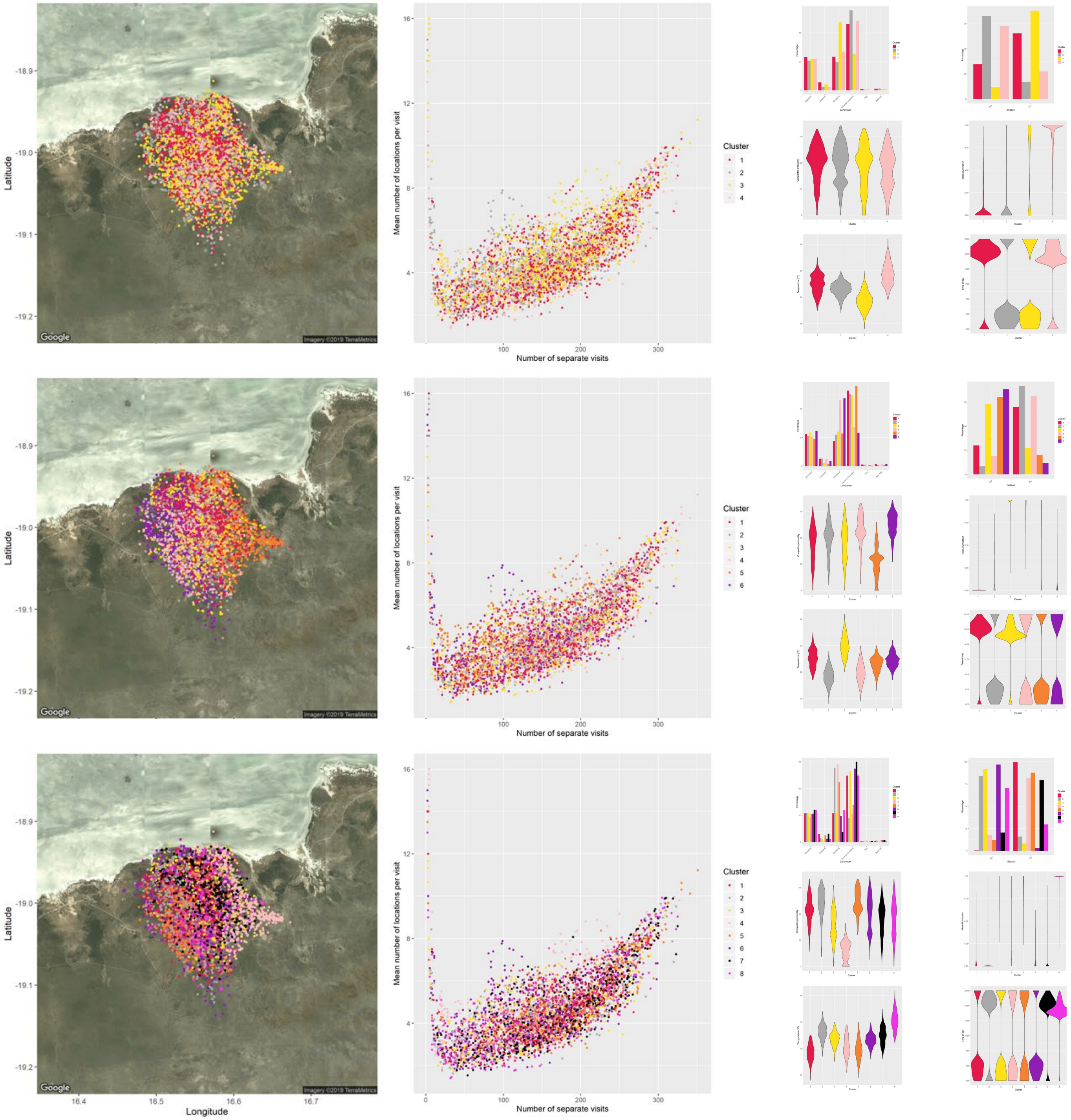
Latitude
Longitude
-18.9
-19.0
-19.1
-19.2
16.4
16.5
16.6
16.7
Google
Imagery ©2019 TerraMetrics
Mean number of locations per visit
Number of separate visits
0
100
200
300
4
8
12
16
Cluster
1
2
3
4
5
6
7
8

(a.2) TJ-33870

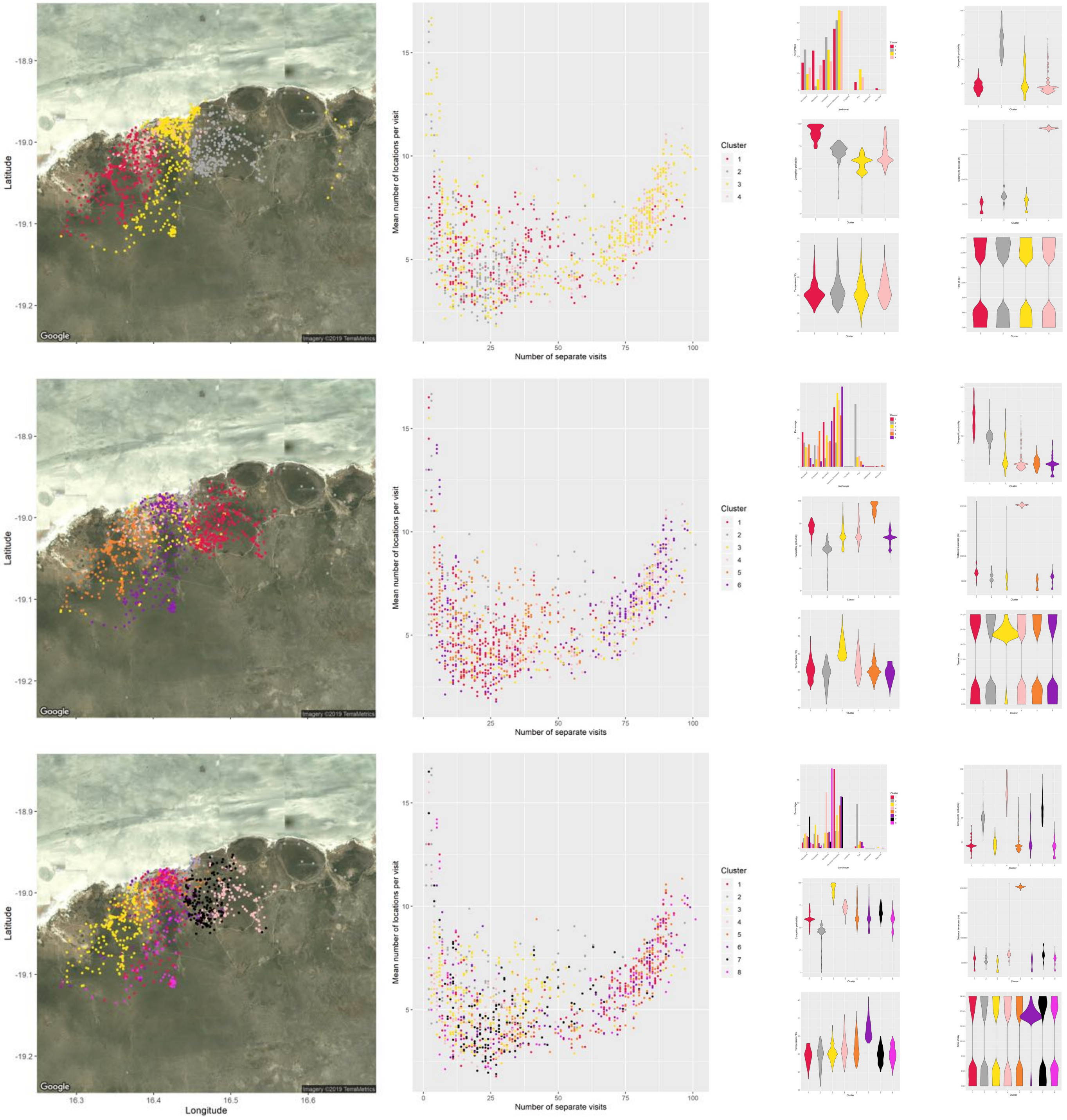

Latitude
-18.9
-19.0
-19.1
-19.2
Longitude
16.3
16.4
16.5
16.6
Google
Imagery ©2019 TerraMetrics
Mean number of locations per visit
15
10
5
Number of separate visits
0
25
50
75
100
Cluster
1
2
3
4
5
6
7
8

(a.3) NE-33871

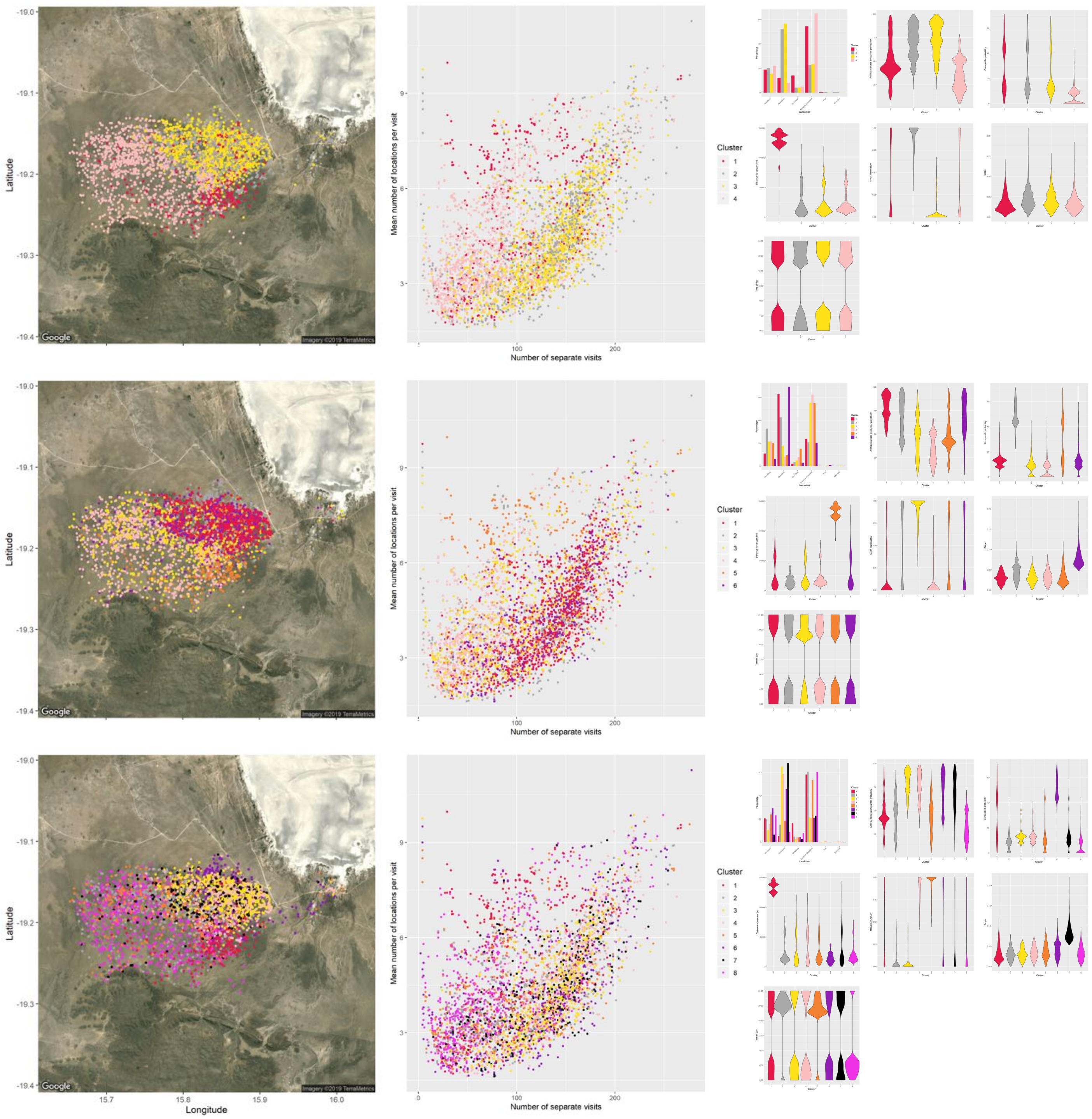

-19.0
-19.1
-19.2
-19.3
-19.4
Latitude
Google
Imagery ©2019 TerraMetrics
15.7
15.8
15.9
16.0
Longitude
Mean number of locations per visit
Number of separate visits
0
100
200
3
6
9
Cluster
1
2
3
4
5
6
7
8

(a.4) SA-33872

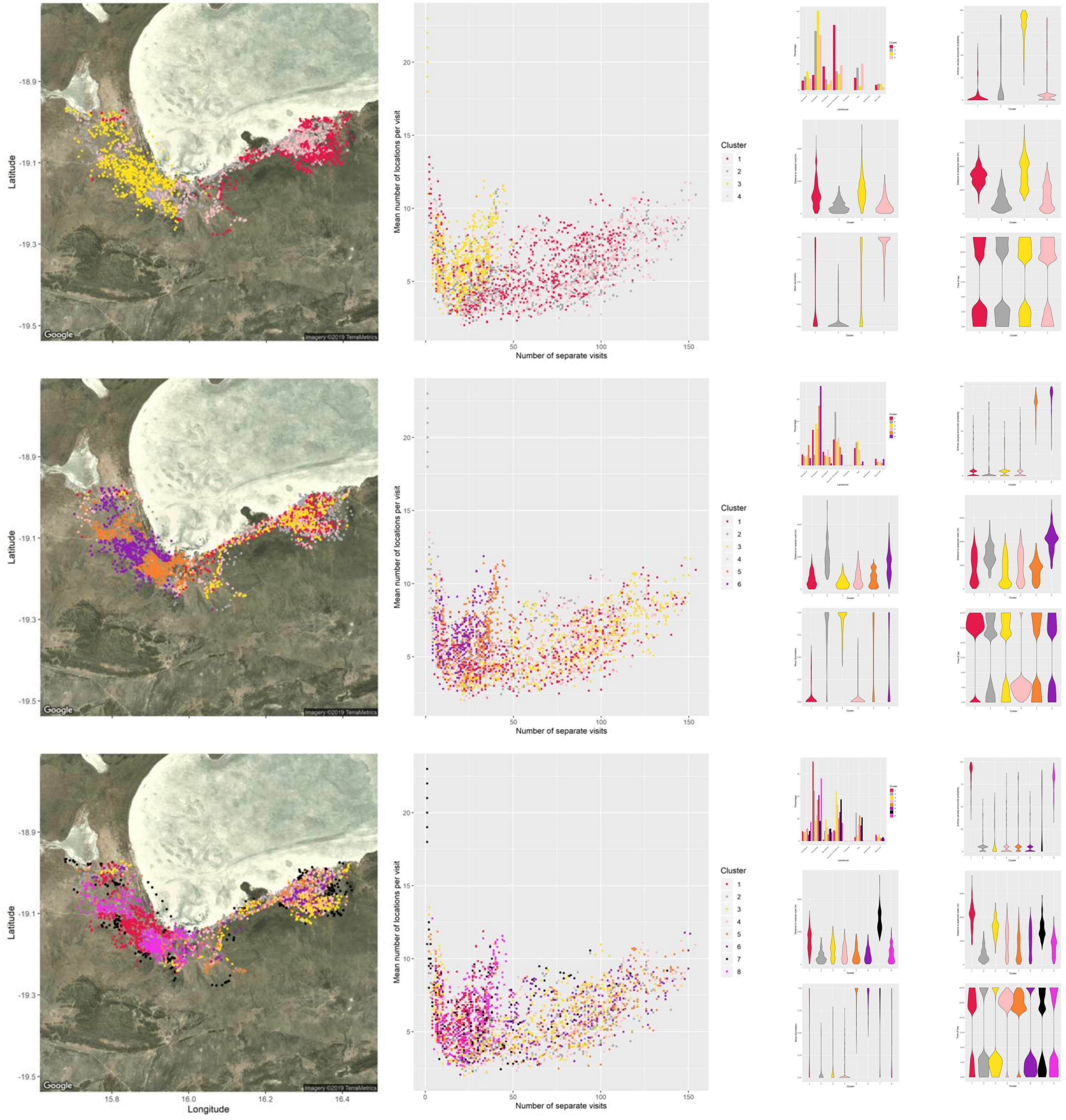
Latitude
-18.9
-19.1
-19.3
-19.5
Longitude
15.8
16.0
16.2
16.4
Google
Imagery ©2019 TerraMetrics
Mean number of locations per visit
5
10
15
20
Number of separate visits
0
50
100
150
Cluster
1
2
3
4
5
6
7
8

(a.5) AU-33873

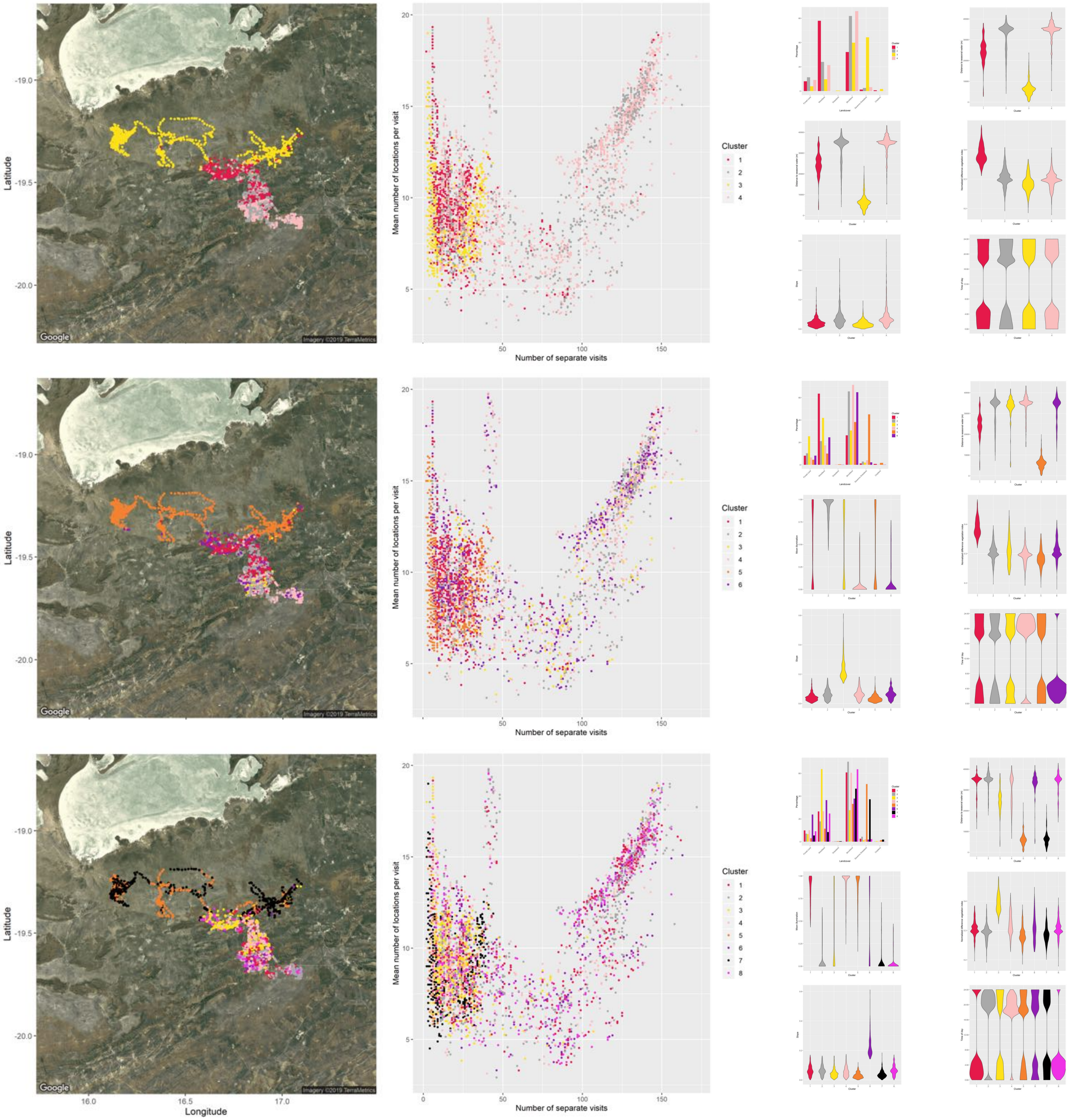

Latitude
-19.0
-19.5
-20.0
Longitude
16.0
16.5
17.0
Google
Imagery ©2019 TerraMetrics
Mean number of locations per visit
Number of separate visits
Cluster
1
2
3
4
5
6
7
8

## (a.6) OM-33874

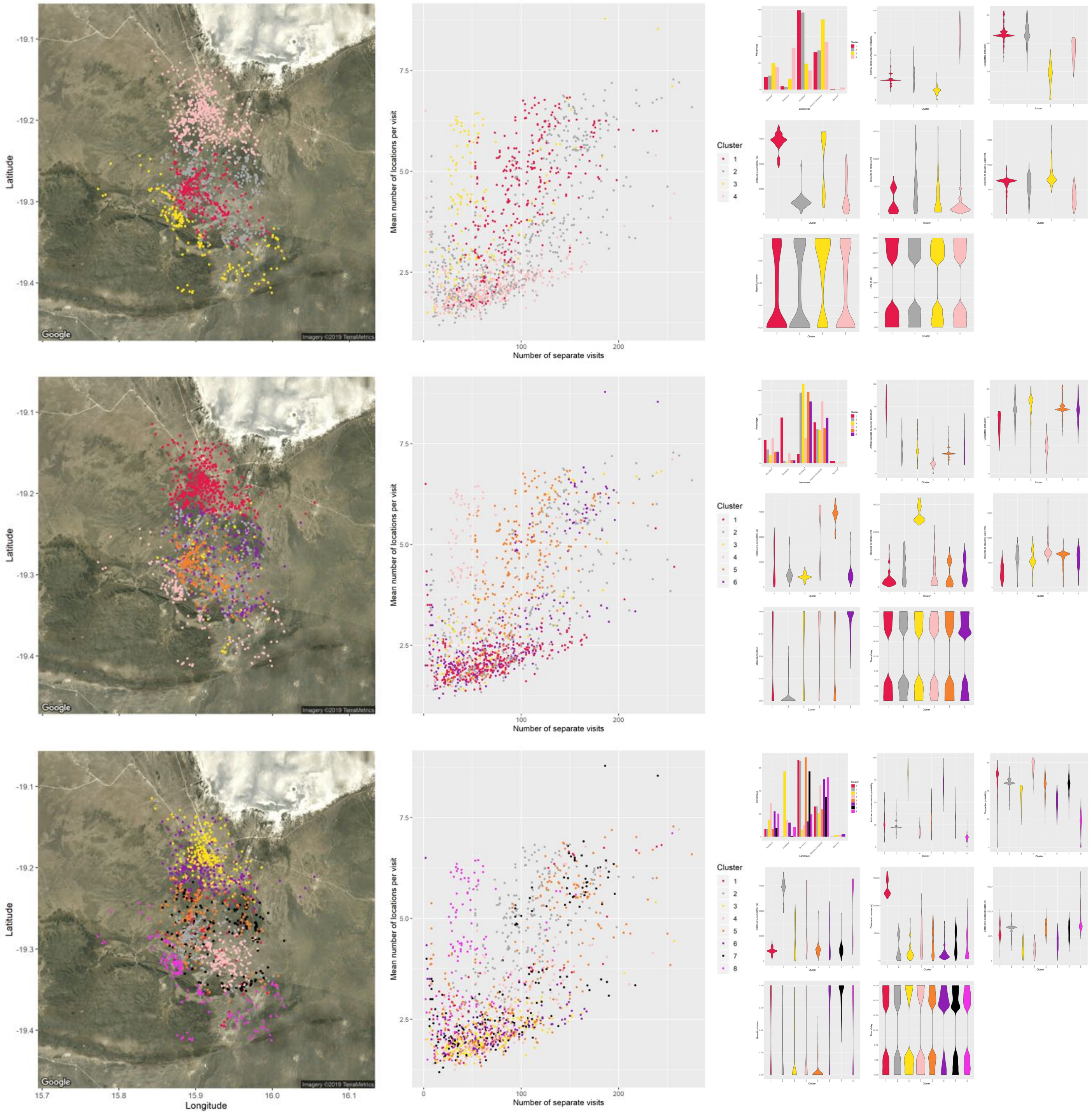
Latitude
-19.1
-19.2
-19.3
-19.4
Longitude
15.7
15.8
15.9
16.0
16.1
Google
Imagery ©2019 TerraMetrics
Mean number of locations per visit
Number of separate visits
7.5
5.0
2.5
0
100
200
Cluster
1
2
3
4
5
6
7
8

(a.7) SU-33951

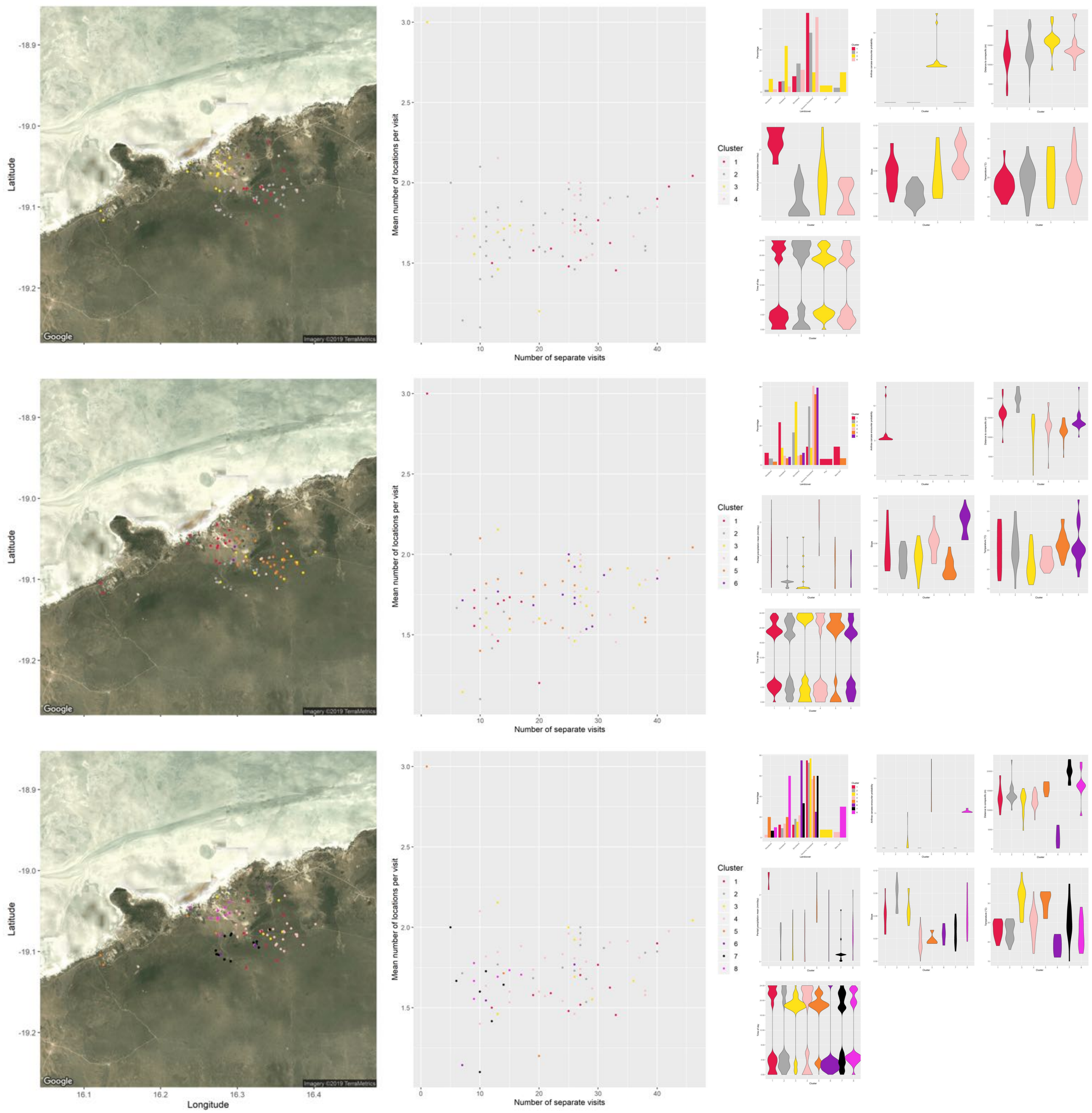

Latitude
-18.9
-19.0
-19.1
-19.2
Google
Imagery ©2019 TerraMetrics
16.1
16.2
16.3
16.4
Longitude
Mean number of locations per visit
3.0
2.5
2.0
1.5
0
10
20
30
40
Number of separate visits
Cluster
1
2
3
4
5
6
7
8

(a.8) WO-34310

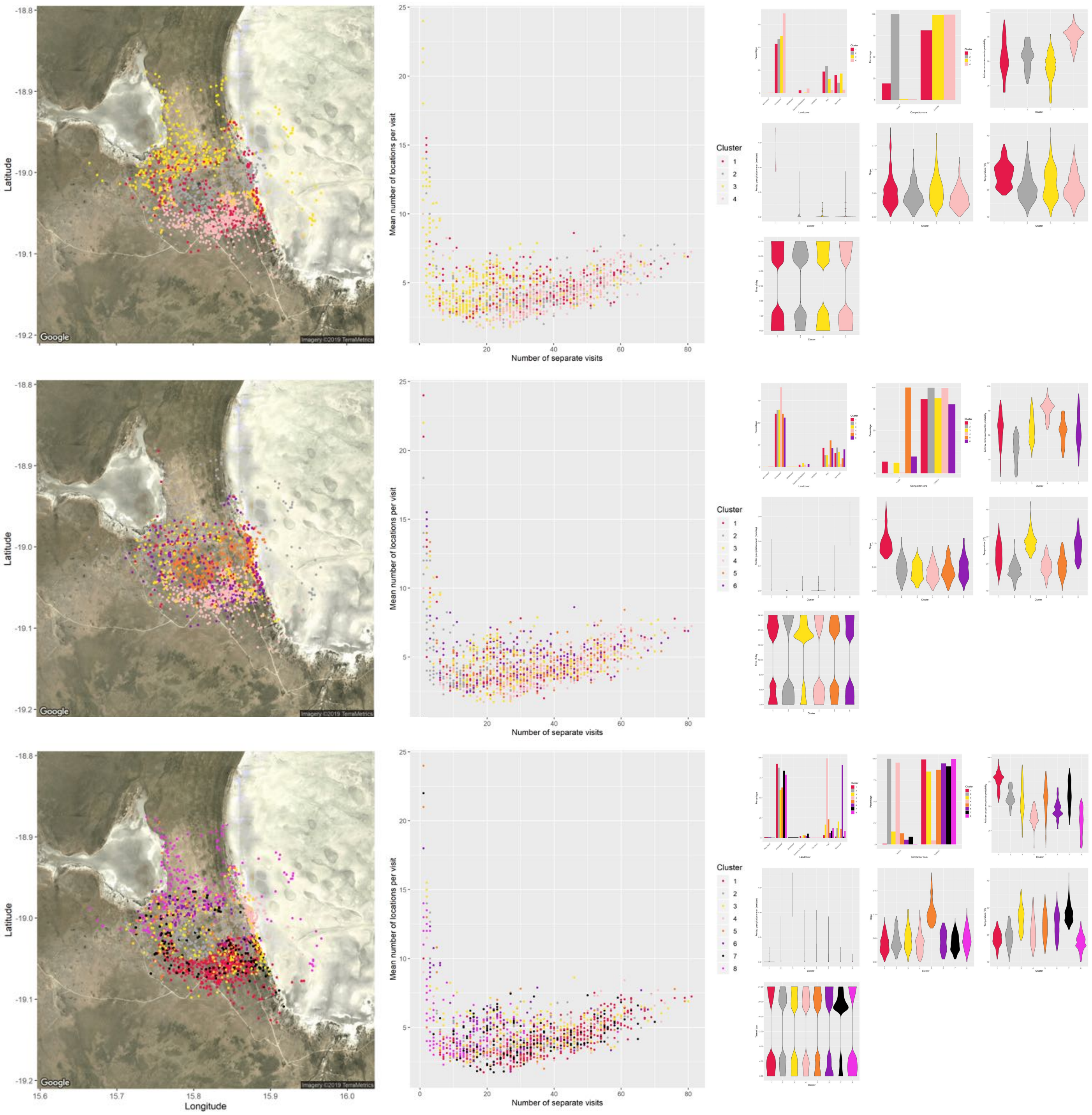

Latitude
-18.8
-18.9
-19.0
-19.1
-19.2
Google
Imagery ©2019 TerraMetrics
15.6
15.7
15.8
15.9
16.0
Longitude
Mean number of locations per visit
Number of separate visits
Cluster
1
2
3
4
5
6
7
8

(b.1) IH-33870

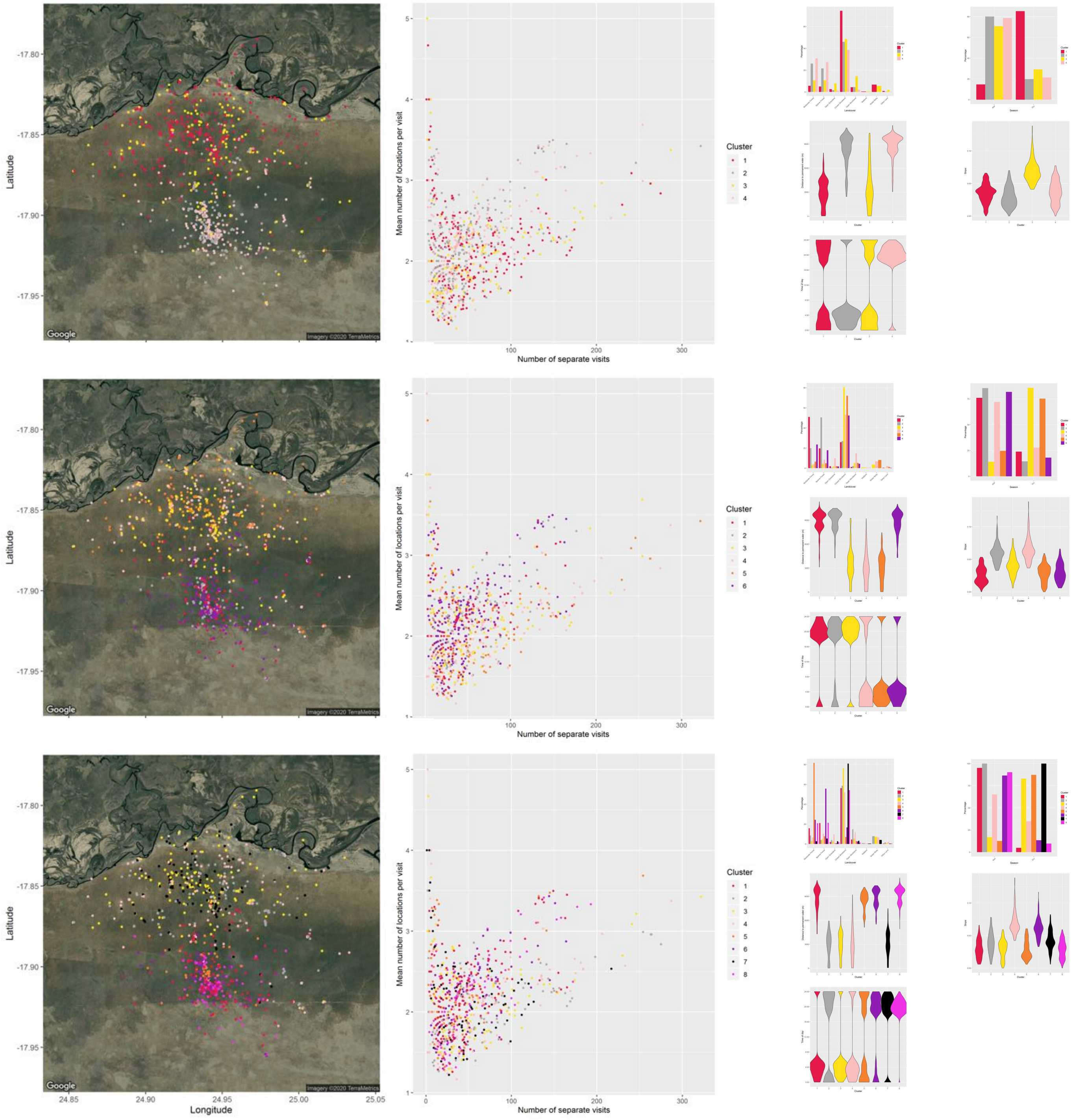
Latitude
Longitude
Mean number of locations per visit
Number of separate visits
Cluster
Google
Imagery ©2020 TerraMetrics

(b.2) KW-33871

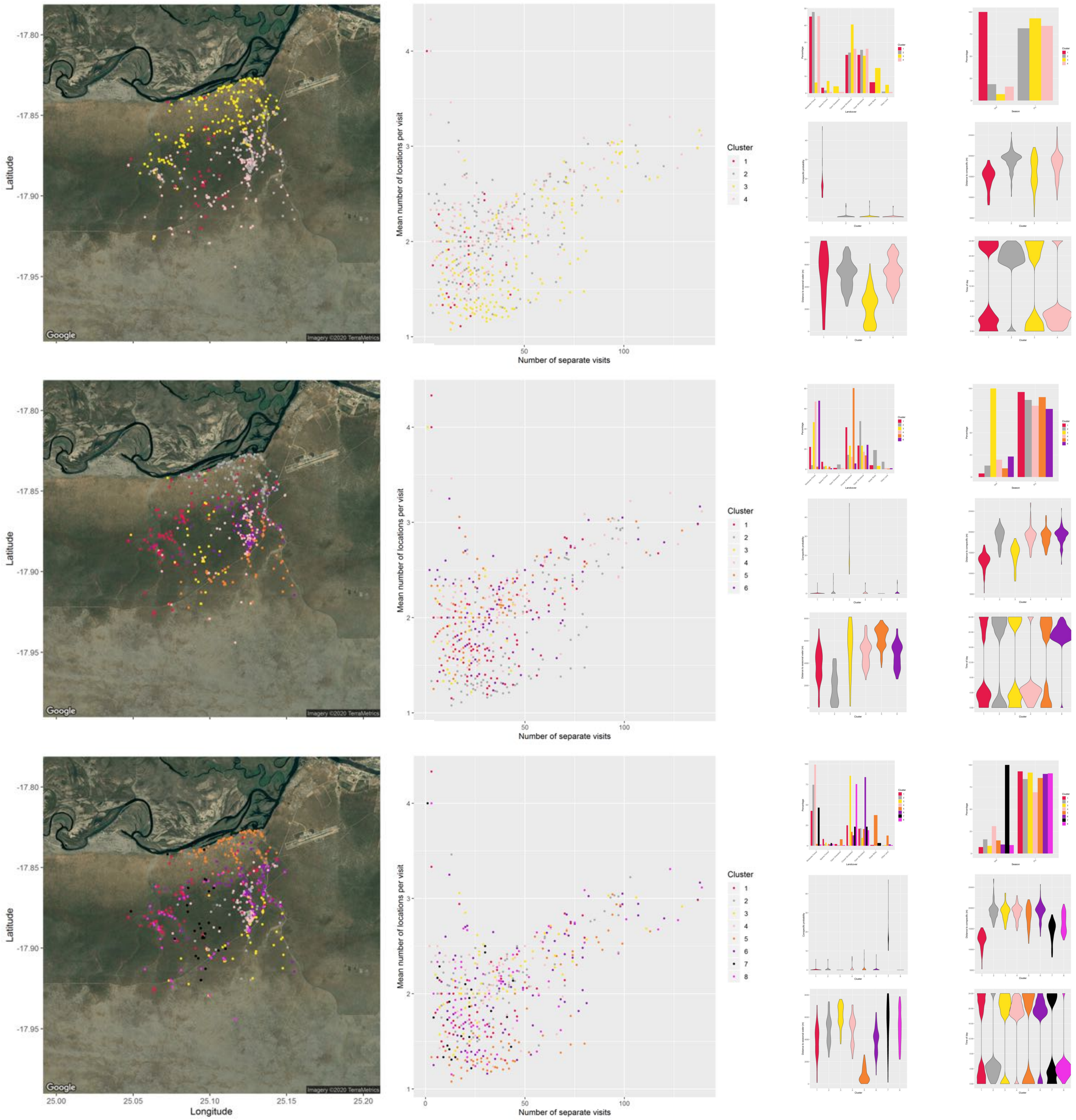
Latitude
Longitude
Google
Imagery ©2020 TerraMetrics
-17.80
-17.85
-17.90
-17.95
25.00
25.05
25.10
25.15
25.20
Mean number of locations per visit
Number of separate visits
0
50
100
Cluster
1
2
3
4
5
6
7
8

# (b.3) SR-34310

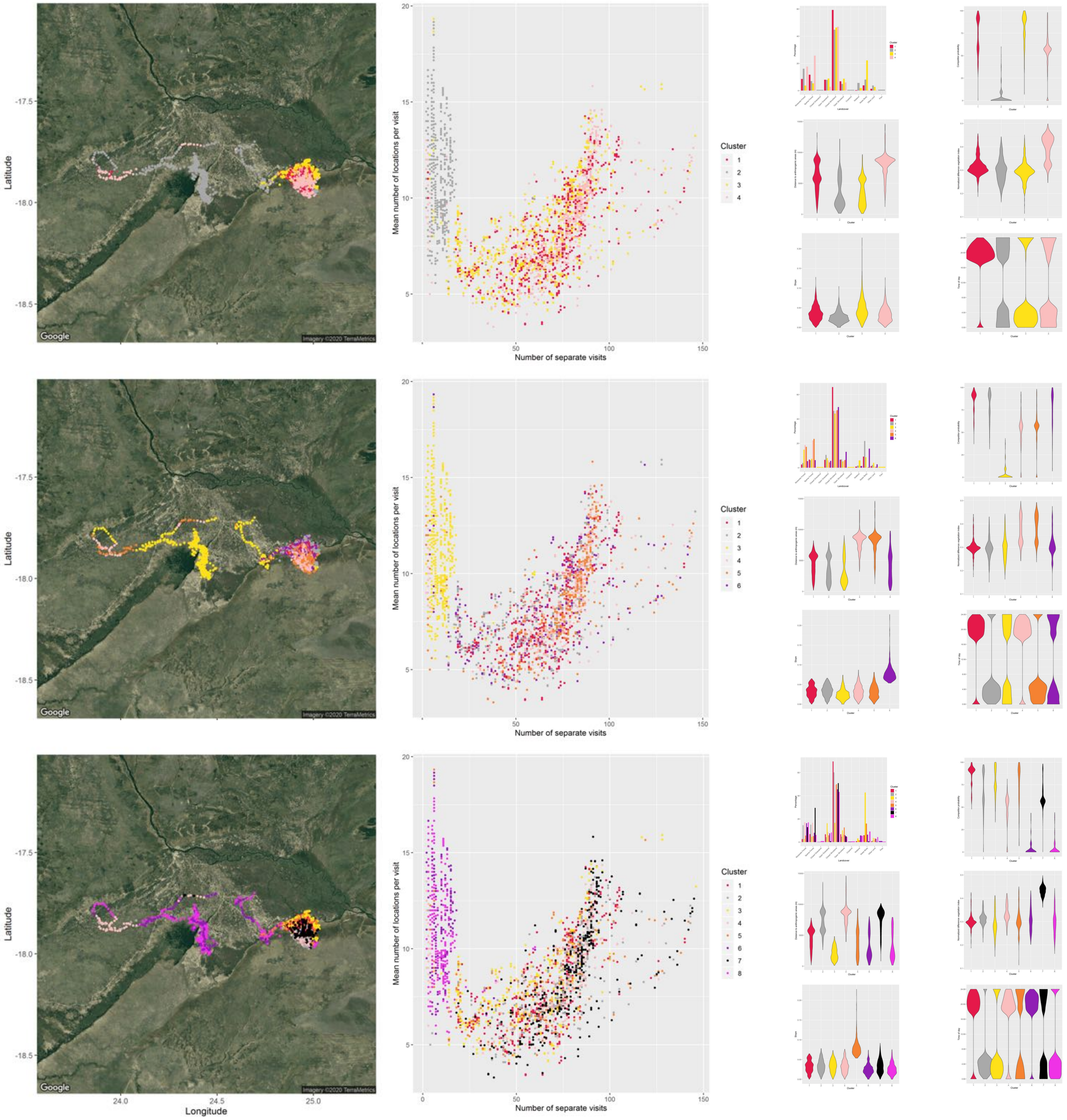
Latitude
Longitude
Mean number of locations per visit
Number of separate visits
Cluster
Google
Imagery ©2020 TerraMetrics

(c.1) AR-33869

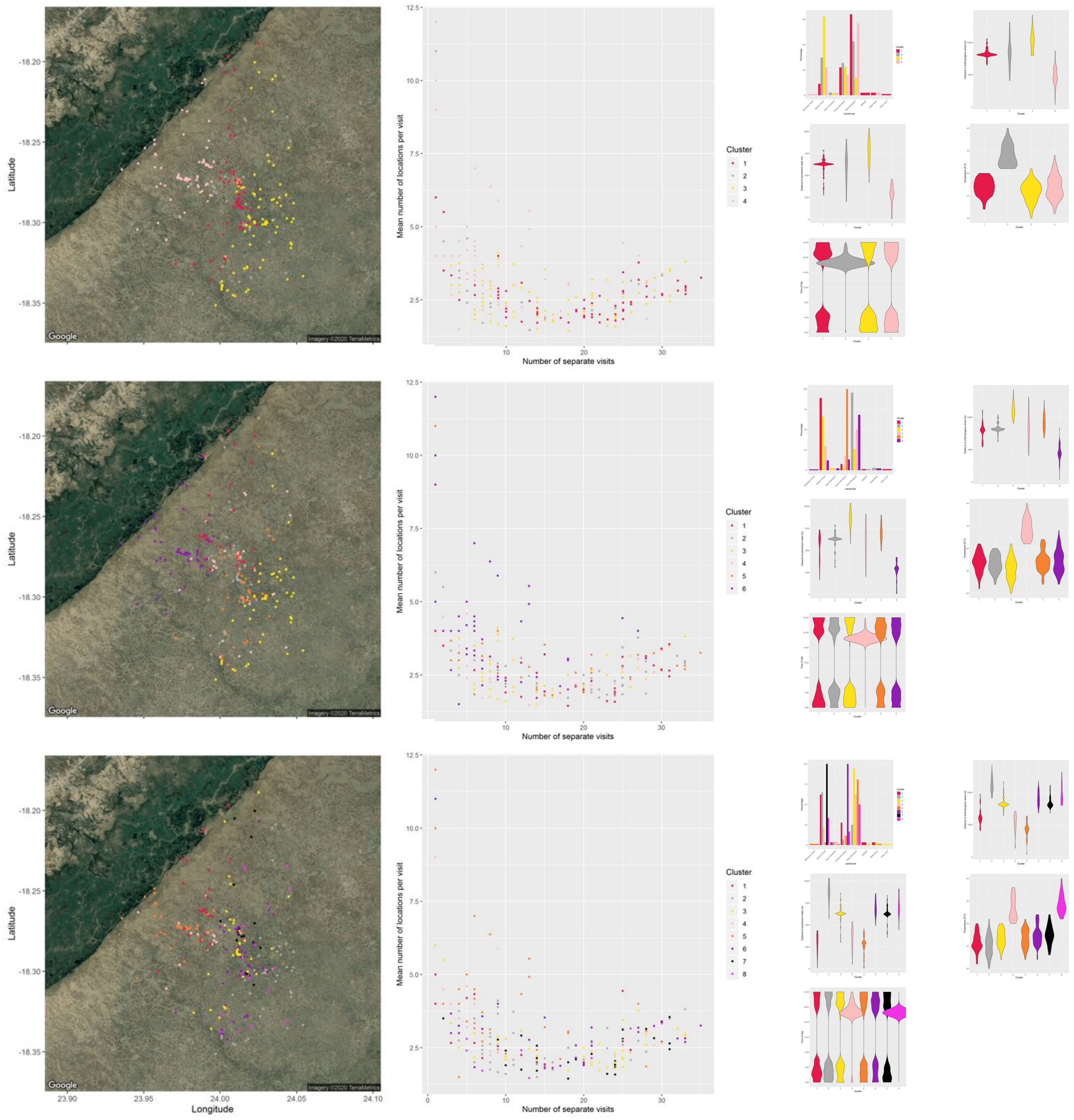

Latitude
-18.20
-18.25
-18.30
-18.35
Google
Imagery ©2020 TerraMetrics
23.90
23.95
24.00
24.05
24.10
Longitude
Mean number of locations per visit
12.5
10.0
7.5
5.0
2.5
0
10
20
30
Number of separate visits
Cluster
1
2
3
4
5
6
7
8

(c.2) RV-33873

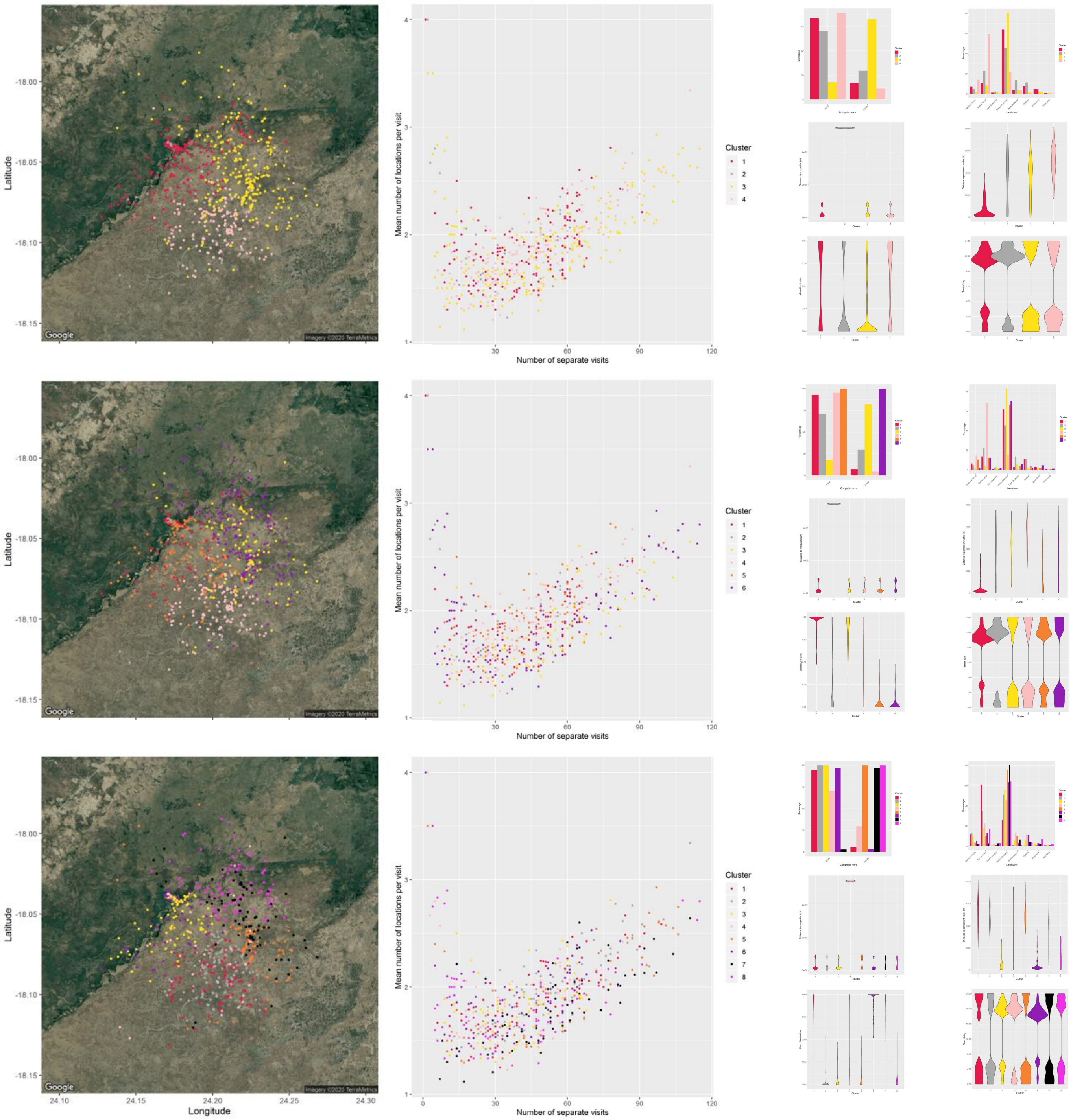


**S19 Fig. Cluster analyses of spotted hyena revisitation and duration.** Maps (left panels) depict the individual spotted hyena's relocations as four (top row), six (middle row), and eight (bottom row) clusters in the (a) Etosha National Park, Namibia; (b) Chobe National Park; and (c) Linyanti Conservancy, Botswana. Unique identifiers are depicted on top corner of each page. Relocations are colour-coded according to the clusters indicated by the range of revisitation (number of separate visits) and duration (mean number of locations per visit) values in RD space plots (shown in central panels). Clusters in the RD space were determined with the *k*-prototype algorithm and are based on ecogeographical variables attached to each relocation. The smaller plots (right panels) present the distribution and percent category of clusters for each of the ecogeographical variables selected from the factor analysis of mixed data (FAMD).
