# Supplementary material for: Coursing hyenas and stalking lions: The potential for inter- and intraspecific interactions
Source: PLoS One. 2023 Feb 3;18(2):e0265054. doi: 10.1371/journal.pone.0265054 (PMC9897591; doi:10.1371/journal.pone.0265054)
Supplement: S20 Fig — Linear regression results relating the mean activity of (a) lions and (b) spotted hyenas to temperature (°C) over each hour of the 24-hour cycle. Each panel indicates the time interval with its’ adjusted R2 and p-value. The red line is the line of best fit to the data, with the grey shaded bars the 95% confidence interval. An asterisk denotes significance at the alpha level with * < 0.05, and *** < 0.001. (PDF) [file pone.0265054.s036.pdf]

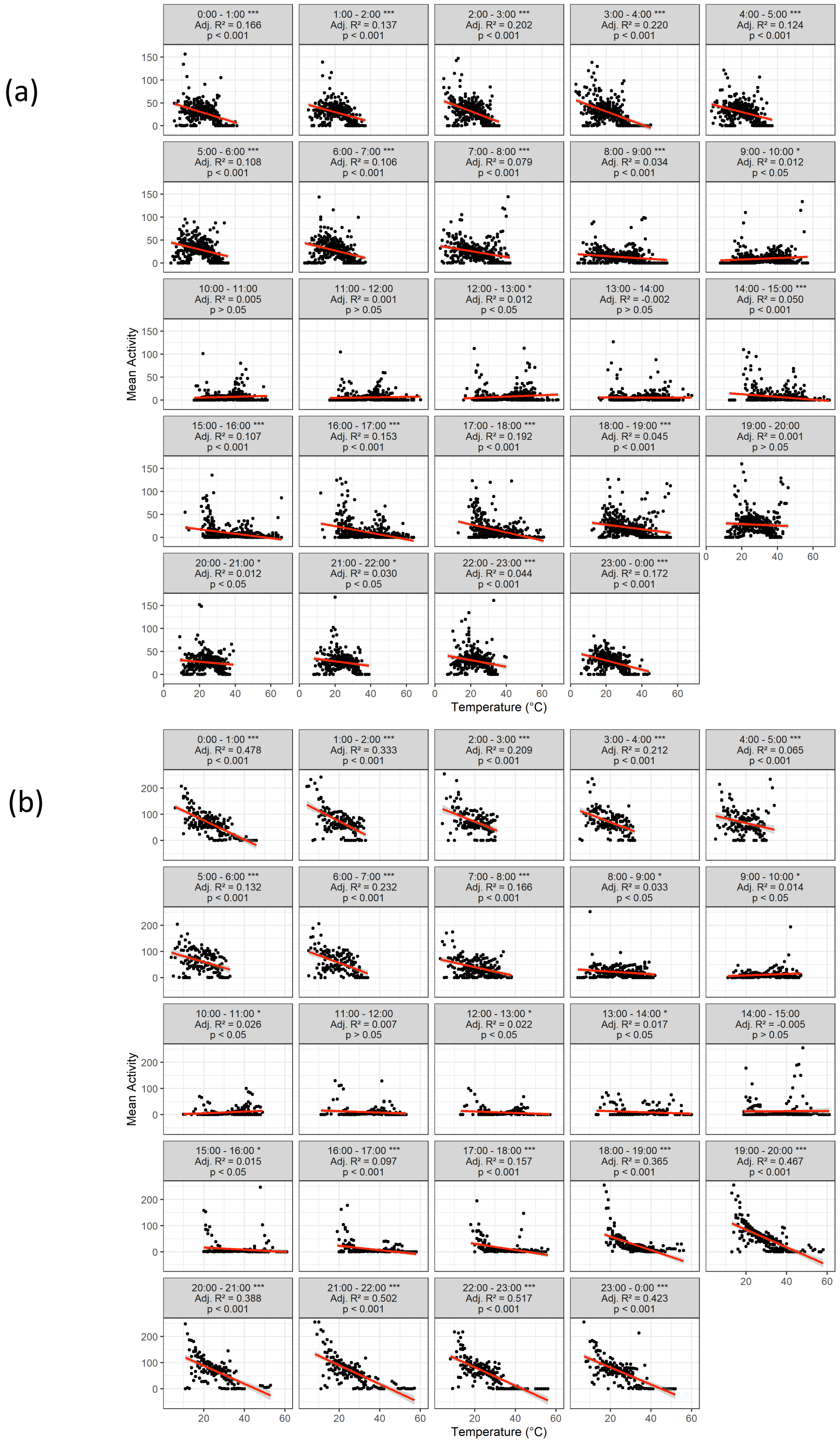

S20 Fig. Relationship plots of lion and spotted hyena activity in relation to temperature for each hour of the 24 hour cycle. Linear regression results relating the mean activity of (a) lions and (b) spotted hyenas to temperature (°C) over each hour of the 24 hour cycle. Each panel indicates the time interval with its' adjusted R^2 and *p*-value. The red line is the line of best fit to the data, with the grey shaded bars the 95% confidence interval. An asterisk denotes significance at the alpha level with * < 0.05, and *** < 0.001.
