# Supplementary material for: Coursing hyenas and stalking lions: The potential for inter- and intraspecific interactions
Source: PLoS One. 2023 Feb 3;18(2):e0265054. doi: 10.1371/journal.pone.0265054 (PMC9897591; doi:10.1371/journal.pone.0265054)
Supplement: Attachment — Submitted filename: Response to Reviewers.pdf [file pone.0265054.s037.pdf]

Dr Marco Apollonio,
Academic Editor
PLOS ONE

November 18, 2022

Dear Dr Apollonio,

Please accept this letter of rebuttal as our response to the reviewers. We appreciated the valuable comments from the reviewers', as they highlighted areas of the manuscript that were hard to follow in our attempts to provide this information to the readers. By addressing these concerns raised by the reviewers, we agree that removing the descriptive statistics from the text to be condensed into tables has resulted in an easier flow in that the text reads clearer.

We feel that this has strengthened the manuscript considerably. We thank the reviewers for their valuable comments and for their time and assistance in improving this manuscript.

Please note that the reviewer's comments have been italicized, and our responses are inserted below in bold.

1. Please ensure that your manuscript meets PLOS ONE's style requirements, including those for file naming. The PLOS ONE style templates can be found at https://journals.plos.org/plosone/s/file?id=wjVg/PLOSOne_formatting_sample_main_body.pdf and https://journals.plos.org/plosone/s/file?id=ba62/PLOSOne_formatting_sample_title_authors_affiliations.pdf

We have redone the file naming system and corrected this to ensure that our manuscript now meets PLOS ONE's style requirements for file naming.

2. We note that you have stated that you will provide repository information for your data at acceptance. Should your manuscript be accepted for publication, we will hold it until you provide the relevant accession numbers or DOIs necessary to access your data. If you wish to make changes to your Data Availability statement, please describe these changes in your cover letter and we will update your Data Availability statement to reflect the information you provide.

**We provided in our submission the repository information for which our data is held in, within the Zenodo repository and can be found here:
Zenodo: https://doi.org/10.5281/zenodo.5949231**

3. We note that you have referenced (ie. Bewick et al. [5]) which has currently not yet been accepted for publication. Please remove this from your References and amend this to state in the body of your manuscript: (ie "Bewick et al. [Unpublished]") as detailed online in our guide for authors http://journals.plos.org/plosone/s/submission-guidelines#loc-reference-style

We do not find the reference Bewick et al. in our paper. Our reference [5] is for Scognamillo et al., and we have checked through our paper for any reference to Bewick and have found none.

4. We note that Figure 1 in your submission contain [map/satellite] images which may be copyrighted. All PLOS content is published under the Creative Commons Attribution License (CC BY 4.0), which means that the manuscript, images, and Supporting Information files will be freely available online, and any third party is permitted to access, download, copy, distribute, and use these materials in any way, even commercially, with proper attribution. For these reasons, we cannot publish previously copyrighted maps or satellite images created using proprietary data, such as Google software (Google Maps, Street View, and Earth). For more information, see our copyright guidelines: http://journals.plos.org/plosone/s/licenses-and-copyright.

We require you to either (1) present written permission from the copyright holder to publish these figures specifically under the CC BY 4.0 license, or (2) remove the figures from your submission:

a. You may seek permission from the original copyright holder of Figure 1 to publish the content specifically under the CC BY 4.0 license.

We recommend that you contact the original copyright holder with the Content Permission Form (http://journals.plos.org/plosone/s/file?id=7c09/content-permission-form.pdf) and the following text:
"I request permission for the open-access journal PLOS ONE to publish XXX under the Creative Commons Attribution License (CCAL) CC BY 4.0 (http://creativecommons.org/licenses/by/4.0/). Please be aware that this license allows unrestricted use and distribution, even commercially, by third parties. Please reply and provide explicit written permission to publish XXX under a CC BY license and complete the attached form."

Please upload the completed Content Permission Form or other proof of granted permissions as an "Other" file with your submission.

In the figure caption of the copyrighted figure, please include the following text: "Reprinted from [ref] under a CC BY license, with permission from [name of publisher], original copyright [original copyright year]."

b. If you are unable to obtain permission from the original copyright holder to publish these figures under the CC BY 4.0 license or if the copyright holder's requirements are incompatible with the CC BY 4.0 license, please either i) remove the figure or ii) supply a replacement figure that complies with the CC BY 4.0 license. Please check copyright information on all replacement figures and update the figure caption with source information. If applicable, please specify in the figure caption text when a figure is similar but not identical to the original image and is therefore for illustrative purposes only.
The following resources for replacing copyrighted map figures may be helpful:

USGS National Map Viewer (public domain): http://viewer.nationalmap.gov/viewer/
The Gateway to Astronaut Photography of Earth (public domain): http://eol.jsc.nasa.gov/sseop/clickmap/
Maps at the CIA (public domain): https://www.cia.gov/library/publications/the-world-factbook/index.html and https://www.cia.gov/library/publications/cia-maps-publications/index.html
NASA Earth Observatory (public domain): http://earthobservatory.nasa.gov/
Landsat: http://landsat.visibleearth.nasa.gov/
USGS EROS (Earth Resources Observatory and Science (EROS) Center) (public domain): http://eros.usgs.gov/#
Natural Earth (public domain): http://www.naturalearthdata.com/

We created Fig 1 ourselves using ArcGIS (ESRI ArcMap v.10.0). The previous PDF of Fig 1 uploaded to PLOS ONE was mistakenly created using the screenshot tool instead of exporting as PDF directly from ArcGIS. This resulted in lines around the map, which made it look as if had been pasted from somewhere else. We have since corrected this error by exporting directly from ArcGIS as PDF.

5. Please include your tables as part of your main manuscript and remove the individual files. Please note that supplementary tables (should remain/ be uploaded) as separate "supporting information" files.

Table 1 has now been included into the main manuscript, and the individual files have been removed.

6. Please review your reference list to ensure that it is complete and correct. If you have cited papers that have been retracted, please include the rationale for doing so in the manuscript text, or remove these references and replace them with relevant current references. Any changes to the reference list should be mentioned in the rebuttal letter that accompanies your revised manuscript. If you need to cite a retracted article, indicate the article's retracted status in the

References list and also include a citation and full reference for the retraction notice.

We have reviewed our reference list to ensure that it is complete and correct.

Reviewer #1: I really liked your paper and feel it presents really important information from a great dataset and hence recommended minor revisions. That said, it was a pretty tough read - very long and filled with data that I think would be better served being presented as tables or figures (I realise there are a lot of these already - but they help your audience out a lot). I think this would help with the flow of the manuscript also. Hence, my big suggestion would be to try to move some of the Results text into tables, and abbreviate the manuscript wherever you can.

We have created new tables (Tables 3, 4, and 7) and included significant values (into Tables 5, 6, and 8) taken from supplementary materials to replace approximately 4 pages of text in the Results section.

Specific suggestions:
- L61: I'd argue that these species 'preferentially' forage on different types of food, but opportunism masks these trends to a degree (Hayward, M. W. and Kerley, G. I. H. 2008. Prey preferences and dietary overlap amongst Africa's large predators. - SA J. Wildl. Res. 38: 93-108.).

We have changed "More specifically, species whose ranges overlap forage different types of food" to "More specifically, species whose ranges overlap forage different types of food, either preferentially or opportunistically" (L64) to reflect this, and included the reference to Hayward and Kerley.

- L326: Can you add in the years that these data were collected here again please?

We have added in the years that the data were collected here (L343-351).

- L498: Somewhere around here, I'd be interested in hearing about how the core areas were situated in relation to those of their competitor (i.e., distances from centre of core areas or close to territory boundaries)?

We included this information at the end of this section (L522-523) and with a new table in supplementary materials (S6 Table) in where we said:

“Furthermore, individual core areas were more distant from competitor core areas than to the nearest territory boundaries of competitors’. This was significant for most cases (all t-tests $p < 0.05$), except for hyenas to heterospecific competitors in Etosha, and to conspecific competitors in Botswana.” and referred to the new table in the supplementary materials.

- L555: Change to "Moreover, lion and hyena movements and activity differed according to the..."

We changed “Moreover, lion and hyena movements were found to differ according to the …” to “Moreover, lion and hyena movements and activity differed according to the …” (L585) as recommended.

- L596-608: Can you relate movements to temperatures as well (each hour of the day)? Seems you've tested one potential explanation for activity, but ignored this one that Rob and I used to explain the strict nocturnality of hyaenas.

We have added in L556-560 a section indicating the relationship between lion and hyena activity to temperature, in which we also provided the adjusted R^2 and included this information in a new figure in S20 Fig in supplementary materials.

- L603: Is this clearer - "Despite lions and hyaenas exhibiting higher proportions of activity during certain periods of the night..."

We changed “Despite whether lions and hyenas were exhibiting higher proportions of activity during certain periods of the night” to “Despite lions and hyenas exhibiting higher proportions of activity during certain periods of the night” (L652) as recommended.

- L793 and stats throughout: always present the chi-square value, alongside degrees of freedom and the p-value.

We have presented the chi-square values alongside with the degrees of freedom and p-values throughout the manuscript where mentioned (i.e., L666, L668, L669), including the tables and in supplementary materials.

- L832: "As mainly cursorial predators, spotted hyaenas ..."

We changed “Mainly cursorial predators, spotted hyenas …” to “As mainly cursorial predators, spotted hyenas” (L865) as recommended.

- L834-836: Isn't this a chicken and egg issue? Are they using those habitats because they like those habitats or their prey likes those habitats?

We state here that lions and spotted hyenas within this study were utilizing similar habitats, not whether they or their prey likes those habitats, and referenced this to the study of Durant et al., 2010 which found that lions and spotted hyenas exhibited broad overlap in terms of habitat preferences, regardless of prey (L867-869).

- L838: isn't preferred prey an important factor here alongside habitat?

We agree prey is an important factor for the distribution and movements of carnivores, and we include this later in our discussion L888-899 where we state:

> **“Previous studies have demonstrated lion movements to be inextricably tied to the location of water-holes across the landscape due to concentrated search efforts for prey [25,118]. …” and later “Spotted hyenas have been found to use locations far from water for den sites and resting [30,114], thus we suggest that hyenas are potentially choosing to remain in areas with landscape characteristics that minimizes detection [120] while increasing prey vulnerability [121].”**

In the earlier section of the discussion, we focus here on the differences in the use of similar habitats between the two species, regardless of prey.

- L844-846: Kasim Rafiq found something similar with leopards and the African carnivore guild as well.

We have included this recent reference here (L877-879).

We thank the reviewers again for their support and guidance with this manuscript.

Yours sincerely,

Nancy Barker

Ph.D., School of Life Sciences
University of KwaZulu-Natal, Westville campus
Durban 4041, South Africa
Email: nancybarkerzoologist@gmail.com
